# Evidence for volcanic forcing of Holocene cold events

**DOI:** 10.1038/s41467-026-73492-4

**Published:** 2026-05-28

**Authors:** Alice R. Paine, James U. L. Baldini, Charlie L. Rex, Michael Sigl, Francesco S. R. Pausata, Richard J. Brown

**Affiliations:** 1 Department of Environmental Sciences, University of Basel, Basel, Switzerland; 2 Department of Earth Sciences, Durham University, Durham, UK; 3 Department of Earth Sciences, University of Oxford, Oxford, UK; 4 Department of Geosciences, University of Oslo, Oslo, Norway; 5 Oeschger Centre for Climate Change Research, University of Bern, Bern, Switzerland; 6 Centre ESCER (Étude et la Simulation du Climat à l’Échelle RÉgionale) and GEOTOP (Research Centre on the dynamics of the Earth System), Department of Earth and Atmospheric Sciences, University of Quebec in Montréal, Montréal, Canada

**Keywords:** Palaeoclimate, Natural hazards

## Abstract

During the Holocene, the climatic stability of the northern hemisphere was intermittently disrupted by centennial-scale cooling events, whose origins remain unclear. Explosive volcanism is a plausible trigger, yet its potential to drive longer-term (centennial-to-millennial) perturbations remains underexplored. Here, we compile records of explosive volcanism, atmospheric sulphate, climate variability, and glacial dynamics over the past ~12,000 years to test the temporal correspondence between major eruptions and abrupt cooling events, and assess mechanisms linking volcanic forcing to prolonged climatic shifts. Over 80% of Holocene glacial advances occurred within chronological uncertainty of at least one large (M ≥ 7) eruption in the northern hemisphere. Monte Carlo simulations confirm that this relationship is non-random (p < 0.01). Combined with evidence for sea ice expansion, Atlantic Ocean circulation weakening, and southward tropical rain belt displacement, our results suggest that volcanic impacts can persist well beyond aerosol lifetimes, emphasizing the need to consider dynamical feedbacks in Earth system responses to eruptions.

## Abstract (web summary)

This study shows that over 80% of Holocene glacier advances coincide with major eruptions, suggesting explosive volcanism could trigger long-lasting cooling through feedbacks involving the ocean, sea ice and atmospheric circulation.

## Introduction

The Holocene is the current interglacial of the Quaternary period and began ~11,700 years ago with a rapid warming in the northern hemisphere. Boreal summer insolation modulated key changes in climate and atmospheric circulation during the early Holocene, with a progressively increasing influence from anthropogenic effects related to deforestation, agriculture and industrialisation^1^. On decadal-to-centennial timescales, these low-amplitude trends were also punctuated by a series of repeated, aperiodic, century-scale cold ‘snaps’ characterised by pronounced surface cooling, an altered hydrological cycle and ecological disruption^2,3^. First identified in the North Atlantic as distinct peaks in Icelandic volcanic glass and/or haematite-stained grains, these cold events were initially linked to southward advection of icebergs from the Nordic and Labrador Seas, and thus to major perturbations in high-latitude ocean dynamics^4^. These peaks were later found to align with discrete cooling anomalies recorded in independent compilations of more than 45 globally distributed terrestrial records^3,5^, as well as nine distinct sea-surface temperature anomalies in marine sediment cores off the coast of West Africa^6^. It is now clear that these events were not confined to the North Atlantic and that their effects propagated at least as far south as the tropics. Understanding what caused these unusually cold intervals is critically important for three reasons: (i) to determine if they were externally forced versus internally generated; (ii) to determine whether they represent an instantaneous (threshold) or cumulative (delayed) response to forcing under near-modern boundary conditions^7^ and (iii) to enhance our ability to model the system’s response to both natural and anthropogenic forcing under a range of future emission scenarios^8^.

Explanations for these cold events have included solar forcing, ocean circulation changes and atmospheric re-organisation^2^. Alongside these ideas, the importance of explosive volcanism as a driver of pre-industrial climate change has continued to gain interest^9–11^. Volcanological evidence for an eruption is often difficult to date precisely, leading to difficulties in assessing the impact of volcanic eruptions on Holocene climate change and variability. A key development was the generation of ice-core sulphate-based reconstructions of volcanism across the Holocene^9^, which, combined with tree-ring and historical records, now reveal compelling temporal coincidences between annual-to-decadal-scale temperature variations, major explosive eruptions, and key cultural transitions, upheavals and hardships^11^. By proposing a causal link between volcanic eruptions and climate disturbance, these coincidences highlight a clear need for a deeper investigation into how long volcanic-induced climate impacts can last, and the utility of paleoclimate data and historical eruption records for this purpose.

The amount of sulphur injected by an eruption into the stratosphere largely controls the short-term climate response. Volcanic sulphur oxidation in the atmosphere forms sulphate aerosols, which scatter incoming solar shortwave radiation, thereby reducing the amount of solar energy reaching the Earth’s surface and producing rapid cooling^12^. This impact can arise either from a single, large, sulphur-rich eruption^13,14^ or from a cluster of eruptions occurring across short time intervals^15–17^. Less well understood are stratospheric volcanic sulphate aerosol-induced dynamical changes within the climate system^18^. A growing body of research shows that eruptions resulting in a hemispherically asymmetric sulphate aerosol burden create an energy imbalance; the disproportionately strong aerosol-induced cooling in the hemisphere of the eruption shifts the intertropical convergence zone (ITCZ) toward the relatively warmer hemisphere^19^, leading to major reorganisations of atmospheric circulation and precipitation patterns across both hemispheres^19–23^.

An eruption’s climate impact depends on eruption magnitude, the amount of stratospheric sulphur injected, latitude, background conditions^24–26^, and numerous other poorly-constrained variables. Nonetheless, compilations of explosive volcanism combined with refinements in paleoclimate time-series from diverse climate proxies reveal that volcanism has repeatedly triggered annual-to-decadal scale cooling episodes throughout the Common Era^10,16,27^. Building on these observations, several studies suggest that feedbacks within the coupled atmosphere–ocean system could prolong the climate effects of explosive eruption(s) beyond the immediate aerosol phase, and these feedbacks may contribute to centennial-scale cooling events observed throughout the Holocene^15,16,19,27,28^. However, very few large (magnitude (*M*) ≥ 6) eruptions occurred across the historical era^29^, so the precise nature of the feedback is still poorly understood.

Assessing the timing, frequency, magnitude and climate-relevant gas emissions of the largest Holocene eruptions rests heavily on the availability of palaeovolcanic records. These records fall into two categories: (1) volcanological databases storing information on discrete eruption events^30^ and (2) proxy archives in which eruptions are recorded as geochemical and/or stratigraphic signals^31^. Ice cores are the most common archive used for this purpose, particularly those from Greenland and Antarctica, where eruptions are recorded as sulphate concentration peaks^9,32^ or as crypto-tephra deposits^33,34^. Despite their usefulness, both sources of Holocene eruption information have their limitations. For example, an eruption’s inclusion in a volcanological database requires the identification of a source volcano, and the number of undocumented eruptions increases with age^35^. Conversely, ice cores retain a sulphate signature from both documented and undocumented volcanic eruptions, but do not provide unambiguous evidence regarding the source volcano, limiting the information regarding eruption parameters that determine the climate response^31,36^. Dating individual eruptions is similarly challenging; whereas the prehistoric volcanological record relies largely on radiometric dating techniques, the ice core sulphate records preserve accurate dates for eruptions, though often without additional context, including the source volcano.

Systematic integration of geological and chemostratigraphic data has already shown great potential for exploration of long-term volcano-climate interactions during the Common Era^9^. However, no study has yet extended this approach across the entire Holocene. Here, we present an extensive data compilation that provides a coherent picture of volcanism and abrupt climate change spanning the past ~12,000 years: presented herein as kiloyears (ka) before 1950 CE. By synthesising data from terrestrial eruption deposits, ice cores, paleoclimate archives and glacial reconstructions, we evaluate the temporal match between major eruptions and abrupt cooling events. We then use these results to assess if a robust statistical link between explosive volcanism and centennial-scale cooling events across the NH exists.

## Results

**Fig. 1:**
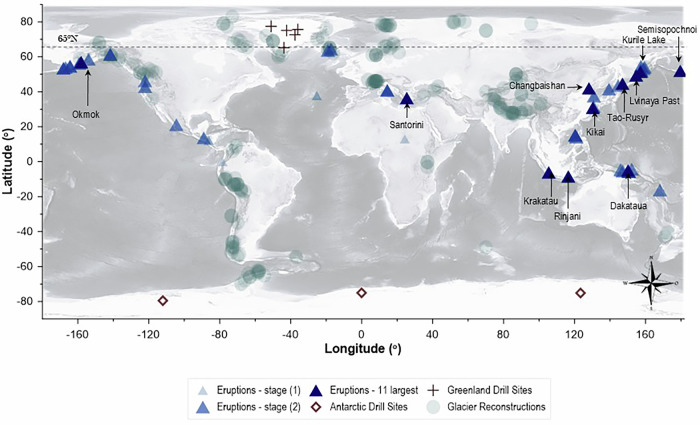
Map displaying the locations of eruption events, glacial reconstructions, and key paleoclimate records. Locations of eruption events are given as blue triangles, and reconstructed glacier advances as (grey circles)^38^. Also featured are paleoclimate reconstructions from Greenland (dark brown crosses), the North Atlantic (orange crosses) and Antarctica (brown diamonds) relevant to assessment of Holocene climate change^31,81,112^. The sources of the eleven largest (*M* ≥ 7) eruptions are labelled and coloured darker blue. A dashed black line marks 65°N latitude. Data points are overlayed onto an adapted (greyscale) AWI Basemap (v. 2025). Basemap data from the Alfred Wegener Institute AWI Basemap (v. 2025), available at 10.1594/PANGAEA.988305, licensed under CC BY 4.0 (https://creativecommons.org/licenses/by/4.0/).

To assess the correlation between explosive volcanism and abrupt cooling events, we first compiled information on the largest known Holocene volcanic eruptions north of 20°S by volume (*n* = 51; Table S1)^37^, and major glacial advances (*n* = 22)^38^ (Fig. 1). The timing of each glacial advance is determined from the compilation of radiometric ages acquired from moraines across 17 regions compiled by Solomina et al.^38^. By marking the point of moraine stabilisation, these ages record the culmination of sustained positive glacier mass balance, and therefore provide a near-direct indication of centennial-scale temperature and precipitation fluctuations (see “Methods”)^39,40^. Sustained intervals in which accumulation exceeds ablation lead to glacier thickening and increased ice flux to the terminus, producing advances after a dynamic response lag of years to decades^41^. For example, periods of accelerated glacier growth typically occur when conditions are cold enough to sustain ice throughout the year^42^. Therefore, alpine glaciers are much more sensitive to centennial-scale climate shifts than large ice sheets, which more clearly capture millennial-scale glacial-interglacial oscillations^43^.

In periods and in regions where annually resolved glacier records are abundant, such as in the Alps, their advances are predominantly attributed to the climate effects (and feedbacks) from clusters of major volcanic eruptions with time delays as low as 1 year, to several decades^17,40,44^. However, over the full Holocene and on a global scale, we cannot quantify the precise time offset between moraine formation, and the initial ‘trigger’ forcing because: (i) our dataset is a regional compilation of terrestrial exposure ages rather than annually resolved glacier records and (ii) the associated moraine ages are almost all synthesised from multiple glaciers of different sizes, shapes and types and located at various elevations^38^. Here, we test the time-association between the largest (*M* ≥ 7) known Holocene volcanic eruptions north of 20°S by volume (*n* = 11; Table S1) and glacial advance datasets relative to the null hypothesis: if explosive volcanism is not causally linked to Holocene glacial advances, eruptions dated to that period would not coincide with major cooling events more often than they would by chance alone. This negates the need for a precise ‘match’ between the eruption and moraine exposure ages, and so allows us to account for non-linear processes that could prolong the effects of the initial volcanic cooling. This approach also avoids issues of circularity that could arise if we were to test the timing of volcanic eruptions relative to cooling event signals in the ice cores, given that older sections of existing ice core records are chronologically constrained using volcanic tephra layers as time-stratigraphic markers^45^.

We first captured the lag time between a given glacial advance and the nearest eruption date, and then calculated a root-mean-square (RMS) statistic from all event pairs, where the RMS statistic is a function of ${N}_{{\rm{e}}}$ (the number of eruptions), ${{\mathrm{T}}}_{{\mathrm{k}}}^{{\mathrm{e}}}$ (the eruption dates) and ${{\mathrm{T}}}_{\mathrm{j}}^{\mathrm{c}}$ (the glacial advance dates). The probability distribution and cumulative distribution functions of this statistic were then assessed by generating 10 million random sets of eruption dates (${E}_{{\rm{R}}}$). Next, the probability that the RMS best match statistic for the distribution of the actual ages (${E}_{{\rm{A}}}$) was significantly less than for ${E}_{{\rm{R}}}$ was determined. ${E}_{{\rm{A}}}$ was defined by dates all calibrated to the IntCal20 curve^46^. Our analysis shows that all known exceptionally large (*M* ≥ 7) Holocene eruptions are within chronological uncertainty of a glacial advance, and that for 72% of these eruptions, evidence for the corresponding glacial advance exists in both northern and southern hemispheres. This relationship is significant at the 99% confidence level (*p* = 0.002), and statistical significance is also maintained if all *M* > 6.7 eruptions (*n* = 14) were included in the analysis (*p* = 0.006).

**Fig. 2:**
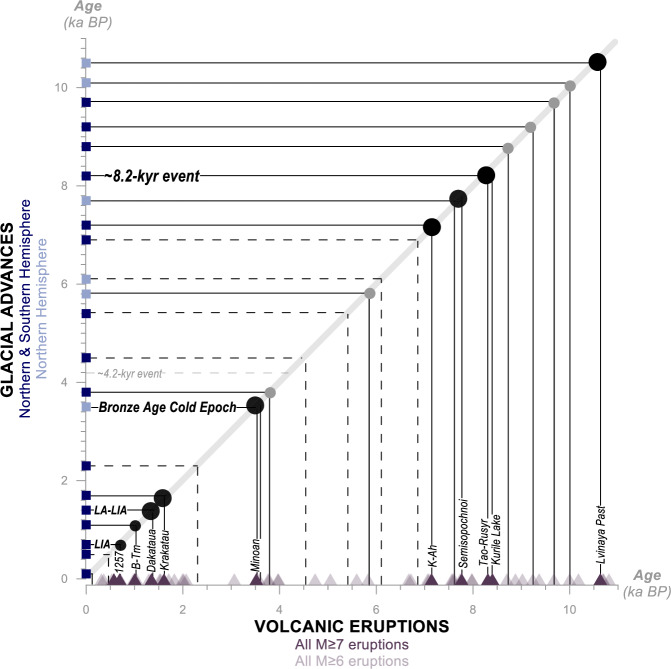
Temporal links between the onset of glacial advances^38^ and known volcanic eruptions occurred during the Holocene. Large black circles mark coincidences (within chronological uncertainty) between glacial advances and the largest eruptions in our compilation (*M* ≥ 7), and smaller grey circles represent coincidences with any of the other 31 eruptions that passed the filtering (described in “Methods”). Dashed lines represent glacial advances that do not occur within the uncertainty of a known volcanic eruption meeting our predefined selection criteria. The largest eruptions are labelled by their corresponding unit name. Chronological uncertainties associated with each eruption and glacial advance in this figure are included in a Supplementary Information File.

**Fig. 3:**
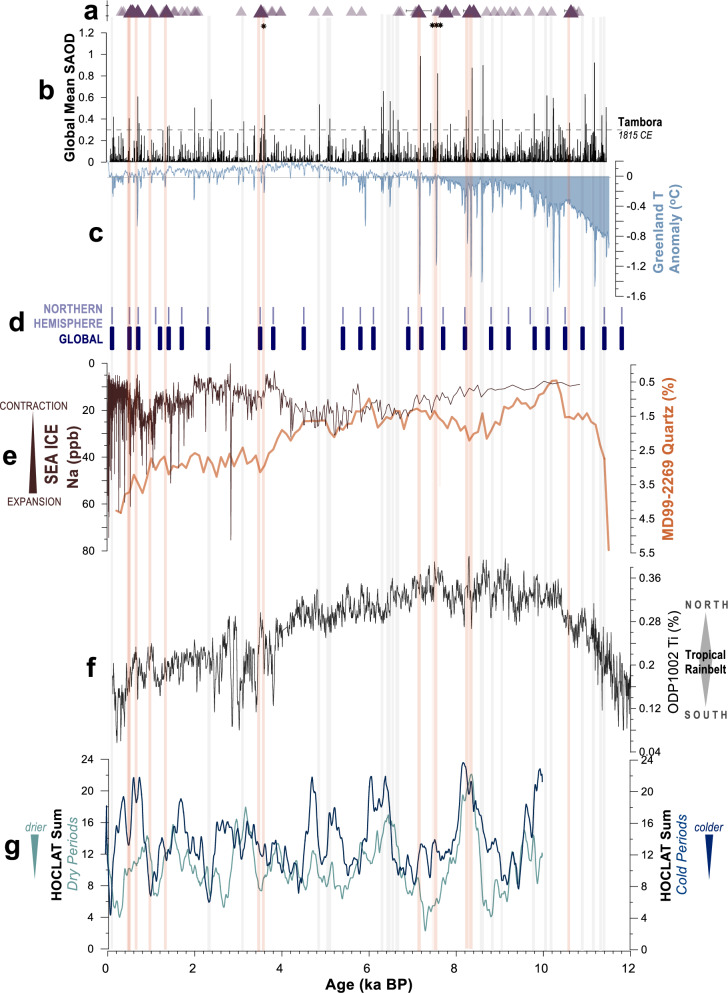
A comparison of key datasets mentioned within the text. **a** Explosive volcanic eruptions compiled as part of this study (see “Methods”); **b** ice-core derived estimates of stratospheric aerosol optical depth (SAOD) from the HolVol reconstruction, presented on the WD2014 timescale and with a dashed horizontal line marking estimated SAOD values produced by Tambora (1815 CE)^9,31^; **c** reconstructed Greenland temperature anomalies over the Holocene derived from argon and nitrogen isotopes^10^; **d** onset dates for major northern hemisphere (dark blue) and southern hemisphere (light blue) glacial advances^38^; **e** records of sea ice variability derived from the RECAP ice core (Greenland)^64^, and sediment succession MD99-2269 (offshore Iceland)^113^; **f** a record of elemental titanium (Ti) in the ODP 1002 sediment core (Cariaco Basin, Venezuela) smoothed by a three-point running mean, where higher Ti concentrations indicate more terrestrial runoff as evidence for a farther-northward summer ITCZ position^47^; **g** weighted curves representing the sum of cold and dry periods within the HOCLAT paleoclimate data compilation^3^. Key eruption events are labelled, and asterisks (*) mark events for which tephra deposits have been identified in Greenland^34^. Orange bars mark the largest 10 eruptions in our compilation that occur <100 years prior to a glacier advance event. Grey bars mark large SAOD peaks with no known eruption source, but also occur <100 years prior to a glacier advance event.

Our statistical analysis provides a clear indication that explosive volcanism may be a candidate trigger for Holocene cold events and shows that exceptionally large (*M* > 7) eruptions coincide with abrupt Holocene cooling events more closely than would be expected by chance. The link between major eruptions and abrupt cooling events is further supported if we extend our eruption record to include all known Holocene *M* ≥ 6 events north of 20°S (Fig. 2), and assess these events relative to records of atmospheric sulphate loading^31^, temperature in Greenland^10^, major glacial advances^38^, mid-latitude temperature and aridity fluctuations^3^ and tropical rain belt dynamics^47^ (Fig. 3). Three key findings emerge from this analysis. First, only four out of 22 glacial advances do not occur within the uncertainty of a known volcanic eruption (Fig. 2). Second, our recalibrated IntCal20 eruption ages (Table S1) show good agreement with ice-core derived ages; an agreement underscored by events whose tephra deposits have been identified in the Greenland ice cores (e.g., Aniakchak II, Mazama, Okmok II and Mai-f (7.58 ± 0.09)) (Fig. 3)^34,48^. Third, over two-thirds of the largest (M ≥ 7) eruptions in our compilation occur within uncertainty of a peak in stratospheric aerosol optical depth (SAOD) exceeding the 1815 CE Tambora eruption (>30 teragrams (Tg) S^36^). Given the 1815 CE Tambora eruption’s considerable climate impact^49^, this implies that almost all of the largest eruptions within our compilation would have had a similar, if not greater, climate impact. This is especially relevant when considering eruptions such as 1257 CE (Samalas: 0.69 ± 0.001 ka), K-Ah (7.14 ± 0.29 ka: IntCal20), or the caldera-forming eruption of Kurile Lake (8.4 ± 0.022 ka: IntCal20), which all released more than twice as much sulphur into the atmosphere as Tambora ( > 75 Tg S) (Fig. 3).

Five exceptionally large (>70 Tg S) VSSI and SAOD (>0.5) peaks exist that are not assigned to a documented eruption (Fig. 3). This might reflect that the eruption responsible for the sulphate spike is not yet documented, possibly due to erosive or volcanic modification of eruption deposits^50^. They may also represent smaller (*M* < 6), unusually sulphur-rich eruptions. For example, the 1963 CE eruption of Mount Agung (Indonesia) and the 1982 CE eruption of El Chichón (Mexico) are both *M* < 5 (<1 km^3^ ejecta) eruptions associated with high SO_2_ emissions, and have been linked to measurable climate disturbances^51,52^. Temporal coincidence alone is not enough to definitively ascribe a geochemical signal to a source eruption, and it is only the geochemistry of tephra particles captured in the ice that allows for unambiguous correlation of a sulphur signal to a specific eruption^34^. Therefore, these SAOD peaks may have been produced by a small (*M* < 6), SO_2_-rich, and/or high-latitude eruption (Fig. 3), but it is also possible that currently published eruption dates exhibit uncertainties too large to make a definitive ice core match.

Our dataset also highlights instances where known *M* ≥ 6 eruptions do not coincide with comparably large SAOD peaks or a glacial advance (Fig. 2). This discrepancy may relate to variables such as bulk composition, degassing rates and viscosity, which control the relationship between eruption magnitude and sulphur released^12^. For example, the Millennium/B-Tm eruption of Changbaishan volcano (China/North Korea) was among the largest eruptions of the Common Era, but excessive sulphur degassing prior to eruption limited the amount of sulphur released during the eruptive event itself, potentially explaining the absence of a pronounced SAOD peak associated with the eruption^53,54^ (Fig. 3b). Syn-eruption sulphur removal from the plume by hydrothermal fluids and/or lithic materials could also prevent sulphur deposition in polar snow^55,56^. Other processes are also relevant. For example, the interaction of water with erupted magmas can ‘scrub’ sulphur from the plumes prior to atmospheric injection^57^, and may explain the notable absence of a large SAOD peak corresponding to the Minoan eruption of the Santorini volcano, Greece (Fig. 3). Both the Millennium/B-Tm and Minoan eruptions underscore how magnitude and the amount of sulphur released to the atmosphere during an eruption are not always correlated. Different high-magnitude eruptions can release varying amounts of sulphur into the atmosphere, leading to substantially different impacts on the climate^2^.

## Discussion

Our synthesis reveals that volcanic forcing has likely played a far greater, and more persistent, role in shaping Holocene climate variability than previously recognised. By compiling records of explosive volcanism, atmospheric sulphate loading, glacial dynamics and climate variability spanning the past 12,000 years, we find that over 80% of Holocene glacial advances occurred within chronological uncertainty of a large (*M* ≥ 6) northern hemisphere eruption, with the relationship between glacial advance and the largest (*M* ≥ 7) eruptions significant at the 99% confidence level. Combined with evidence for rapid (<10–20-year) sea ice and glacial expansion followed by prolonged ocean circulation weakening, and interhemispheric climate reorganisation following major eruptions (Fig. 3)^15–17,27,28,58^, these findings suggest that the climatic consequences of volcanic forcing can persist for centuries—long after the dissipation of sulphate aerosols. If these dynamical, long-term sulphate aerosol effects are considered relative to the role of long-term, low amplitude climate forcing factors (e.g. variations in insolation^2^), explosive volcanism may represent the driver that prompted the climate to cool rapidly, and recurrently, at discrete points throughout the Holocene. Therefore, our work underscores the need to view volcanic perturbations not merely as short-lived radiative events, but as important catalysts—capable of initiating complex feedbacks within the coupled ocean–atmosphere system^59^.

**Fig. 4:**
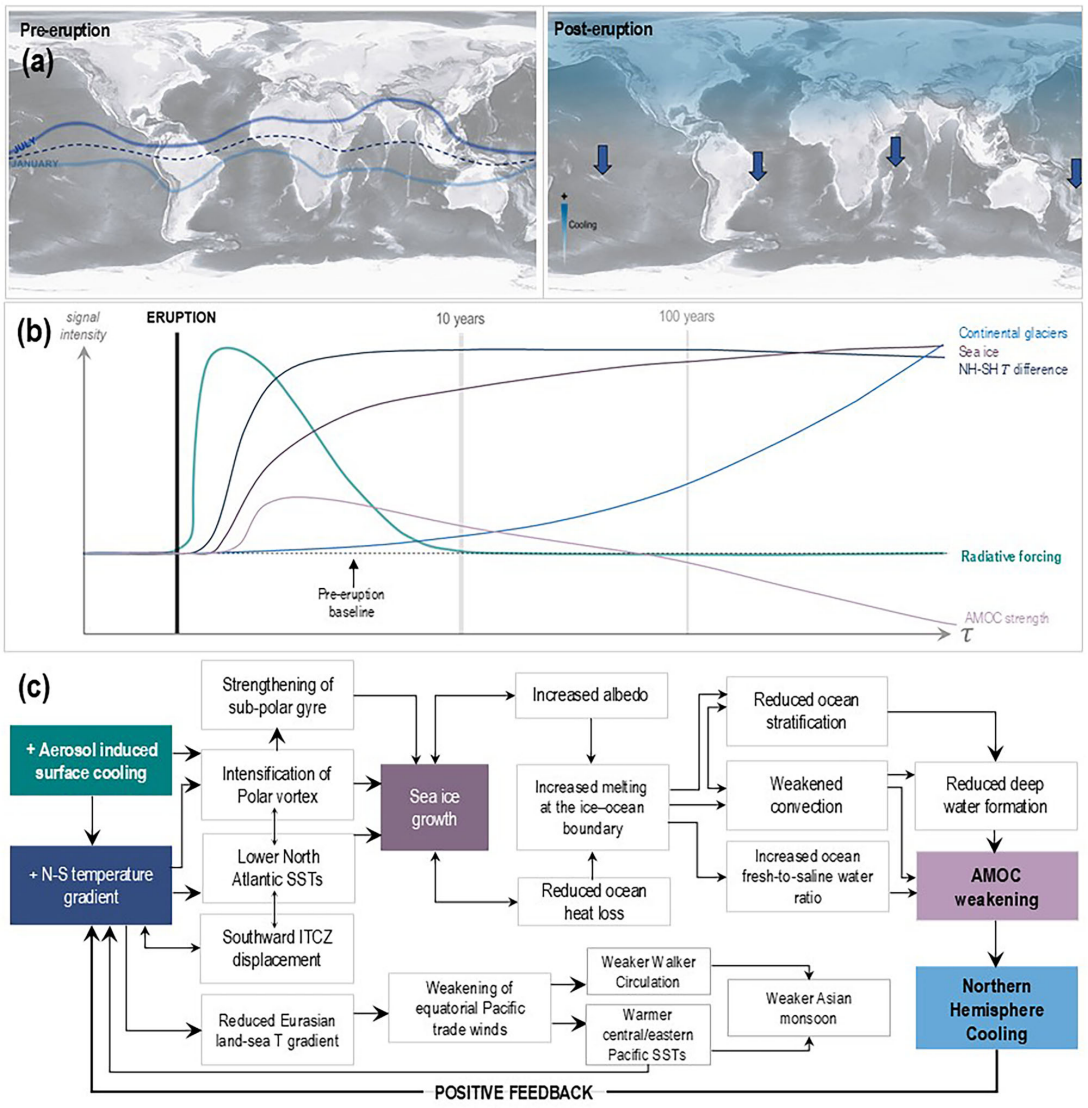
A framework illustrating how a large (*M* ≥ 6) northern hemisphere volcanic eruption could produce a centennial-scale glacier advance. **a** World maps illustrating changes in the hemispheric temperature gradient following an explosive (NH) eruption, with blue shading marking the strength of cooling (darker = colder). For the pre-eruption map, longitudinal variations in mean ITCZ position based on average precipitation for January (grey) and June (blue) between 1979 and 1995 are also shown, with a dashed black line denoting the mean position (data sourced from the International Research for Climate Prediction (http://iri.ldeo.columbia.edu); adapted from Deplazes et al.^114^). Data points are overlayed onto an adapted (greyscale) AWI Basemap (v. 2025). Basemap data from the Alfred Wegener Institute AWI Basemap (v. 2025), available at 10.1594/PANGAEA.988305, licensed under CC BY 4.0 (https://creativecommons.org/licenses/by/4.0/). **b** Schematic diagram showing the time ($\tau$)- resolved evolution of climate system components known to be sensitive to volcanic forcing, and/or propagate eruption-induced feedbacks for multiple centuries. Curves are colour-coded to match their corresponding box in part (**c**), and a dashed grey horizontal line marks the pre-eruption baseline state for each variable; such that if a curve goes above (below) this line, the associated signal is stronger (weaker) than it was under pre-eruption conditions. **c** A unified framework describing the sequence of climate feedbacks proposed as drivers of Holocene glacial advances. Acronyms used include: SSTs sea-surface temperatures, AMOC Atlantic meridional overturning circulation, ITCZ intertropical convergence zone.

Chronological uncertainties, incomplete eruption records, and the lack of a clear mechanistic framework describing this relationship have complicated testing this hypothesis. Here, we propose a framework describing how volcanic eruptions induce positive feedbacks that could ultimately cause a Holocene cold event (Fig. 4). The radiative effects arising from a sulphur injection into the atmosphere by a large explosive eruption(s) in the northern hemisphere would first cause pronounced surface cooling^18^. Such a cooling effect also arises if we consider the magnitude-frequency relationship^60^, which highlights that high-frequency ‘clusters’ of smaller explosive, and/or particularly sulphur-rich effusive eruptions may also deliver substantial quantities of sulphur to the lower stratosphere, capable of producing a potent radiative effect^15,16^.

The initial, sulphur-induced cooling response would trigger two coupled responses in the climate system. The first involves the creation of a pronounced interhemispheric energy imbalance across the atmospheric energy flux equator^20^, decreasing northward heat transport and displacing the ITCZ southward (Fig. 4a)^19,61^. This would expand the northern polar cell, shift the jet stream southward, and reduce temperature and precipitation across the northern hemisphere—responses strongly supported by both proxy records^21,62,63^ and model simulations^22,23,61^. The second involves a rapid expansion of sea ice in the North Atlantic, in response to the volcanic-aerosol-driven surface cooling (Fig. 4b). Several studies have shown that both alpine glacier^44,58^ and sea ice^27,28,51^ extent are both highly sensitive to volcanic forcing, but also that the extent and characteristics of sea ice in the North Atlantic region underwent a series of abrupt changes throughout the Holocene (Fig. 3e)^64,65^. Sea ice reflects incoming solar radiation, while also serving as a physical barrier that inhibits the exchange of heat and moisture between the ocean and atmosphere^51^. Thus, expansion of sea ice extent alters atmosphere-ocean heat exchanges, stratification, and deep-water formation sites in the Arctic and Nordic Seas^66,67^. Sea ice expansion typically occurs within a decade of an eruption in response to reduced SSTs, strengthening the subpolar gyre and the polar vortex (Fig. 4c)^68^, and causing an initial AMOC intensification^15,61^.

On centennial timescales, two processes amplify the role of sea ice dynamics (and their associated feedbacks)—relative to the initial radiative response. The first is increased surface albedo due to more extensive sea ice coverage, which sustains the colder conditions conducive to further sea ice growth^67^. The second is reduced oceanic heat loss beneath the ice, creating a subsurface heat anomaly that enhances melting, freshens the surface ocean and weakens deep convection^28^. This heat anomaly also reverses the initial strengthening of the subpolar gyre, causing a spin-down effect that progressively reduces ocean stratification and advection of heat and salt^28^. Not only does this further amplify the temperature and salinity gradient, but it also prompts the poleward advection of anomalously cold and fresh subpolar water into the Arctic Ocean, resulting in decreased heat transport, reduced ice–ocean heat exchange, decreased rates of sea-ice melt, weakening of the AMOC, and muted deep water formation^28,61^. Superimposed on these sea-ice-driven responses are atmospheric adjustments associated with the southward ITCZ displacement (Fig. 4c). Alteration of key ocean–atmosphere feedbacks would not only result in a weakened Walker circulation^69^, but also a tendency toward warmer central/eastern equatorial Pacific SSTs characteristic of El Niño conditions^70^. By simultaneously weakening monsoon circulations^71,72^ and enhancing precipitation in parts of the southern hemisphere^21,23^, these tropical teleconnections reinforce the atmospheric reorganisation triggered by the initial aerosol forcing^22^. Hence, they represent the culmination of the ocean–atmosphere feedbacks initiated by volcanic aerosol forcing, and superimposed onto the background state set by lower-amplitude forcing factors (e.g., insolation changes)^2^. In this framework, volcanism acts as a pacing and intensifying mechanism, triggering nonlinear responses within the climate system that emerge from the interaction between episodic sulphur emission events and other underlying, more gradual radiative fluctuations.

### The 8.2 ka event

**Fig. 5:**
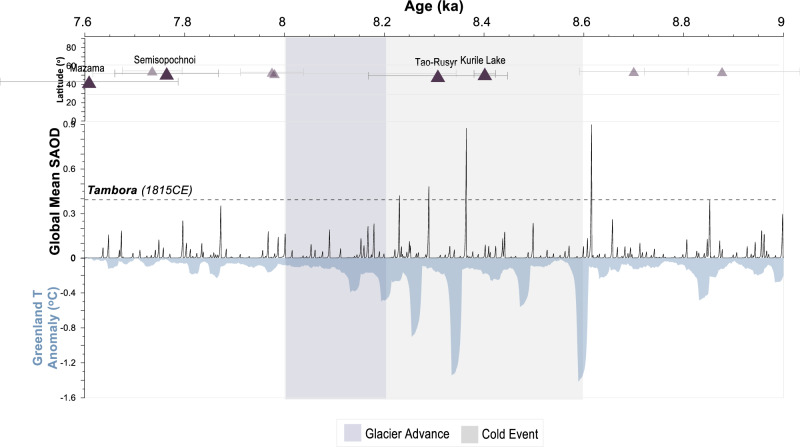
Comparison of volcanic and chemostratigraphic records spanning the 8.2 ka event interval. Triangles denote the timing of the large explosive eruptions included in our compilation (Table S1), with dark colouring given to those large enough for inclusion in our statistical analysis, and grey horizontal lines displaying uncertainties associated with the IntCal20 calibration. Blue shading shows reconstructed Greenland temperature anomalies over the Holocene derived from argon and nitrogen isotopes^10^, compared with a record of global mean stratospheric aerosol optical depth (SAOD) generated from the HolVol dataset^31^. Vertical purple shading marks an interval marked by evidence for glacial advance^38^, and grey shading marks the timing of a discrete cold event within the HOCLAT proxy data compilation^3^.

**Fig. 6:**
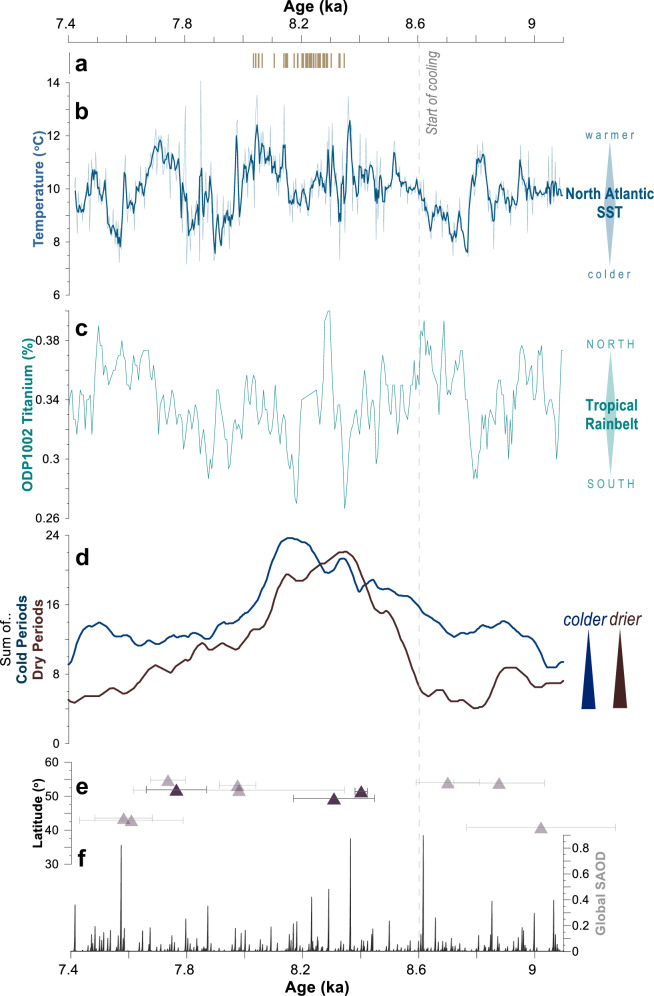
Comparison of paleoclimate and volcanic records spanning the 8.2 ka event interval. **a** Stable oxygen isotope (δ^18^O) anomalies obtained by breakpoint analysis of global speleothem records, where all anomalies are larger than speleothem record measurement uncertainties^115^. **b** Alkenone-derived sea surface temperature (SST) reconstruction for the western Nordic Seas^116^. **c** Elemental titanium (Ti) in the ODP 1002 sediment core (Cariaco Basin, Venezuela) smoothed by a three-point running mean, where higher Ti concentrations are interpreted as evidence for a farther-northward summer ITCZ position^47^. **d** Weighted curves representing the sum of cold and dry periods within the HOCLAT paleoclimate data compilation^3^. **e** Explosive volcanic eruptions compiled as part of this study, with *M* ≥ 7 events coloured in darker purple (see “Methods”). **f** Global mean stratospheric aerosol optical depth (SAOD) generated from the HolVol dataset^31^, which shows clear evidence for a clustering of 3 ‘Tambora-sized’ eruptions preceding the 8.2 ka event.

The 8.2-kyr event offers a compelling illustration of how such mechanisms may have operated (Figs. 5 and 6). Traditionally, a catastrophic meltwater flood into the North Atlantic from the Laurentide Ice Sheet was thought to have caused the 8.2 ka event^73–76^. However, recent model simulations have cast doubt over outburst flooding as the sole cause of the event, as none have yet succeeded in reproducing cooling with sufficient amplitude to be sustained for multiple centuries^77–79^. Therefore, although perturbation to North Atlantic Ocean circulation is well established as a key driver of the 8.2 ka event’s severity, the idea that this event was caused solely by freshwater forcing remains contentious^80^.

Our compilation shows that two of the largest known high-latitude eruptions of the Holocene—Kurile Lake (8.4 ± 0.02 ka; M7.3), and Tao-Rusyr (8.31 ± 0.14 ka; M7.0)—occurred at the onset of the peak 8.2-kyr cooling (Fig. 5). Two anomalously large volcanic sulphate and acidity spikes within several Greenland ice cores^31,81^ are also nearly contemporaneous with these two eruptions, and existing melt inclusion and matrix glass measurements suggest that both were likely to have produced sulphur emissions comparable to the 1815 CE eruption of Tambora^82^. However, also visible is a third large volcanic sulphate peak with an unknown source at ~8.6 ka (Fig. 5). Although no eruptions in our compilation clearly align with this peak, one possibility is that it is linked to the effusive Þjórsá eruption of the Bárðarbunga volcanic system in southern Iceland at ~8.6 ka—the largest Holocene lava flow known to have been emplaced during a single event^83,84^.

A growing number of paleoclimate records covering this interval show that the earliest signs of cooling associated with the 8.2 ka event began between ~8.6 and 8.5 ka^85^, over 100 years before the date of the earliest known Laurentide outburst flood (8.47 ± 0.3 ka)^74^. Indeed, the peak cooling signal in the Greenland temperature reconstructions occurs at ~8.6 ka—immediately following an unusually large injection of sulphur into the atmosphere (Fig. 5). During the early Holocene, freshwater inputs to the North Atlantic would already be high due to the opening of a new drainage pathway into the Hudson Strait, and accelerated melt of the remnant Laurentide Ice Sheet (LIS) during the final stages of deglaciation^86–88^. Furthermore, numerous marine and terrestrial proxy records also suggest gradually increasing temperatures across the northern hemisphere during this time, associated with major readjustment of regional temperature gradients, progressive strengthening of the Atlantic Meridional Overturning Circulation (AMOC)^80^, and progressive shifts in solar activity^80,89^. Together, these records strongly suggest that an intermediate climate state existed prior to the 8.2-ka event characterised by moderate-to-low sea-ice extent^64^, high variability in solar output^89^, declining insolation^80^, elevated meltwater inputs to the North Atlantic^86^, and large-scale atmospheric reorganisation^90^. Hence, a climate system that would have been highly sensitive to external forcing relative to the mid- and late-Holocene^64,91^.

The Kurile Lake, Tao-Rusyr and unknown ~8.62 ka eruptions are all likely to have injected massive quantities of sulphur into the stratosphere, consistent with ice core records of volcanism and regional cooling from Greenland (Fig. 5). We propose that their sulphur emissions perturbed a climate system already primed for change by solar, cryospheric and atmospheric factors, by promoting hemispheric cooling that reinforced ocean–atmosphere feedbacks already operating near a stability threshold. For example, sulphur emissions produced by the first eruption at ~8.62 ka could have caused a transient expansion of sea ice in response to the surface cooling, prompting a decrease in surface heat loss (Fig. 4c). Alongside this surface cooling would also be southward displacement of the tropical rain belt, which would propagate cooling and/or aridity in the northern hemisphere^90,92–94^, and warming and/or increased moisture in the southern hemisphere^95,96^. Although initially it seems counterintuitive to suggest warming of the southern hemisphere in conjunction with glacial advance (Fig. 3), glacier growth also requires a state of positive mass balance^42^. In this case, warmer and wetter conditions linked to a southward ITCZ shift have increased orographic snowfall in the high Andes^97^, which would promote glacier advance despite regional warming^38^. Less than 200 years later, the stratospheric sulphur injections produced by the Kurile Lake and Tao-Rusyr would further cool the climate before it could return to its pre-forced state, amplifying the already-present sea ice feedbacks^27,28,98^, and culminating in a pronounced cooling/drying signal centred on ~8.2 ka (Figs. 5 and 6). Therefore, the occurrence of an ~8.6 ka precursor explosive, sulphur-rich eruption could reconcile existing proxy-model discrepancies that cast uncertainty over whether, and how, early cooling signals relate to the peak ~8.2 ka event^85^.

Considering this evidence, we propose that the 8.2-kyr event cooling reflects the ongoing delivery of freshwater to the North Atlantic from the deteriorating LIS^86^ combined with cooling associated with several closely spaced, very large and very high latitude eruptions (Figs. 3e and 5). This hypothesis is consistent with the event’s distinct ‘double-plunging’ structure^90,93,99^, which suggests two distinct cooling phases separated by a brief and partial recovery. It is also consistent with a scenario where two large sulphur injections to the atmosphere were spaced closely enough for the climate forcing potential of the second eruption to be superimposed onto that of the first—effectively amplifying the disturbance^16^. Hence, our synthesis supports a dual-forcing scenario in which volcanic activity, rather than meltwater release alone, provided the necessary radiative trigger for the 8.2 ka event’s amplitude and persistence.

In conclusion, our results highlight the importance of considering volcanic forcing as an integral driver of Holocene climate variability. Large explosive eruptions were not isolated, transient perturbations, but rather recurrent catalysts that activated feedbacks within the coupled climate system—particularly through sea ice–AMOC interactions and interhemispheric energy imbalances. When sustained over centuries or repeated through clusters of eruptions, interacting feedbacks can reorganise large-scale ocean–atmosphere dynamics, leading to persistent cooling, hydrological shifts and glacier advance across the northern hemisphere. This sequence provides a physically consistent mechanism linking episodic volcanism to centennial-scale Holocene cold events, consistent with the temporal correlation identified in our statistical analysis. Although uncertainties in eruption dating and regional glacier responses remain, the weight of evidence very strongly suggests that volcanic perturbations can trigger self-sustaining cryospheric–oceanic feedbacks that extend well beyond aerosol lifetimes. Recognising these dynamical linkages not only advances our understanding of past climate variability but also offers a framework for anticipating the potential long-term impacts of future large eruptions in a warming world.

## Methods

### Data acquisition

#### Cold events

We define the timing of ‘abrupt cold events’ by the onset of major glacial advances^38^. Glacier fluctuations provide a near-direct indication of temperature and precipitation variability over longer (>100-year) timescales^39,40^, with periods of accelerated glacier growth typically occurring when conditions are cold enough to sustain ice throughout much of the year, and prompt precipitation to fall predominantly as snow^42^. Variations in glacier extent can also be constrained in space, which is particularly important here given that cooling effects following high- and mid-latitude eruptions are likely to be spatially heterogeneous. Therefore, given that the cold intervals of the Holocene were nearly all punctuated by the advance of temperate glaciers^38^, these glacial variations documented by sedimentological and geomorphic evidence can account for the non-linearities that may emerge when considering longer-term volcanic climate perturbations, and so provide effective qualitative indicators of regional and global cooling^100^ that are less affected by short-lived (<10-year) variations relative to high resolution records such as lake sediments, tree rings and ice cores^3^.

#### Volcanic eruptions

Geological data were compiled in two stages. The first stage involved the extraction of eruption data from the large magnitude explosive eruption database (LaMEVE), which contains data for 1883 explosive eruptions produced by 471 discrete volcanoes during the Quaternary (~2580 ka to present^37^). Eruptions were immediately removed if they did not correspond to our pre-defined criteria for location (90°N to 20°S) and age (~11.7–0.6 ka). This latitude band also accounts for tropical (0–20°S) southern hemisphere eruptions, which could have also distinctly influenced the climate of the NH^9,31^. For example, evidence has been found for both climate extremes and Alpine glacier advances during the 17th and 19th centuries following eruptions of Huaynaputina (1600 AD, 17°S) and Tambora (1815 AD, 8°S)^17,40^.

A conservative magnitude filter of *M* ≥ 5.9 was initially applied to our search, where *M* is quantified to one decimal point. Thus, eruptions listed as *M* = 5.9 include all eruptions with *M* values between 5.9 and 6.0. Eruptions with magnitudes listed as 5.9 were independently assessed in order to verify volumetric estimations and their associated magnitude calculations. For example, whether primary ash fall occurred over the ocean^101^, or whether the eruption resulted in the formation of a caldera structure^102^. Two M5.9 eruptions were retained within our compilation in light of this assessment: the Jala Pumice eruption of Ceboruco and the KS2 eruption of Ksudach. The decision to filter eruptions by magnitude was made because this parameter provides a practical, consistent and reproducible proxy for eruption scale^103^, and subsequently climate forcing potential (Fig. S3, Text S3).

The second stage of data compilation involved a thorough intra-study comparison, where volumetric and chronological data were cross-checked with all literature currently available for each eruption individually, to determine the consistency in values. Where possible, magnitude estimates were also cross-checked for each eruption across different modes of data acquisition (e.g., geological, geophysical and model-based), to ensure that the values provided in the literature were consistent across different methods of estimation. Following stages one and two, 51 eruptions matched our selection criteria. Eight eruptions were removed during the second compilation stage, due to inconsistencies in magnitude and/or age estimates when values were crosschecked, and two of these eight events also lacked high-quality age determinations.

From the 51 eruptions that remained in our compilation following stages (1) and (2), ages for 45 are derived from ^14^C (radiocarbon) dating. To ensure chronological consistency across our dataset, all eruption ages were first recalibrated using the IntCal20 curve^46^, and modelled within the OxCal online version 4.4 model (https://c14.arch.ox.ac.uk/oxcal.html). To assess time-resolved changes in age precision as a function of refinements in radiocarbon age determinations, all ^14^C eruption ages were also calibrated to previous iterations of the radiocarbon curve, each named based on the year of publication: IntCal13, IntCal09 and IntCal04. Ages ascribed to the majority (73%) of the eruptions in this compilation became younger once calibrated to the IntCal20 curve, with only 7 (16%) becoming older following recalibration (Fig. S4). Five eruptions (11%) showed no differences in age between IntCal04 and IntCal20. Recalibration increased the precision of all eruption ages within the compilation; the largest error reduction was exhibited by the Wakamiko eruption of Aira caldera, Japan (−2566 years). All newly recalculated ages and uncalibrated radiocarbon dates used for these calculations are reported in Table S1, and we include raw data and graphical outputs in a supplementary file.

Four eruptions did not need recalibration, because their ages are derived solely from ice-core tephrochronology. This meant that either (a) no eruptive material has been directly dated by ^14^C, or (b) that the range in existing radiocarbon age determinations was so large that no one of these could be isolated for calibration with confidence. The Eldgjá eruption of the Katla volcano (1.011 ± 0.001 ka) also presents a unique case for inclusion. Although this event did not meet the selection criteria for minimum magnitude described above, this eruption is thought to have been possibly the largest flood basalt eruption on Earth over the last two millennia, with ∼85% of its SO_2_ emissions (~220 Tg) reaching upper tropospheric and lower stratospheric altitudes^104,105^. Therefore, it represents a rare case where SO_2_ emissions may be substantial, but released over a more prolonged period of time than is typical for an explosive eruption^106^.

To complement our geological eruption record, we also consider the HolVol eruption record produced by Sigl et al.^31^, which is based on high-resolution ice-core SO_4_ (or S) measurements from Greenland and Antarctic ice cores. The volcanic reconstruction is based on four ice cores, all synchronised to the annual-layer counted WAIS Divide (WD)2014 chronology from Antarctica. This timescale used the following eruptions as age constraints: Tambora (Indonesia), Huaynaputina (Peru), Samalas (Indonesia) and Miyake (Japan). The Greenland ice-core used in this study is additionally constrained using documentary records of volcanic dust veils in ~1.41, 1.32, and 1.01 ka as described by Stothers and Rampino^107^ and Sigl et al.^9^, together with the eruption markers described above. No climate information has been used for the chronologies, and no external age constraints have been used before 1.41 ka in either Greenland or Antarctica, but the WD2014 is in excellent agreement (±15 years) with dendrochronological dates throughout the Holocene (e.g., ^10^Be and ^14^C)^9,31^.

Not all the largest peaks in volcanic stratospheric sulphur (VSSI) and stratospheric aerosol optical depth (SAOD) listed in HolVol can (yet) be definitively linked to a source volcano. However, this ice-core-based reconstruction can directly complement our geological dataset. For example, by capturing signals produced by eruptions that left minimal or no terrestrial deposits^35,49^. HolVol identifies 850 discrete eruptions with sulphur emissions >1 teragram (Tg) between 11.5 and 0.05 ka. In other words, eruptions that equal or exceed the sulphur output of the 2011 CE Nabro (Eritrea; M4.4) and 2008 CE Kasatochi (Alaska; M4.3) eruptions, and so likely to have had a measurable effect on the climate system^52,108^. Within HolVol, there exist 187 VSSI anomalies >10 Tg S, and 11 with ascribed VSSI values > 60 Tg S. Hence, quantities of SO_4_ comparable to net emissions by the 1991 CE Pinatubo (M6.1; ~8.5 Tg S), and 1257 CE (Samalas) (M7; ~75 Tg S) eruptions, respectively.

### Statistical analysis

To test whether volcanic eruptions were a candidate trigger for Holocene cold events, we perform statistical analysis of databases for the timing of glacial advances and the largest (*M* ≥ 7) eruptions known to have occurred during this interval. The time-association between our eruption and glacial advance datasets is tested in a stepwise manner, relative to two core hypotheses: (1) if explosive volcanism is causally linked to Holocene glacial advances, eruptions dated to that period occur in close proximity to major cooling events more often than would a randomised set of the same number of eruptions and (2) this association would be likely to be dominated by Northern Hemisphere eruptions, given that glacial advances of the Holocene are known to be intrinsically linked to ocean-atmosphere dynamics in the North Atlantic^109^—thus are most likely to be influenced by extratropical eruptions in that same hemisphere^20,110^. Both can be tested using a relatively simple statistical test of an association.

Here, we consider the largest (*M* ≥ 7) eruptions within our compilation (*n* = 11). This equates to ~one eruption per kiloyear, which is a frequency consistent with statistical modelling^111^. This may result in the omission of climatologically significant eruptions; however, using too many eruptions would artificially inflate the likelihood of a false-positive result where no actual link between an eruption and the climate response exists. Our method increases the likelihood that any identified link between an eruption that passed through this filtering and a glacial advance is meaningful, but also that some glacial advances were caused by eruptions that were filtered out. Hence, our null hypothesis posited that known eruptions coincide with glacial advances no more closely in time than they would by chance alone.

To test this, we first captured the average time gap between a given glacial advance and the closest eruption date by using the root-mean-square (RMS) best match statistic (1) of all such time gaps in the record...

1 $${\mathrm{RMS}}=\sqrt{1/{{\mathrm{N}}}_{\mathrm{e}}{\sum }_{{\mathrm{k}}=1}^{{{\mathrm{N}}}^{\mathrm{e}}}{\min }_{\mathrm{j}}({{\mathrm{T}}}_{\mathrm{j}}^{\mathrm{c}}-{{{\mathrm{T}}}_{{\mathrm{k}}}^{{\mathrm{e}}}})^{2}}$$

…where the RMS is a function of *N*_e_ (the number of eruptions), ${{\mathrm{T}}}_{{\mathrm{k}}}^{{\mathrm{e}}}$ (the eruption dates) and ${{\mathrm{T}}}_{\mathrm{j}}^{\mathrm{c}}$ (the glacial advance dates), and provides a summation of the distances between individual eruption ages and the nearest glacial advance.

The probability distribution and cumulative distribution functions of this statistic were then assessed by generating 10 million random eruption dates (*E*_R_), with the assumption that these dates were uniformly and independently distributed across the interval of interest (~11.7–0.6 ka). As part of this, we compared *N*_e_ = 11 to the RMS for *E*_R_ with *N*_e_ = 11; meaning that the 1 million MC ensembles produced the same number of eruptions per simulation (*n* = 11) as there were actual Holocene eruptions as specified above (*n* = 11). From this, the probability that the RMS best match statistic for the distribution of the actual ages (*E*_A_) was significantly less than for *E*_R_ was determined. This means that if no correlation exists between the *E*_A_ dates and climate shifts, the sum of all the RMS best match statistics should be no different from that produced using *E*_R_ dates. For our analysis, we define *E*_A_ by dates calibrated to the IntCal20 curve, which is the most up-to-date radiocarbon calibration currently available^46^. Information for all calibrations is available in the supplementary file accompanying this article.

## Supplementary information

Supplementary Information

Transparent Peer Review file

## Source data

Source Data

## Data Availability

All of the eruption ages recalibrated by this study (IntCal20) are included in the Supplementary Dataset and Information files that accompany this article. Also included are eruption source locations, uncalibrated ^14^C ages, and their associated references. Secondary datasets used in this study are all accompanied by reference to the source literature and/or repository in which the data are stored. Source data are provided with this paper.
