# Supplementary material for: Evidence for volcanic forcing of Holocene cold events
Source: Nat Commun. 2026 May 28;17:6950. doi: 10.1038/s41467-026-73492-4 (PMC13388704; doi:10.1038/s41467-026-73492-4)
Supplement: Supplementary file 1 — Supplementary Information [file 41467_2026_73492_MOESM1_ESM.pdf]

# Evidence for volcanic forcing of Holocene cold events

Alice R Paine[1]*, James UL Baldini[2], Charlie L Rex[3], Michael Sigl[4,5], Francesco S R Pausata[6], Richard J Brown[2]

[1]Department of Environmental Sciences, University of Basel, CH-4056 Basel, Switzerland
[2]Department of Earth Sciences, Durham University, Durham, DH1 3LE, UK
[3]Department of Earth Sciences, University of Oxford, Oxford, OX1 3AN, UK
[4]Department of Geosciences, University of Oslo, Oslo, Norway
[5]Oeschger Centre for Climate Change Research, University of Bern, CH-3012 Bern, Switzerland
[6]Centre ESCER (Étude et la Simulation du Climat à l'Échelle RÉgionale) and GEOTOP (Research Centre on the dynamics of the Earth System), Department of Earth and Atmospheric Sciences, University of Quebec in Montréal, Montréal, Canada

***Corresponding Author:*** alice.paine@unibas.ch

## S1: Volcano-climate interactions

Explosive volcanic eruptions emit volatile gases and halogens in variable concentrations, exerting complex effects on atmospheric chemistry and temperature[1]. The lifetime and spatial spread of volcanic compounds in the upper atmosphere are governed by factors including: (a) the latitude of the point source, where aerosols emitted at lower latitudes are more likely to spread between hemispheres[2,3]; and/or (b) the time of year of the eruption, due to changes in tropopause height and the location of the ascending branch of the Hadley circulation that impacts poleward transport and its dispersion[4,5].

Net sulphur dioxide ($SO_2$) loading into the stratosphere is a critical control on the short-term climatic significance of an eruption. Oxidation in the atmosphere leads to formation of sulphate aerosols, which scatter incoming solar shortwave radiation, thereby reducing the amount of energy reaching the Earth's surface and producing rapid cooling[6]. However, the relationship between eruption magnitude and $SO_2$ erupted mass is also modulated by magma composition and by factors such as sulphur solubility and removal (or "scrubbing") by hydrothermal systems and other fluids[7–9]. Volcanogenic sulphur can influence climate primarily through the formation of stratospheric sulphate aerosols, and this impact can arise either from a single, large, sulphur-rich eruption or from a cluster of moderate-magnitude eruptions occurring over short time intervals. A classic example of the former is the 1815 eruption of Tambora (Indonesia), which produced significant cooling through a strong negative radiative forcing — estimated at around -2 to -3 W/m² global annual mean[3,10]. This was considerably larger than the estimated solar forcing reduction during the Maunder Minimum (1645–1715 CE), which was on the order of -0.2 W/m² (IPCC AR6). However, closely timed eruptions forming a cluster can have even greater long-term climatic effects, due to sustained stratospheric aerosol loading and reduced recovery time between events. In these instances, the sulphur dose (and thus climate forcing) would vary in relation to eruption-specific factors such as duration and degassing intensity, as well as the tightness of the cluster[11].

Beyond radiative cooling, explosive volcanism can also induce significant dynamical changes in the climate system by disrupting atmospheric circulation patterns through the injection of sulphur dioxide and other compounds into the stratosphere[12]. For eruptions that occur in extra-tropical and polar regions, these compounds are typically concentrated within the origin hemisphere [13–15], whereas those emitted by tropical eruptions exhibit less pronounced north-south partitioning[3]. In all cases, hemispheric

asymmetries in aerosol-induced cooling – for instance, stronger cooling in the hemisphere where the eruption occurs – create an interhemispheric energy imbalance. This forces the intertropical convergence zone (ITCZ) to shift toward the relatively warmer hemisphere, triggering major reorganizations of atmospheric circulation and precipitation patterns [4]. This atmospheric response to volcanism is well documented by both observational and model-based evidence. For example, a strengthening of the Northern Hemisphere stratospheric polar vortex was observed following the eruption of Mount Pinatubo in 1991 CE [16], contributing to a positive phase of the North Atlantic Oscillation (NAO) and associated shifts in midlatitude climate patterns. A growing suite of coupled Earth and climate system models further suggests that volcanic eruptions can lead to sea ice expansion, followed by changes in deep ocean convection and Atlantic Meridional Overturning Circulation (AMOC) strength in the subsequent years [17–20].

Volcanic-induced climate cooling varies widely in both amplitude and duration depending on multiple factors including: (1) eruption magnitude; (2) stratospheric $SO_2$ load and its injection rate; (3) eruption latitude; and (4) background climate conditions[3,21,22]. The first three of these are eruption specific and reflect key volcanic parameters such as total erupted volume, magmatic volatile content, and magma composition. Despite this variability, compilations of explosive volcanic eruption timings, locations, and magnitudes – together with refinements in paleoclimate time-series from diverse climate proxies – increasingly support the proposition that explosive volcanism has repeatedly triggered annual-to-decadal scale cooling episodes throughout the Common Era (~2.5 ka to present) [11,18,23,24]. Building on these observations, several studies suggest that feedbacks within the coupled atmosphere–ocean system could prolong the climate effects of explosive eruption(s) beyond the immediate aerosol phase. Such feedbacks may contribute to centennial-scale cooling events observed throughout the Holocene [11,17–20,25].

## S2: Cold event definition

In this study, we define the timing of abrupt Holocene cooling events by the onsets of major glacial advances, derived from compilation of radiometric ages acquired from moraines across 17 regions compiled by Solomina et al.[26] (**Fig. S1**). Two secondary sources of information were also considered for definition of these cold events: (1) temperature reconstructions derived from argon and nitrogen isotopes contained within air trapped inside the GISP2 ice core[23], and (2) sedimentary records of ice-rafted debris deposition in the North Atlantic[27]. Here, we outline why both of these sources were deemed unsuitable for our intended purpose.

The ice core-derived temperature reconstruction presented in Kobashi et al.[23] is derived from isotopic measurements of occluded air in the GISP2 ice core (Greenland). From this time series, the coldest events can be isolated by calculating residuals from a 15-point moving average, which highlighted the events defined as data with values more than 0.2 °C below the moving average (n = 28). However, despite the clear temporal agreement of this record with the VSSI/SAOD curves (**Fig. S2**)[28], the decision was made not to use this data to define the cold events in our analysis. First, because it could introduce circularity to the result if tested against (and compared with) the ice core-derived sulphur record,

especially in cases where the best eruption age currently available is based on ice core data. Second is because the rate and magnitude of cooling recorded in Greenland may not be representative of the wider Northern Hemispheric signal[29], and is instead more likely to reflect regional-scale atmospheric circulation patterns and sea-ice feedbacks. As a result, reliance on a single Greenland-based temperature proxy could bias the identification of cold events toward regional-scale responses, rather than capturing broader hemispheric-scale climate variability.

Another factor to consider is the annual- to decadal-scale resolution of this argon-nitrogen temperature reconstruction[23]. Precision that is high enough to capture sub-decadal-scale high-latitude temperature excursions, and so highly effective for considering the short-term (<$10^1$ years) climate effects of explosive eruptions (e.g., seasonal anomalies). This suitability is reinforced by a statistically significant time association between the largest high-latitude temperature (cooling) excursions[23] , and VSSI peaks[28]; when tested also using an RMS-based approach. However, a record with such high resolution is less appropriate for examination of more prolonged climate responses to volcanism; where non-linear and/or mid-low latitude processes could cause the systemic response to last significantly longer than the radiative forcing itself[30]. For example, surface temperature relaxation times are significantly longer in the ocean compared to the land[20,31], meaning that the first measurable signs of perturbation may not become measurable for several months after the peak in radiative forcing[4]. Hence, the high temporal sensitivity of this proxy may overemphasize short-lived, regionally confined cooling anomalies while underrepresenting the integrated, longer-term climate response to volcanic forcing.

Bond (ice-rafting) Events are also pervasive features of Holocene climate variability[32]. However, they are also unsuitable here as markers of abrupt Holocene cold events. Although their associated peaks in the established North Atlantic drift-ice index[27] do correspond to several of the glacial advances[26], they are not consistent features of these advances. However, absence of evidence does not equate to evidence of absence, and because Bond Events are defined by marine sediment tracers, it is highly possible that the ice rafting events did not reach the coring locations, or the resulting glacial advance was not sufficiently large in magnitude for ice rafting in the first place. Both could result in an abrupt cooling event failing to be recorded in the sedimentary record.

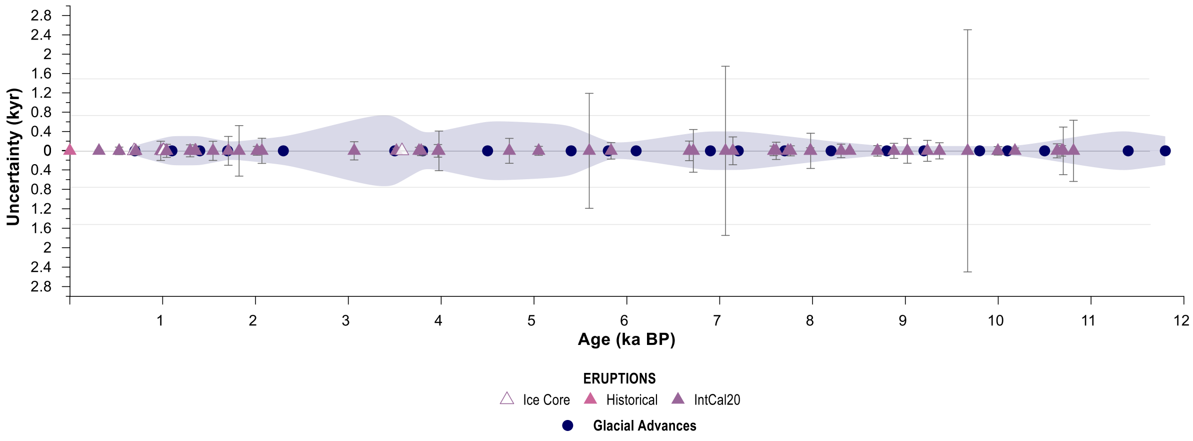


**Figure S1**: Uncertainties in glacial advance and eruption dates. Glacial advance dates are based on the compilation by Solomina et al.[26] , with blue shading marking upper and lower uncertainty estimates. Eruption dates derive from various sources (all listed in **Table S1**), and error bars mark the range in ages given in this study.

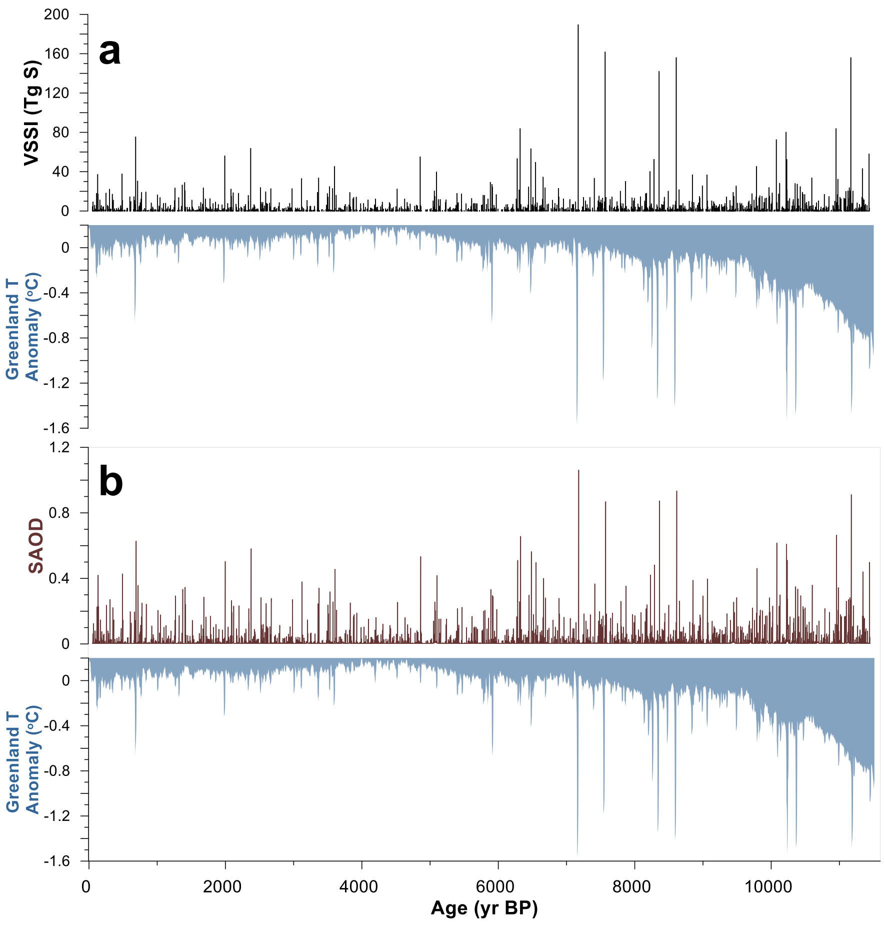


**Figure S2**: Comparison of reconstructed Greenland temperature anomalies over the Holocene derived from argon and nitrogen isotopes[23], with (a) volcanic stratospheric sulphur injection (VSSI) and (b) stratospheric aerosol optical depth (SAOD)[28].

## S3: Paleo-volcanic records

Few large (magnitude (M)≥6) eruptions have been directly observed, and even fewer have been empirically documented [33]. This means that assessing the timing, frequency, and magnitude of explosive volcanism during the Holocene relies on the availability of palaeovolcanic records, which can be divided into two categories. The first are databases, where geological information on discrete eruption events can be organized, stored, and combined with other studies focussing on the same event. Examples of information typically stored in these databases include the name of the volcano, specific location, magnitude, volume of ejecta, and magmatic composition (e.g., LaMEVE[34]). The second type are those in which eruption events are identifiable as a discrete geochemical, and/or stratigraphic signals within a natural archive: signals that can be used to determine the timing of eruption events relative to any environmental perturbations simultaneously captured within the record.

Ice cores from Greenland and Antarctica have become crucial archives of past volcanic activity[28]. Explosive eruptions inject large quantities of 'impure materials' into the atmosphere, with direct effects on atmospheric opacity and composition [35]. Given the known purity of precipitation falling on the high latitude ice sheets, and the low background impurity level of the ice, contamination of the atmosphere by volcanic tephra and aerosols can produces measurable, short-lived changes in the composition of the ice at the surface[36,37]. Specifically, as discrete peaks in sulphate ($SO_4$) concentrations [38–40], and/or microscopic (crypto-) tephra particles[41,42]. In the 1980's, pioneering studies first identified acidic layers within ice cores from Greenland temporally coinciding with major 20$^{th}$ century eruptions[36]. Building on this methodology, recent extension of chemostratigraphic records has since provided continuous records of explosive volcanism spanning the Common Era (2.5 ka to present[38]), and more recently the full Holocene[28,40]. Following the broad assumption that the ice-core sulphate deposition is proportional to the stratospheric sulphur emission, proxies to more accurately quantify the radiative effects of volcanic aerosols on the Earth's atmosphere through time have subsequently been developed: stratospheric sulphur injection (VSSI), and stratospheric aerosol optical depth (SAOD) (**Fig. S2**)[43].

The information provided by ice cores has been invaluable for the study of volcano-climate interactions during the Holocene[28,40]. Sulphur dioxide ($SO_2$) is also arguably the most significant gas emitted from volcanoes from a short-term climatological perspective, owing to its ability to cause measurable perturbations to the Earth's atmosphere[44]. However, there are several degrees of uncertainty associated with records of eruption-specific sulphur emissions on longer ($10^3$-yr) timescales. These uncertainties underpin our decision to not define the 'largest' Holocene eruption events using peaks in SAOD and/or VSSI (proxy for $SO_2$ output). Thus, while acknowledging the fact that erupted mass and sulphur yield is not one-to-one[6], eruption magnitude provides a practical, consistent, and reproducible proxy for eruption scale[45], and subsequently climate forcing potential, that can be used for comparative purposes.

The first source of uncertainty relates to measurement of pre- and syn-eruptive sulphur emissions of past eruptions, for which there is no single technique ideally suited to this purpose. For eruptions that

occurred in the pre-instrumental era, sulphur emissions may be estimated using ice-core based reconstructions[28,38], or by petrological analysis of melt inclusions trapped in volcanic rocks[6,46]. However, evidence exists for a clear discrepancy between predicted and measured sulphur emissions; a mismatch termed the "sulphur excess problem"[47]. This discrepancy was highlighted during our compilation process, where we compared estimates of erupted mass and, where available, sulphur output across the available literature for each discrete eruption. We found that the intra-study range in volume estimates (relative to the mean) was notably smaller than the ranges quoted for sulphur emissions; the latter largely due to poor consistency in estimates across different methodologies[47,48]. Thus, suggesting that placing quantitative constraints on an eruption's estimated sulphur emission is highly sensitive to methodology. Furthermore, ice cores retain $SO_4$ from both documented and undocumented volcanic eruptions[28,49], and so provide incomplete information on eruption source parameters that could be critical for understanding how these events could be mechanistically linked to long-term climate perturbations[28,38].

The second reason we define eruption size by volumetric mass, rather than sulphur output, is because selecting events based on the latter would risk introducing two key issues to our analysis: (1) temporal bias, and (2) data non-uniformity. The first issue refers to a bias toward more closely studied, but not necessarily larger, events. The amount of sulphur released is not known for all Holocene eruptions because of (a) poor access to eruptive materials suitable for petrological analysis, and/or (b) no conclusive evidence (e.g., tephra) to attribute the eruption to an ice core sulphur signal. For example, no studies have yet attempted to quantify sulphur emissions from the Tao Rusyr eruption (8.31 ± 0.14 ka), and no tephra has yet been found in ice cores from Greenland that would link this eruption to a definitive sulphur peak. If we were to filter our eruption compilation eruptions based solely on sulphur emission, this event would be eliminated; despite being one of the largest explosive eruptions of the Holocene[34]. Hence, preferentially excluding under-characterised events, while also overrepresenting those that have been more intensively studied.

Data non-uniformity is another issue that could arise if the largest Holocene eruptions are isolated solely by VSSI/SAOD. For example, **Figure S2** shows that the largest SAOD and VSSI peaks are non-uniformly distributed, with 8 of the 10 largest VSSI injections recorded between ~9 and 6 ka. VSSI values for all eight of these eruptions exceed those calculated for the largest known volcanic eruptions of the last 2.5-kyr (excluding the 1257 CE Samalas eruption[50]). Conversely, between ~6 and 0 ka, both the average number of eruptions and the cumulative VSSI (45 Tg S $kyr^{-1}$) appear 21% and 41% lower, respectively[28]. To assess the effects of this non-uniformity on our analysis, we performed an additional root-mean-square (RMS) test of the time-association between the largest SAOD 'eruptions'[28], and global glacial advances[26]. This analysis yielded a non-significant result, and we posit that this is most likely due to the fact that the glacial advance onsets defined by Solomina et al.[26] extend near-uniformly across the entire Holocene, similar to the eruption record defined by volumetric mass (**Fig. S1**). Whereas, the largest SAOD and VSSI peaks are restricted to the first half of the Holocene (**Fig. S2**), resulting in a temporal mismatch that directly affects the statistical power of the test. For example, 'synthetic' eruptions are generated under the assumption of temporal randomness across the full

Holocene window, producing a near-uniform distribution of events through time. However, because the largest SAOD peaks appear concentrated into the ~9–6 ka window, the Monte Carlo simulations systematically overestimate the expected temporal overlap with glacial advances under the null hypothesis: inflating the baseline coincidence level, and creating disproportionately large temporal gaps. Hence, explaining why glacier advance[26] onsets better correlate with eruptions identified from geological evidence (**Table S1**), but produce a non-significant result when tested against the largest VSSI/SAOD peaks[28].

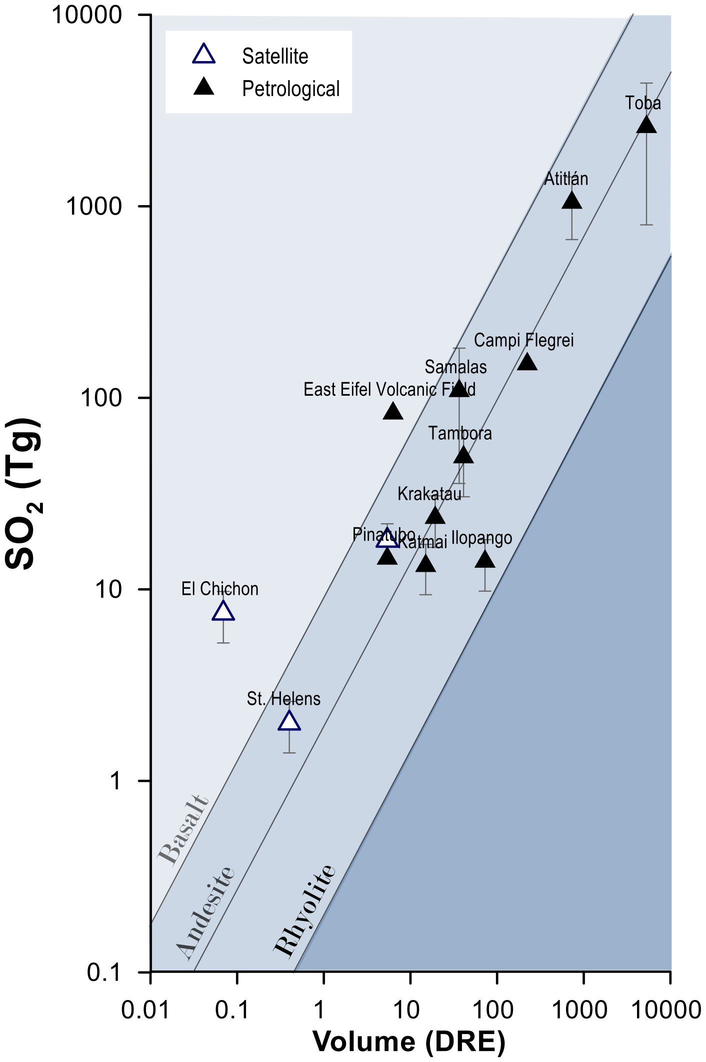


**Figure S3**: Erupted volume (dense-rock equivalent (DRE)) versus $SO_2$ flux (Tg) plotted for a selection of large volcanic eruptions known to have occurred over the past ~100-kyr. Eruptions for whom $SO_2$ values have been calculated using petrological methods are marked as black triangles. Direct satellite-derived values are marked by white triangles. Blue shading highlights the three primary bulk magma compositions as defined by sulphur solubility[46].

## S3.1. Eruption dating

**Table S1**: Uncalibrated ($^{14}C$), calibrated (IntCal20), and ice core-derived ages for the largest known Holocene eruptions. IntCal20 ages (marked by dark blue) listed are those calculated by, and presented in this study. All other information is listed alongside the corresponding reference(s). All ages are given in years before present, where “present” corresponds to 1950 CE. Eruptions shaded in grey are those used in the RMS analysis.

| Volcano | Latitude | Eruption Unit | M | $^{14}C$ Age | Ref | IntCal20 Age* | Ice Core Age | Ref |
|---|---|---|---|---|---|---|---|---|
| Aira | 31.59 | Wakamiko | 6.1 | 8050±1000 | 51 | 9671±2506 | - | - |
| Ambrym | -16.25 | ~2ka | 6.8 | 1900±106 | 52 | 2069±260 | - | - |
| Aniakchak | 56.88 | Aniakchak II | 6.9 | - | - | - | 3578±4 | 53 |
| Aniakchak | 56.88 | Aniakchak I | 6.5 | 9470±40 | 54 | 10686±109 | - | - |
| Avachinsky | 53.26 | IAv2 | 6.0 | 7151±51 | 55 | 7975±63 | - | - |
| Black Peak | 56.55 | CF | 6.5 | 4447±40 | 56 | 5050±93 | - | - |
| Ceboruco | 21.13 | Jala Pumice | 5.9 | 1055±85 | 57,58 | 980±199 | 1009±1 | 59 |
| Changbaishan | 41.98 | Millennium/B-Tm | 7.4 | - | - | - | 1004±1 | 60 |
| Churchill | 61.38 | WRAe | 6.7 | 1097±63 | 58,61 | 1044±136 | 1097±1 | 38,62 |
| Churchill | 61.38 | WRAn | 6.2 | 1822±130 | 63 | 1708±299 | - | - |
| Dakataua | -5.06 | Dk | 7.4 | 1370±37 | 64 | 1349±51 | - | - |
| Fisher | 54.65 | CF | 6.7 | 9372±198 | 65 | 10701±493 | - | - |
| Fisher | 54.65 | Turquoise Cone | 6.2 | 5120±70 | 65 | 5830±170 | - | - |
| Grímsvötn | 64.42 | Saksunarvatn | 6.6 | - | - | - | 10180±20 | 28 |
| Illopango | 13.67 | TBJ | 6.7 | 1650±102 | 66 | 1542±198 | 1519±2 | 66 |
| Kaguyak | 58.61 | Caldera eruption | 5.6 | 5800±200 | 67 | 6716±442 | - | - |
| Karkar | -4.65 | Wadau deposit | 6.0 | 9090±250 | 68 | 10811±633 | - | - |
| Karymsky | 54.05 | KRM | 6.2 | 7892±41 | 69 | 8700±109 | - | - |
| Katla | 63.63 | Eldgjá | 5.7 | - | - | - | 1011±1 | 59,70 |
| Khangar | 54.75 | KHG | 6.2 | 6915±30 | 69,71 | 7735±60 | 7872±50 | 72 |
| Kikai | 30.79 | Akahoya/ K-Ah | 7.2 | 6280±130 | 73 | 7142±288 | - | - |
| Krakatau | -6.10 | 416 AD | 7.1 | 1609 | - | - | - | - |
| Ksudach | 51.80 | KS1 | 6.3 | 1850±240 | 58,74 | 1824±523 | - | - |
| Ksudach | 51.80 | KS2 | 5.9 | 7133±195 | 58,74 | 7980±364 | 7089±26 | 42 |
| Kurile Lake | 51.45 | CF | 7.3 | 7618±14 | 75 | 8402±22 | - | - |
| Long Island | -5.36 | Biliau Beds | 6.3 | 3990±110 | 76 | 4734±258 | - | - |
| Lvinaya Past | 44.61 | CF | 7.0 | 9400±60 | 77 | 10634±145 | - | - |
| Makushin | 53.89 | 6000 BC | 5.9 | 8050±50 | 51 | 8877±156 | - | - |
| Mashu | 43.57 | Ma-f/g/h/i/j | 6.3 | 6730±60 | 78 | 7582±99 | 7473±33 | 42 |
| Mazama | 42.93 | Crater Lake | 6.8 | 6754±100 | 79 | 7609±179 | 7562±35 | 42 |
| Okmok | 53.43 | Okmok I | 6.9 | 8260±90 | 56 | 9237±217 | - | - |
| Okmok | 53.43 | Okmok II | 6.7 | 2050±30 | 80 | 2017±92 | 1993±2 | 81 |
| Opala | 52.54 | OP | 5.7 | 1480±50 | 69 | 1357±64 | - | - |
| Oshima-Oshima | 41.51 | Nishi-yama ejecta | 6.0 | 760±70 | 82 | 710±87 | - | - |

| | | | | | | | | |
|---|---|---|---|---|---|---|---|---|
| Pago | -5.58 | WK-2 | 6.5 | 3300±160 | 83 | 3976±410 | - | - |
| Pago | -5.58 | WG | 6.4 | 1240±80 | 83 | 1296±124 | - | - |
| Pago | -5.58 | WK-1 | 6.0 | 5630±110 | 83 | 6672±203 | - | - |
| Pinatubo | 15.13 | Tayawan/ Pasbul | 6.3 | 8380±80 | 84 | 9367±167 | - | - |
| Pinatubo | 15.13 | Crow Valley | 6.1 | 4865±485 | 84 | 5595±1190 | - | - |
| Pinatubo | 15.13 | Maraunot | 6.1 | 2925±70 | 84 | 3063±188 | - | - |
| Rabaul | -4.27 | Rabaul Pyroclastics | 6.0 | 1380±34 | 85 | 1351±42 | - | - |
| Rinjani | -8.42 | Samalas | 7.0 | - | | - | 693±1 | 50 |
| Santorini | 36.40 | Minoan | 7.3 | 3310±23 | 86 | 3518±53 | - | - |
| Semisopochnoi | 51.93 | Caldera formation | 7.1 | 6920±60 | 87 | 7764±104 | - | - |
| St. Helens | 46.20 | Yn (Smith Creek) | 6.0 | 3499±40 | 88 | 3785±99 | - | - |
| Taal | 14.00 | Taal scoria | 6.7 | 6110±800 | 89 | 7063±1751 | - | - |
| Tao-Rusyr | 49.35 | CF | 7.0 | 7500±80 | 69 | 8308±140 | - | - |
| Ulleungdo | 37.50 | U4 | 6.7 | 8949±42 | 90 | 9997±88 | - | - |
| Veniaminof | 56.17 | CF | 6.7 | 3646±55 | 56 | 3965±131 | - | - |
| Vesuvius | 40.82 | Avellino | 6.5 | 3479±29 | 91 | 3761±74 | - | - |
| Vesuvius | 40.82 | Mercato Pumice | 5.7 | 8098±71 | 92,93 | 9022±257 | - | - |

**Inclusion of Aniakchak II in our statistical analysis follows an independent assessment of all eruptions listed in* ***Table S1****, where each event was assessed in order to verify volumetric estimations and their associated magnitude calculations. Despite the Aniakchak II having a LaMEVE-listed magnitude that falls just below our M≥7 criterion, its inclusion is justified for two reasons. First, the volcano is surrounded by ocean, the reported eruption size of 6.9 is likely a minimum estimate[94]. Second, LaMEVE calculates a magnitude of 6.9 using magma and tephra densities of 2500 kg $m^{-3}$ and 1000 kg $m^{-3}$, but using values more consistent with field evidence (2200 kg $m^{-3}$ and 1500 kg $m^{-3}$, respectively [95,96]) would increase the estimated magnitude to above 7.0.*

To ensure chronological consistency across our dataset, all eruption ages were first recalibrated using the IntCal20 curve[97], and modelled within the OxCal online version 4.4 model (https://c14.arch.ox.ac.uk/oxcal.html). To assess time-resolved changes in age precision as a function of refinements in radiocarbon age determinations, all $^{14}C$ eruption ages were also calibrated to previous iterations of the radiocarbon curve, each named based on the year of publication: IntCal13, IntCal09, and IntCal04. Ages ascribed to the majority (73%) of the eruptions in this compilation became younger once calibrated to the IntCal20 curve, with only 7 (16%) becoming older following recalibration (**Fig. S2**). Five eruptions (11%) showed no differences in age between IntCal04 and IntCal20, Recalibration increased the precision of all eruption ages within the compilation; the largest error reduction exhibited by the *Wakamiko* eruption of Aira caldera, Japan (-2566 years). All newly recalculated ages and uncalibrated radiocarbon dates used for these calculations are reported in **Table S1**.

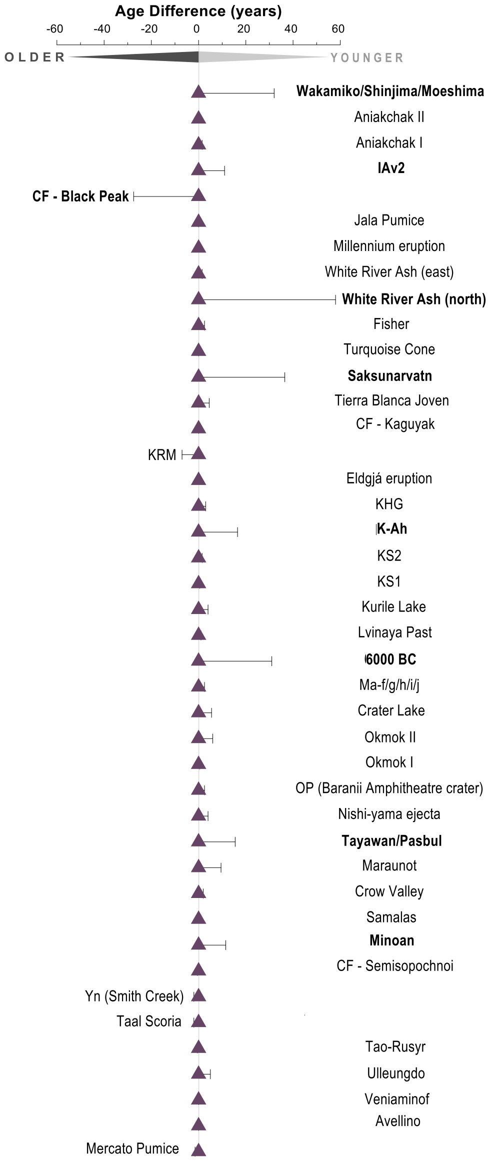


**Figure S4:** Age differences between IntCal04, and IntCal20 calibrations for all eruptions considered in this work. Eruptions are labelled by unit name.

## S4: Orbital (solar) forcing

Orbital forcing is known to have played a fundamental role in pacing low-amplitude climate variability during the Holocene[27,98,99]. Over multiple millennia, the climate system has been strongly influenced by opposite hemispheric trends of the summer insolation, with the consequent redistribution of energy prompting seasonal shifts in mean Intertropical Convergence Zone (ITCZ) position, and Northern Hemisphere summer monsoon strength [100–102].

On Holocene timescales, beryllium-10 ($^{10}Be$) records from ice cores have historically been a key source of evidence favouring solar output as the main driver of Holocene climate, and hence that low solar output (measured by total solar irradiance (TSI)) provides a robust explanation for abrupt cooling events[27,98]. However, what remains unclear is if, and how, solar variability could explain the more abrupt atmospheric and terrestrial signals associated with Holocene cold events[103]. First, because the

mechanisms that dictate the sensitivity of the Earth's climate to solar variability also remain poorly understood, as the relative importance of factors such as stratospheric winds[104], cloud condensation[105], the El Niño Southern Oscillation (ENSO)[106], the North Atlantic Oscillation (NAO)[107], winter westerly strength[108], and sea ice distributions[109] for amplification of solar signals is likely to vary spatially and temporally. This not only complicates efforts to isolate solar forcing from internal variability but also precludes correlation of short-lived signals between records, where solar anomalies may be finer than the chronological uncertainties in the archive's age model.

Another source of uncertainty in attribution of Holocene cold events to solar variations can also be linked to volcanism. Studies have shown that volcanic eruptions demonstrably affect the concentration of $^{10}Be$ in the atmosphere, by producing a heightened equator-pole temperature gradient that is associated with intensification of the northern polar vortex[14,110], and elevated drawdown of $^{10}Be$ from the stratosphere following volcanic eruptions[111,112]. For example, ice sheet $^{10}Be$ concentrations were found to be significantly increased following the 1257 CE Samalas eruption of Rinjani volcano (Indonesia), with the proposed mechanism for this increase being adsorption of $^{10}Be$ to $SO_4$, and subsequent depletion of the stratospheric $^{10}Be$ reservoir[111]. Thus, if considered across the Holocene, it is possible that $^{10}Be$ removal by volcanogenic sulphate aerosols following large eruptions could also account for observed increases in ice sheet $^{10}Be$ during Holocene cold events. Hence, it is crucial that future work further tests how volcanism contributed to the origins of decadal- to centennial-scale cosmogenic isotope variability derived from ice cores.

## S5: Role of background conditions

Background conditions provide essential context for understanding both the causes and expressions of Holocene climate change. Although the Holocene is often viewed as a period of relative climatic stability, gradual changes in orbital-driven insolation are known to have influenced these conditions, and hence the expressions of climate variability during this time. These include the distribution of solar radiation across latitudes and seasons, which in turn affected factors such as ice sheet dynamics, atmospheric circulation patterns, sea surface temperatures, and ocean-atmosphere heat exchange. Understanding these slow insolation changes is therefore crucial for understanding the mechanisms driving climate variability across different stages of the Holocene[99].

The extent to which orbital-scale changes may have amplified the sensitivity of the Holocene climate system to volcanic forcing remains unclear. Examination of climate variability during the last-glacial period (120–11 ka) has suggested that abrupt climate change tends to occur when the system is 'primed' for instability, often defined by high/intermediate global ice volume, and low/intermediate atmospheric $CO_2$[113]. It is unlikely the effect of these conditions was similarly pronounced during the Holocene, because (a) the timescales associated with climate variability were much shorter, and (b) abrupt changes occurred under interglacial conditions, with greater stability in atmospheric $CO_2$ and global ice volume[114]. Volcanic forcing could also have a distinctly different impact on a glacial climate system, in which atmospheric $CO_2$, global ice volume, sea level, and orbital forcing were significantly more

influential[115–117]. However, it is possible that more transient (<$10^3$-years) changes in background conditions during the Holocene could have influenced sulphate aerosol cycling, eruption plume altitude, and tropopause height, and so influenced the expression of any ensuing climate impact. All of which have been shown to influence the climatological potency of explosive volcanism, and studies suggest suggesting that these effects may be further amplified in an already-warming system[22,118]. Although there is a pressing need for quantitative constraints on how naturally forced variations in background climate conditions could influence where, and in what doses, volcanic $SO_2$ reaches the stratosphere, the evidence that does exist pertains to a non-trivial effect that is worthy of further exploration.

## References


1. von Glasow, R., Bobrowski, N. & Kern, C. The effects of volcanic eruptions on atmospheric chemistry. *Chemical Geology* **263**, 131–142 (2009).
2. Sjolte, J., Adolphi, F., Guðlaugsdòttir, H. & Muscheler, R. Major Differences in Regional Climate Impact Between High- and Low-Latitude Volcanic Eruptions. *Geophysical Research Letters* **48**, 1–8 (2021).
3. Toohey, M. *et al.* Disproportionately strong climate forcing from extratropical explosive volcanic eruptions. *Nature Geoscience* **12**, 100–107 (2019).
4. Erez, M. & Adam, O. Energetic Constraints on the Time-Dependent Response of the ITCZ to Volcanic Eruptions. *Journal of Climate* **34**, 9989–10006 (2021).
5. Pausata, F. S. R. & Camargo, S. J. Tropical cyclone activity affected by volcanically induced ITCZ shifts. *Proceedings of the National Academy of Sciences of the United States of America* **116**, 7732–7737 (2019).
6. Oppenheimer, C., Scaillet, B. & Martin, R. S. Sulfur degassing from volcanoes: Source conditions, surveillance, plume chemistry and earth system impacts. *Reviews in Mineralogy and Geochemistry* **73**, 363–421 (2011).
7. Shinohara, H. Excess degassing from volcanoes and its role on eruptive and intrusive activity. *Reviews of Geophysics* **46**, 1–31 (2008).
8. Wallace, P. J. Volatiles in subduction zone magmas; concentrations and fluxes based on melt inclusion and volcanic gas data. *Journal of Volcanology and Geothermal Research* **140**, 217–240 (2005).
9. Carn, S. A., Krotkov, N. A., Fisher, B. L. & Li, C. Out of the blue: Volcanic SO2 emissions during the 2021–2022 eruptions of Hunga Tonga—Hunga Ha'apai (Tonga). *Frontiers in Earth Science* **10**, 1–18 (2022).
10. Robock, A. Volcanic Eruptions and Climate. *Reviews of Geophysics* **38**, 191–219 (2000).
11. Van Dijk, E. J. C., Jungclaus, J., Sigl, M., Timmreck, C. & Krüger, K. High-frequency climate forcing causes prolonged cold periods in the Holocene. *Commun Earth Environ* **5**, 242 (2024).
12. Timmreck, C. Modeling the climatic effects of large explosive volcanic eruptions. *Wiley Interdisciplinary Reviews: Climate Change* **3**, 545–564 (2012).
13. Pausata, F. S. R., Zanchettin, D., Karamperidou, C., Caballero, R. & Battisti, D. S. ITCZ shift and extratropical teleconnections drive ENSO response to volcanic eruptions. 5194 (2020).
14. Fuglestvedt, H. F., Zhuo, Z., Toohey, M. & Krüger, K. Volcanic forcing of high-latitude Northern Hemisphere eruptions. *npj Clim Atmos Sci* **7**, 10 (2024).
15. Zhuo, Z., Kirchner, I., Pfahl, S. & Cubasch, U. Climate impact of volcanic eruptions: The sensitivity to eruption season and latitude in MPI-ESM ensemble experiments. *Atmospheric Chemistry and Physics* **21**, 13425–13442 (2021).
16. Stenchikov, G. *et al.* Arctic Oscillation response to the 1991 Mount Pinatubo eruption: Effects of volcanic aerosols and ozone depletion. *Journal of Geophysical Research Atmospheres* **107**, 4803 (2002).
17. Slawinska, J. & Robock, A. Impact of volcanic eruptions on decadal to centennial fluctuations of Arctic sea ice extent during the Last Millennium and on initiation of the Little Ice Age. *Journal of Climate* **31**, 2145–2167 (2018).
18. Miller, G. H. *et al.* Abrupt onset of the Little Ice Age triggered by volcanism and sustained by sea-ice/ocean feedbacks. *Geophysical Research Letters* **39**, 1–5 (2012).
19. Zhong, Y. *et al.* Centennial-scale climate change from decadally-paced explosive volcanism: A coupled sea ice-ocean mechanism. *Climate Dynamics* **37**, 2373–2387 (2011).
20. Pausata, F. S. R., Chafik, L., Caballero, R. & Battisti, D. S. Impacts of high-latitude volcanic eruptions on ENSO and AMOC. *Proceedings of the National Academy of Sciences of the United States of America* **112**, 13784–13788 (2015).
21. Mather, T., Pyle, D. M. Volcanic emissions: Short-term perturbations, long-term consequences and global environmental change. in *Volcanism and Global Environmental Change* (ed. Schmidt, A., Fristad, K., Elkins-Tanton, L.) 195–207 (Cambridge University Press, Cambridge, 2015).
22. Aubry, T. J. *et al.* Climate change modulates the stratospheric volcanic sulfate aerosol lifecycle and radiative forcing from tropical eruptions. *Nature Communications* **12**, (2021).

23. Kobashi, T. *et al.* Volcanic influence on centennial to millennial Holocene Greenland temperature change. *Scientific Reports* **7**, 1–10 (2017).
24. Larsen, D. J., Miller, G. H., Geirsdóttir, Á. & Ólafsdóttir, S. Non-linear Holocene climate evolution in the North Atlantic: a high-resolution, multi-proxy record of glacier activity and environmental change from Hvítárvatn, central Iceland. *Quaternary Science Reviews* **39**, 14–25 (2012).
25. Dutta, D. *et al.* State-Dependent North Atlantic Response to Volcanic Eruption Clusters. *Geophysical Research Letters* **52**, e2025GL117582 (2025).
26. Solomina, O. N. *et al.* Holocene glacier fluctuations. *Quaternary Science Reviews* **111**, 9–34 (2015).
27. Bond, G., Showers, W., Cheseby, M., Lotti, R., Almasi, P., de Menocal, P., Priore, P., Cullen, H., Hajdas, I., Bonani, G. A pervasive millennial-scale cycle in the North Atlantic Holocene and glacial climates. *Science* **294**, 2130–2136 (1997).
28. Sigl, M., Toohey, M., McConnell, J. R., Cole-Dai, J. & Severi, M. Volcanic stratospheric sulfur injections and aerosol optical depth during the Holocene (past 11 500 years) from a bipolar ice-core array. *Earth Syst. Sci. Data* **14**, 3167–3196 (2022).
29. McKay, N. P. *et al.* The 4.2 ka event is not remarkable in the context of Holocene climate variability. *Nat Commun* **15**, 6555 (2024).
30. Dima, M. & Lohmann, G. Evidence for Two Distinct Modes of Large-Scale Ocean Circulation Changes over the Last Century. *Journal of Climate* **23**, 5–16 (2010).
31. Stenchikov, G. *et al.* Volcanic signals in oceans. *Journal of Geophysical Research Atmospheres* **114**, 1–13 (2009).
32. Fletcher, W. J., Sánchez Goñi, M. F., Naughton, F. & Seppä, H. Introduction to the Holocene climate. in *European Glacial Landscapes* 65–72 (Elsevier, 2024). doi:10.1016/B978-0-323-99712-6.00001-5.
33. Brown, S. K. *et al.* Characterisation of the Quaternary eruption record: Analysis of the Large Magnitude Explosive Volcanic Eruptions (LaMEVE) database. *Journal of Applied Volcanology* **3**, 1–22 (2014).
34. Crosweller, H. S. *et al.* Global database on large magnitude explosive volcanic eruptions (LaMEVE). *Journal of Applied Volcanology* **1**, 1–13 (2012).
35. Lamb, H. H. Volcanic dust in the atmosphere; with a chronology and assessment of its meteorological significance. *Phil. Trans. R. Soc. Lond. A* **266**, 425–533 (1970).
36. Hammer, C. U., Clausen, H. B. & Dansgaard, W. Greenland ice sheet evidence of post-glacial volcanism and its climatic impact. *Nature* **288**, 230–235 (1980).
37. Hammer, C. U. Past volcanism revealed by Greenland Ice Sheet impurities. *Nature* **270**, 482–486 (1977).
38. Sigl, M. *et al.* Timing and climate forcing of volcanic eruptions for the past 2,500 years. *Nature* **523**, 543–549 (2015).
39. Wolff, E. W. *et al.* Frequency of large volcanic eruptions over the past 200 000 years. *Clim. Past* **19**, 23–33 (2023).
40. Cole-Dai, J. *et al.* Comprehensive Record of Volcanic Eruptions in the Holocene (11,000 years) From the WAIS Divide, Antarctica Ice Core. *Journal of Geophysical Research: Atmospheres* **126**, 1–15 (2021).
41. Plunkett, G. *et al.* Smoking guns and volcanic ash: The importance of sparse tephras in Greenland ice cores. *Polar Research* **39**, 1–11 (2020).
42. Davies, S. M. *et al.* Exploiting the Greenland volcanic ash repository to date caldera-forming eruptions and widespread isochrons during the Holocene. *Quaternary Science Reviews* **334**, 108707 (2024).
43. Toohey, M. & Sigl, M. Volcanic stratospheric sulfur injections and aerosol optical depth from 500 BCE to 1900 CE. *Earth System Science Data* **9**, 809–831 (2017).
44. Robock, A. Volcanic Eruptions and Climate. *Reviews of Geophysics* **38**, 191–219 (2000).
45. Pyle, D. M. Sizes of Volcanic Eruptions. in *Encyclopaedia of Volcanoes* (ed. Sigurdsson, H.) (Academic Press, 2015).
46. Wallace, P. J. Volatiles in subduction zone magmas; concentrations and fluxes based on melt inclusion and volcanic gas data. *Journal of Volcanology and Geothermal Research* **140**, 217–240 (2005).
47. Shinohara, H. Excess degassing from volcanoes and its role on eruptive and intrusive activity. *Reviews of Geophysics* **46**, 1–31 (2008).
48. Scaillet, B. & Oppenheimer, C. On the Budget and Atmospheric Fate of Sulfur Emissions From Large Volcanic Eruptions. *Geophysical Research Letters* **51**, e2023GL107180 (2024).
49. Lin, J. *et al.* Magnitude, frequency and climate forcing of global volcanism during the last glacial period as seen in Greenland and Antarctic ice cores (60–9 ka). *Climate of the Past* **18**, 485–506 (2022).
50. Vidal, C. M. *et al.* Dynamics of the major plinian eruption of Samalas in 1257 A.D. (Lombok, Indonesia). *Bull Volcanol* **77**, 73 (2015).
51. Global Volcanism Program. Volcanoes of the World. in *Volcanoes of the World* vol. 5.0.2 (Smithsonian Institution, 2023).
52. McCall, G. J. H., LeMaitre, R. W., Malahoff, A., Robinson, G. P. & Stephenson, P. J. The geology and geophysics of the ambrym caldera, New Hebrides. *Bull Volcanol* **34**, 681–696 (1970).
53. Pearson, C. *et al.* Geochemical ice-core constraints on the timing and climatic impact of Aniakchak II (1628 BCE) and Thera (Minoan) volcanic eruptions. *PNAS Nexus* **1**, pgac048 (2022).
54. VanderHoek, R. & Myron, R. *An Archaeological Overview and Assessment of Aniakchak National Monument and Preserve*. (2004).
55. Braitseva, O., Ponomareva, V. V., Sulerzhitsky, L. D., Melekestsev, I. V. & Bailey, J. Holocene Key-Marker Tephra Layers in Kamchatka, Russia. *Quaternary Research* **47**, 125–139 (1997).
56. Miller, T. P. & Smith, R. L. Late Quaternary caldera-forming eruptions in the eastern Aleutian arc, Alaska. *Geology* **15**, 434–438 (1987).

57. Sieron, K. & Siebe, C. Revised stratigraphy and eruption rates of Ceboruco stratovolcano and surrounding monogenetic vents (Nayarit, Mexico) from historical documents and new radiocarbon dates. *Journal of Volcanology and Geothermal Research* **176**, 241–264 (2008).
58. Jensen, B. J. L. *et al.* A latest Pleistocene and Holocene composite tephrostratigraphic framework for northeastern North America. *Quaternary Science Reviews* **272**, 107242 (2021).
59. Hutchison, W. *et al.* High-Resolution Ice-Core Analyses Identify the Eldgjá Eruption and a Cluster of Icelandic and Trans-Continental Tephras Between 936 and 943 CE. *JGR Atmospheres* **129**, e2023JD040142 (2024).
60. Oppenheimer, C. *et al.* Multi-proxy dating the 'Millennium Eruption' of Changbaishan to late 946 CE. *Quaternary Science Reviews* **158**, 164–171 (2017).
61. Jensen, B. J. L. *et al.* Transatlantic distribution of the Alaskan White River Ash. *Geology* **42**, 875–878 (2014).
62. Mackay, H. *et al.* The 852/3 CE Mount Churchill eruption: examining the potential climatic and societal impacts and the timing of the Medieval Climate Anomaly in the North Atlantic region. *Clim. Past* **18**, 1475–1508 (2022).
63. Reuther, J., Potter, B., Coffman, S., Smith, H. & Bigelow, N. Revisiting the Timing of the Northern Lobe of the White River Ash Volcanic Event in Eastern Alaska and Western Yukon. *Radiocarbon* **62**, 169–188 (2020).
64. McKee, C. O., Neall, V. E. & Torrence, R. A remarkable pulse of large-scale volcanism on New Britain Island, Papua New Guinea. *Bull Volcanol* **73**, 27–37 (2011).
65. Stelling, P., Gardner, J. E. & Begét, J. Eruptive history of Fisher Caldera, Alaska, USA. *Journal of Volcanology and Geothermal Research* **139**, 163–183 (2005).
66. Smith, V. C. *et al.* The magnitude and impact of the 431 CE Tierra Blanca Joven eruption of Ilopango, El Salvador. *Proceedings of the National Academy of Sciences* 202003008 (2020) doi:10.1073/pnas.2003008117.
67. Fierstein, J. Explosive eruptive record in the Katmai region, Alaska Peninsula: an overview. *Bull Volcanol* **69**, 469–509 (2007).
68. Pain, C. F. & McKee, C. O. Late Quaternary eruptive history of Karkar Island. *Geological Survey of Papua New Guinea Memoir* **10**, 39–47 (1981).
69. Braitseva, O. A., Melekestsev, I. V., Ponomareva, V. V. & Sulerzhitsky, L. D. Ages of calderas, large explosive craters and active volcanoes in the Kuril-Kamchatka region, Russia. *Bulletin of Volcanology* **57**, 383–402 (1995).
70. Oppenheimer, C. *et al.* The Eldgjá eruption: timing, long-range impacts and influence on the Christianisation of Iceland. *Climatic Change* **147**, 369–381 (2018).
71. Bazanova, L. I., Melekestsev, I. V., Ponomareva, V. V., Dirksen, O. V. & Dirksen, V. G. Late Pleistocene and Holocene volcanic catastrophes in Kamchatka and in the Kuril Islands. Part 1. Types and classes of catastrophic eruptions as the leading components of volcanic catastrophism. *Journal of Volcanology and Seismology* **10**, 151–169 (2016).
72. Cook, E. *et al.* First identification of cryptotephra from the Kamchatka Peninsula in a Greenland ice core: Implications of a widespread marker deposit that links Greenland to the Pacific northwest. *Quaternary Science Reviews* **181**, 200–206 (2018).
73. Smith, V. C. *et al.* Identification and correlation of visible tephras in the Lake Suigetsu SG06 sedimentary archive, Japan: Chronostratigraphic markers for synchronising of east Asian/west Pacific palaeoclimatic records across the last 150 ka. *Quaternary Science Reviews* **67**, 121–137 (2013).
74. Ponomareva, V. *et al.* A full holocene tephrochronology for the Kamchatsky Peninsula region: Applications from Kamchatka to North America. *Quaternary Science Reviews* **168**, 101–122 (2017).
75. Ponomareva, V. V. *et al.* The 7600 (14C) year BP Kurile Lake caldera-forming eruption, Kamchatka, Russia: stratigraphy and field relationships. *Journal of Volcanology and Geothermal Research* **136**, 199–222 (2004).
76. Pain, C. F., Blong, R. J., McKee, C. O. & Polach, H. A. Pyroclastic deposits and eruptive sequences of Long Island. *Geological Survey of Papua New Guinea Memoir* 101–113 (1981).
77. Razzhigaeva, N. G., Matsumoto, A. & Nakagawa, M. Age, source, and distribution of Holocene tephra in the southern Kurile Islands: Evaluation of Holocene eruptive activities in the southern Kurile arc. *Quaternary International* **397**, 63–78 (2016).
78. Kishimoto, H. Tephrostratigraphy and eruption style of Mashu volcano, during the last 14,000 years, eastern Hokkaido, Japan. *Bulletin of the Volcanological Society of Japan* **54**, 15–36 (2009).
79. Egan, J., Staff, R. & Blackford, J. A high-precision age estimate of the Holocene Plinian eruption of Mount Mazama, Oregon, USA. *The Holocene* **25**, 1054–1067 (2015).
80. Larsen, J. F., Neal, C. A., Schaefer, J. R. & Nye, C. J. *Geologic Map of Okmok Volcano*. https://doi.org/10.14509/31015 (2023).
81. McConnell, J. R. *et al.* Extreme climate after massive eruption of Alaska's Okmok volcano in 43 BCE and effects on the late Roman Republic and Ptolemaic Kingdom. *Proceedings of the National Academy of Sciences of the United States of America* **117**, 15443–15449 (2020).
82. Katsui, Y. & Yamamoto, M. The 1741–1742 activity of Oshima-oshima volcano, north Japan. *Journal of the Faculty of Science at Hokkaido University* **19**, 527–536 (1981).
83. Machida, H. *et al.* Holocene explosive eruptions of Witori and Dakataua caldera volcanoes in West New Britain, Papua New Guinea. *Quaternary International* **34–36**, 65–78 (1996).

84. Newhall, C.G., Daag, A.S., Delfin, F.G., Hoblitt, R.P., McGeehin, J., Pallister, J.S., Regalado, T.M., Rubin, M., Tubianosa, B.S., Tamayo, R.A., Umbal, J. V. *Eruptive History of Mount Pinatubo. Fire and Mud: Eruptions and Lahars of Mount Pinatubo, Philippines*. (Philippine Institute of Volcanology and Seismology, Quezon City, 1996).
85. McKee, C.O., Baillie, M.G., Reimer, P. J. A revised age of AD 667-699 for the latest major eruption at Rabaul. *Bulletin of Volcanology* **77**, 65 (2015).
86. Pearson, C., Sbonias, K., Tzachili, I. & Heaton, T. J. Olive shrub buried on Therasia supports a mid-16th century BCE date for the Thera eruption. *Sci Rep* **13**, 6994 (2023).
87. Coombs, M. L., Larsen, J. F. & Neal, C. A. *Postglacial Eruptive History and Geochemistry of Semisopochnoi Volcano, Western Aleutian Islands, Alaska*. 33 https://doi.org/10.3133/sir20175150 (2018).
88. Jensen, B. J. L., Beaudoin, A. B., Clynne, M. A., Harvey, J. & Vallance, J. W. A re-examination of the three most prominent Holocene tephra deposits in western Canada: Bridge River, Mount St. Helens Yn and Mazama. *Quaternary International* **500**, 83–95 (2019).
89. Delos Reyes, P. J. *et al.* A synthesis and review of historical eruptions at Taal Volcano, Southern Luzon, Philippines. *Earth-Science Reviews* **177**, 565–588 (2018).
90. Staff, R. A. *et al.* New$^{14}$ C Determinations from Lake Suigetsu, Japan: 12,000 to 0 Cal BP. *Radiocarbon* **53**, 511–528 (2011).
91. Sevink, J., Bakels, C. C., Van Hall, R. L. & Dee, M. W. Radiocarbon dating distal tephra from the Early Bronze Age Avellino eruption (EU-5) in the coastal basins of southern Lazio (Italy): Uncertainties, results, and implications for dating distal tephra. *Quaternary Geochronology* **63**, 101154 (2021).
92. Santacroce, R. *et al.* Age and whole rock-glass compositions of proximal pyroclastics from the major explosive eruptions of Somma-Vesuvius: A review as a tool for distal tephrostratigraphy. *Journal of Volcanology and Geothermal Research* **177**, 1–18 (2008).
93. Wulf, S., Kraml, M., Brauer, A., Keller, J. & Negendank, J. F. W. Tephrochronology of the 100ka lacustrine sediment record of Lago Grande di Monticchio (southern Italy). *Quaternary International* **122**, 7–30 (2004).
94. Waythomas, C.F., Neal, C. A. Tsunami generation by pyroclastic flow during the 3500-year B.P. caldera-forming eruption of Aniakchak volcano, Alaska. *Bulletin of Volcanology* **60**, 110–124 (1998).
95. Bacon, C. R., Neal, C. A., Miller, T. P., McGimsey, R. G. & Nye, C. J. *Postglacial Eruptive History, Geochemistry, and Recent Seismicity of Aniakchak Volcano, Alaska Peninsula*. (2014) doi:http://dx.doi.org/10.3133/pp1810.
96. Paine, A. R. & Wadsworth, F. B. Large explosive eruptions may be dominated by pyroclastic flows instead of buoyant plumes: insights from a global data compilation. *J Appl. Volcanol.* **14**, 3 (2025).
97. Reimer, P. J. *et al.* The IntCal20 Northern Hemisphere Radiocarbon Age Calibration Curve (0-55 cal kBP). *Radiocarbon* **62**, 725–757 (2020).
98. Mayewski, P. A. *et al.* Holocene climate variability. *Quaternary Research* **62**, 243–255 (2004).
99. Fletcher, W. J., Sánchez Goñi, M. F., Naughton, F. & Seppä, H. Synthesis and perspectives: drivers, rhythms, and spatial patterns of Holocene climate change. in *European Glacial Landscapes* 127–146 (Elsevier, 2024). doi:10.1016/B978-0-323-99712-6.00026-X.
100. Crucifix, M., Loutre, M. F. & Berger, A. The Climate Response to the Astronomical Forcing. *Space Sci Rev* **125**, 213–226 (2007).
101. Schneider, T., Bischoff, T. & Haug, G. H. Migrations and dynamics of the intertropical convergence zone. *Nature* **513**, 45–53 (2014).
102. Wang, Y. *et al.* The Holocene Asian monsoon: Links to solar changes and North Atlantic climate. *Science* **308**, 854–857 (2005).
103. Wanner, H. & Bütikofer, J. Holocene bond cycles: Real or imaginary? *Geografie-Sbornik* **113**, 338–350 (2008).
104. Thiéblemont, R., Matthes, K., Omrani, N.-E., Kodera, K. & Hansen, F. Solar forcing synchronizes decadal North Atlantic climate variability. *Nat Commun* **6**, 8268 (2015).
105. Svensmark, H., Enghoff, M. B., Shaviv, N. J. & Svensmark, J. Increased ionization supports growth of aerosols into cloud condensation nuclei. *Nat Commun* **8**, 2199 (2017).
106. Emile-Geay, J., Cane, M., Seager, R., Kaplan, A., Almasi, P. El Niño as a mediator of the solar influence on climate. *Paleoceanography* **22**, PA3210 (2007).
107. Shindell, D. T., Schmidt, G. A., Miller, R. L. & Rind, D. Northern hemisphere winter climate response to greenhouse gas, ozone, solar, and volcanic forcing. *J. Geophys. Res.* **106**, 7193–7210 (2001).
108. Swingedouw, D. *et al.* Natural forcing of climate during the last millennium: fingerprint of solar variability: Low frequency solar forcing and NAO. *Clim Dyn* **36**, 1349–1364 (2011).
109. Sha, L. *et al.* Solar forcing as an important trigger for West Greenland sea-ice variability over the last millennium. *Quaternary Science Reviews* **131**, 148–156 (2016).
110. Azoulay, A., Schmidt, H. & Timmreck, C. The Arctic Polar Vortex Response to Volcanic Forcing of Different Strengths. *JGR Atmospheres* **126**, e2020JD034450 (2021).
111. Baroni, M., Bard, E., Petit, J.-R., Magand, O. & Bourlès, D. Volcanic and solar activity, and atmospheric circulation influences on cosmogenic 10Be fallout at Vostok and Concordia (Antarctica) over the last 60years. *Geochimica et Cosmochimica Acta* **75**, 7132–7145 (2011).
112. Jordan, C. E., Dibb, J. E. & Finkel, R. C. 10Be/7Be tracer of atmospheric transport and stratosphere-troposphere exchange. *J. Geophys. Res.* **108**, 2002JD002395 (2003).
113. Brook, E. J. & Buizert, C. Antarctic and global climate history viewed from ice cores. *Nature* **558**, 200–208 (2018).
114. Lambeck, K., Rouby, H., Purcell, A., Sun, Y. & Sambridge, M. Sea level and global ice volumes from the Last Glacial Maximum to the Holocene. *Proc. Natl. Acad. Sci. U.S.A.* **111**, 15296–15303 (2014).

115. Sun, Y. *et al.* Persistent orbital influence on millennial climate variability through the Pleistocene. *Nature Geoscience* **14**, 812–818 (2021).
116. Vettoretti, G., Ditlevsen, P., Jochum, M. & Rasmussen, S. O. Atmospheric CO2 control of spontaneous millennial-scale ice age climate oscillations. *Nature Geoscience* https://doi.org/10.1038/s41561-022-00920-7 (2022) doi:10.1038/s41561-022-00920-7.
117. Zhang, X., Lohmann, G., Knorr, G. & Purcell, C. Abrupt glacial climate shifts controlled by ice sheet changes. *Nature* **512**, 290–294 (2014).
118. Zanchettin, D. *et al.* Background conditions influence the decadal climate response to strong volcanic eruptions. *Journal of Geophysical Research - Atmospheres* **118**, 4090–4106 (2013).
