# Supplementary material for: Evidence for volcanic forcing of Holocene cold events
Source: Nat Commun. 2026 May 28;17:6950. doi: 10.1038/s41467-026-73492-4 (PMC13388704; doi:10.1038/s41467-026-73492-4)
Supplement: Supplementary file 2 — Transparent Peer Review file [file 41467_2026_73492_MOESM2_ESM.pdf]

nature portfolio

Peer Review File

Evidence For Volcanic Forcing of Holocene Cold Events

Corresponding Author: Dr Alice Paine

This file contains all reviewer reports in order by version, followed by all author rebuttals in order by version.

Version 0:

Reviewer comments:

Reviewer #1

(Remarks to the Author)
Summary

The authors explore the potential of long-term volcano-climate interactions during the entire Holocene. They claim this has only been done for the Common Era but they now extend this approach across the entire Holocene. By synthesizing data from terrestrial eruption deposits, ice cores, paleoclimate archives, and glacial reconstructions, they evaluate the temporal match between major eruptions and abrupt cooling events. They then use these results to assess if a statistical link exists between explosive volcanism and centennial-scale cooling events across the NH.

General
Overall, this is a nicely written short manuscript extending similar and previous CE work back through the Holocene. This is, however, definitely not the first time researchers point to the possible link between large volcanic eruptions (atmospheric sulphur loading) and centennial-scale cooling during the entire Holocene although here their approach applying systematic integration of geological and chemostratigraphic data is kind of novel. The results are interesting although the methods are fairly simple, just applying statistics to an existing albeit limited datasets. Although I do have some concerns regarding both the data set, the limitations and the overall methodology I think it is worthy of being published in Nature Communication after the authors address some of my concerns listed below.

Major
My first concern relates to the authors' pre-defined criteria for eruption magnitude and location (60°N to 20°S). As they themselves indicate, they applied a conservative magnitude filter of ≥5.9 based mainly on volume estimates and related magnitude calculations. It is surprising that they do not place more emphasis on the climatic impact of volcanic eruptions based on the estimated sulphur production. Therefore, limiting their data to explosive volcanic eruptions of magnitudes >5.9 between latitudes 60°N and 20°S needs to be better justified. This limitation excludes, for example, all Icelandic eruptions, both explosive and effusive, even though they are known for their long lasting and high sulphur production, as explained in further detail below. Despite of this, the authors do include two Icelandic eruptions (both located north of lat 60°N - Table S1 and Figure S1): the Eldgjá eruption (937CE) and the Saksunarvatn eruption (using an outdated name and date of 10200 yr for the G10 ka eruption series that took place from ~9900-10400 BP). It is also a bit strange here that they do cite the paper by Óladóttir et al 2020 that describes the G10 ka eruption series and corrects the age range, but still do not rely on that age constraint.

Along the same line, they have a conclusion chapter focusing particularly on the 8200 yr cold event emphasizing the volcanic eruptions as a trigger and pointing at two or three possible candidates (the Kurile Lake, Tao-Rusyr, and unknown ~8.62 ka eruptions) that are all likely to have injected massive quantities of sulphur into the stratosphere. This is not the first time that volcanic eruptions have been mentioned as a possible trigger for centennial scale cooling during the Holocene – and the 8200 year cold event. A number of paleoclimatic studies in Iceland have pointed at large Icelandic eruptions as possible candidates, among them the Thjorsa lava eruption ~8600 years ago, which produced the largest lava flow on Earth in one eruption during the Holocene, possibly lasting for decades, with aerosol loading of ~340 mega tons (cf. Thordarson et al 2003; Geirsdóttir et al 2012, 2020). The 8200 year event was a particularly interesting cold event since it took place during the Holocene Thermal Maximum and thus very likely that the event was caused by a combined effect from perturbation to North Atlantic Ocean due to freshwater input and a volcanic trigger to result in the large magnitude of cooling. Here, more discussion on the role of variable NH summer insolation between the Early and Late Holocene is needed.

Another concern relates to the time offset between moraine formation and the forcing that cause the advance in a time of overall deglaciation. Certainly ice sheet – ocean interactions through the earliest Holocene are the dominant drivers. However, after 6 ka or so, ice sheets are irrelevant for cold events. This makes their correlation to glacial advances weak. Most age determinations of ice sheet advances on land, even during the 8200 years event, are dating the advance not the trigger – and even 8200 year cosmogenic dates date the moraine stabilization, which is long after the initiation of the event. This time offset between moraine formation and the forcing that caused the advance in a time of overall deglaciation needs a much more thorough discussion – and maybe some considerations that separate ice sheet advances versus local glacier advances. The reliability/lag of Late Holocene glacier advance constrains also needs to be addressed in the paper.

Finally, the authors propose mechanisms at the end for sustaining cold excursions. They could rely more on empirical reconstructions here to test if these are feasible within our known understanding of paleoclimate. As written it sounds very hypothetical but there are tons of data to explore these mechanisms. For instance, sea ice is far less prevalent during the Early and Middle Holocene than later during the Little Ice Age, again, probably related to the diminishing NH insolation during the late Holocene. So I'm not sure if it can always be proposed as a sulphur amplifier, particularly during the Early Holocene. Looking at and comparing to sea ice records would be a nice addition.

Minor

Line 34- 35: Here it would perhaps be more correct to say that on millennial timescales, the climate of the Holocene in the Northern Hemisphere was strongly influenced by the steadily decreasing summer insolation, whereas on decadal to centennial timescales Holocene climate was variable, fluctuating between warmer and colder, and more humid and more arid states (e.g. Wanner et al., 2011). I think Boreal summer insolation as the first order trend affected the whole of the Holocene evenly whether strong (Early Holocene) or weak (Late Holocene)…
Line 38: It is a bit weird starting with Bond Events, that I don't think have anything to do with volcanism
Line 56-60: I think this would be considered a circular argument…
Line 101-103: By synthesizing data from terrestrial eruption deposits, ice cores, paleoclimate archives, and glacial reconstructions, we evaluate the temporal match between major eruptions and abrupt cooling events. We then use these results to assess if a robust statistical link between explosive volcanism and centennial-scale cooling events across the NH exists.
This is perhaps the weakest part of the study, e.g. the paleoclimate database and the lag time between triggers and stable moraines. Statistical methods based on a weak database are difficult to interpret…
Line 110-111: How do the authors estimate the initiation of the glacial advance? How do they estimate the lag time between glacial advances and nearest preceding eruption date?
Line 118: What is the average frequency of large volcanic eruptions throughout the Holocene?
Line 129-130: First, only four out of 22 glacial advances do not occur within uncertainty of a known volcanic eruption (Fig. 2). This needs better clarification regarding what is considered the onset of glacier advance.
Line 173-174: Here it would be good to compare this with reconstructed sea ice data. Also note that this is more evident during the Late Holocene at times when NH summer insolation was at its minimum.

References

T. Thordarson, S. Self, D. J. Miller, G. Larsen and E. G.Vilmundardóttir 2003. Sulphur release from flood lava eruptions in the Veidivötn, Grímsvötn and Katla volcanic systems, Iceland. Geological Society, London, Special Publications p103-121. doi: 10.1144/GSL.SP.2003.213.01.07

Geirsdóttir, Á., Miller, G.H., Larsen, D., Ólafsdóttir, S., Thordarson, Th. 2003. Holocene Climate Extremes: The Little Ice Age And The 8.2 Ka Event As Viewed From Iceland. AGU Chapman Conference on
Volcanism and the Atmosphere. Selfoss, Iceland 10 – 15 June 2012

Geirsdóttir et al 2020. Holocene history of landscape instability in Iceland: Can we deconvolve the impacts of climate, volcanism and human activity? Quaternary Science Reviews 249 (2020) 106633

Reviewer #2

(Remarks to the Author)
The paper "Evidence For Volcanic Forcing of Holocene Cold Events" by Paine et al provides evidence for explosive volcanism initiation of centennial scale cold periods during the Holocene. The Introduction is well structured and lays out the possible mechanisms for explosive volcanism having both short term and centennial scale impacts on the climate. The Results provide a robust statistical analysis showing that the timing of large explosive volcanic eruptions immediately proceeding many of the Holocene's centennial scale cold periods is highly unlikely to be through random chance. The Discussion puts the results in context and describes a mechanistic framework for the long term impact of explosive volcanism on climate.

It has been my pleasure to review this manuscript. It is very well written, with clear and concise arguments and sufficient evidence to back the assertions. I believe that this work will advance the knowledge around the role of volcanism in climate variability, and will be well cited in the future. It is my recommendation that the paper be accepted for publication with minor revisions.

Below are some suggestions for some minor additions to the manuscript to improve the clarity and strength of the arguments, as well as some typographical edits.

Minor Additions

1) Typically the dating of ice cores involves matching to other palaeo-records, including sulphur/sulphate records. Therefore, the ice core dating may not be independent of the existing volcanic dating. The manuscript would benefit from a short acknowledgement (even in the Supp Info) of this potential circularity in the dating of volcanic events.

2) My understanding is that you compare RMS for E_A with N_e=11 to RMS for E_R with N_e=1 (with the latter calculated 10 million times). It might be a more appropriate (or at least insightful additional) comparison to generate 1 million ensembles of 11 randomly distributed eruptions, and calculate RMS for these alternative E_R's with N_e=11.

Typographical edits (Lxxx refers to line xxx in the pdf)
L48 "inform our ability to model", "inform" doesn't quite seem the right word here, suggest "improve" or "enhance"
L54 "so a volcanic driver for Holocene climate change is under-researched" suggest changing to "leading to difficulties in researching the impact of volcanic eruptions on Holocene climate change and variability."
L55 delete "volcanic"
L79 This is the first time you have used "M" in the main text, define as eruption Magnitude based on erupted mass
L94 This is where you could add some text (or a reference to Supp Info) around possible circularity in ice core/volcanic dating
L101 Add "CE" after "1950"
L109 Change "core" to "centre" to avoid confusion with ice cores and/or sediment cores
L116 Lines 114-116 don't quite read correctly to me. I think the easiest fix is to add a full stop after "determined"
L121 Change "eruptions precede" to "eruptions immediately precede" or similar. As written, the eruptions could precede by any (large) number of years
Figure 1 Add in coastlines to help the reader with geographic context
Figure 1,2 and 5 The captions all refer to "section" in the manuscript, but the manuscript doesn't have numbered sections. Change these references
L128 Move the citation "10" from "temperature" to "Greenland"
Figure 2 It would be useful to also show age uncertainties on this figure
Figure 3 Panel e, it is difficult to tell apart the two lines, suggest more distinct colours
L162 Add "the" between "of" and "Santorini"
L193 Sea ice also acts as a physical barrier, inhibiting exchange of heat, moisture and momentum. It would be worth mentioning this
L196 This is the first time you have used "AMOC", define it
L202 You just said at line 195 that there is strengthening of the sub-polar gyre. Need to discuss this "spin down" in context of the strengthening
Figure 4 Panel C. Two edits here. 1) swap the vertical position of the two arrows on the right hand side of the "N-S temperature gradient box" so that the two lines don't cross. 2) The line that branches into three from the right hand side of the "Increased melting at the ice-ocean boundary" is confusing. Does it a) represent a duplication of the upper and lower arrows from the right hand side, or b) an interaction between the "Reduced ocean stratification" and "Weakened convection" boxes. If so then swap the "Increased ocean fresh-to-saline water ratio" box with either the box above or below, and reposition the arrows so they don't cross
L237 This is the first time you have used "LIS", define it here rather than at line 251
L292 Missing a "S" after the degree symbol
L293 Missing a "M" before the greater than or equal to symbol
L473 Strange symbols after the dates in the article title
L601 Missing accent on "a" and extra "A" with a caret over it in "Eldgja"

Supplementary Info
L44 I think "greater" should be "smaller"
L70 Should the ">" be a "<"?
L74 Delete the ")"
L82 Change "so given that" to "because"
Figure S1 The uncertainties for the cases that were non explicitly given are not symmetric around zero. If this is in fact the case, need to explain why
L131 Add a "S" after the degree symbol
L133 Add a "M" before the greater than or equal to symbol
L135-136 It is implicit that M is quantified to one decimal point, so that M=5.9 gets all eruptions where 5.9 <= M < 6. This needs to be make explicit
Table S1 The InterCal20 ages for the two Ksudach eruptions appear to have been swapped
L191 The argument that the solar variability is only a small fraction of the change in TSI and is therefore not a driver of climate variability is weak, given that on line 35 of the Supp Info you have the Maunder Minimum change at -0.2 M/m^2
L361 Strange characters after the dates in the article title

Regards

Jason Roberts

Version 1:

Reviewer comments:

Reviewer #1

(Remarks to the Author)
I have read the article with regard to the peer review of both reviewers and believe that the authors have worked conscientiously to refine the article and thereby meet the peer reviewers. The manuscript is very well written and the authors have greatly improved the clarity and strength of arguments and both detailed the methodology and clarified the statistics as requested. The interpretation of results and conclusions are supported by the data while at the same time the authors are well aware of the limitations of the study. I am convinced that this paper will increase interests in the role of volcanism when comes to explaining climate perturbations and recommend that the paper be accepted for publication.

My only comment at this stage is a minor editing type of a comment:

L100 either skip (ka) where it is at the end of the sentence or move it to after kiloyears (ka):
"presented herein as kiloyears (ka) before 1950 CE."

...and just some food for thoughts:
L305 in regard to the discussion that starts in L305 I think you can easily refer to Figure 3e as an example of stepwise cooling from 8.6 to 8.2 ka seen in the proxy record (MD99-2269 Quartz (%)). Another possible example also based on proxy data could be Larsen et al 2012 QSR 39 who identified two distinct and severe cold pulses between 8.7 and 8.0 and suggested additional trigger mechanisms, such as increased volcanism.

Reviewer #2

(Remarks to the Author)
The revised manuscript "Evidence For Volcanic Forcing of Holocene Cold Events" by Paine et al provides evidence for explosive volcanism initiation of centennial scale cold periods during the Holocene. The authors have diligently and thoughtfully addressed the reviewer comments, resulting in a clearer manuscript worthy of publication once some minor issues are addressed to the Editor's satisfaction.

The most significant remaining issue is inconsistency between the main text and Table S1. Specifically:
- The text list n=11 for M>=7 (lines 126 and 429) but Table S1 only had 10 M>=7 eruptions, I think the problem is that Kuwae has been deleted from the table. If n=10 for M>=7 is correct, the statistical analysis for n=11 need to be redone for n=10
- The text lists a total of 50 (line 107, 367 and 372) eruptions, but (even without Kuwae) Table S1 lists 51 eruptions
- The text lists n=18 for M>6.7 (line 144), but Table S1 only has 14 (or 15 if Kuwae is added) M>6.7
- The test lists Kurile Lake as M7.3 and Tao-Rusyr as M7.1 (line 262), but Table S1 lists them as 7.2 and 7.0 respectively

Minor suggested topographical edits are listed below
- line 121 did you mean 'one year to a few decades'? i.e. add "year"
- Figure 3 caption, bold "(e)"
- line 280, you have already defined AMOC previously, so could change "Atlantic Meridional Overturning Circulation (AMOC)" to "AMOC"

I would like to finish by thanking the authors and editor for making reviewing this manuscript an enjoyable experience.

Regards

Jason Roberts

Open Access This Peer Review File is licensed under a Creative Commons Attribution 4.0 International License, which permits use, sharing, adaptation, distribution and reproduction in any medium or format, as long as you give appropriate credit to the original author(s) and the source, provide a link to the Creative Commons license, and indicate if changes were made.
In cases where reviewers are anonymous, credit should be given to 'Anonymous Referee' and the source.
The images or other third party material in this Peer Review File are included in the article's Creative Commons license, unless indicated otherwise in a credit line to the material. If material is not included in the article's Creative Commons license and your intended use is not permitted by statutory regulation or exceeds the permitted use, you will need to obtain permission directly from the copyright holder.
To view a copy of this license, visit https://creativecommons.org/licenses/by/4.0/

Please find below our detailed responses (in blue) to comments provided by Reviewers #1 and #2 The original reviewer comments are repeated here in black for clarity and completeness.

Reviewer #1

The authors explore the potential of long-term volcano-climate interactions during the entire Holocene. They claim this has only been done for the Common Era but they now extend this approach across the entire Holocene. By synthesizing data from terrestrial eruption deposits, ice cores, paleoclimate archives, and glacial reconstructions, they evaluate the temporal match between major eruptions and abrupt cooling events. They then use these results to assess if a statistical link exists between explosive volcanism and centennial-scale cooling events across the NH.

Overall, this is a nicely written short manuscript extending similar and previous CE work back through the Holocene.

> We thank Reviewer #1 for taking the time to review our manuscript, and are grateful for their positive feedback. Their comments have been really useful for improving the manuscript.

This is, however, definitely not the first time researchers point to the possible link between large volcanic eruptions (atmospheric sulphur loading) and centennial-scale cooling during the entire Holocene although here their approach applying systematic integration of geological and chemostratigraphic data is kind of novel.

> We are pleased that Reviewer #1 considers our work to be novel, and also concur that a growing number of studies have sought to quantify, and test, links between volcanism and centennial scale climate perturbations. It is exactly this increase in interest that prompted the writing of this manuscript – to synthesize the wide range of data now available and identify how these can be integrated to better identify intervals, scenarios, and/or events in which the effects of volcanic forcing could have been amplified during the Holocene.

The results are interesting although the methods are fairly simple, just applying statistics to an existing albeit limited datasets. Although I do have some concerns regarding both the data set, the limitations and the overall methodology I think it is worthy of being published in Nature Communication after the authors address some of my concerns listed below.

> We are very happy to hear that Reviewer #1 believes our manuscript is worthy of publication in *Nature Communications*, and agree that the manuscript can be improved further. In our response below, we outline how we address all comments and suggestions and the resulting adaptations to our revised manuscript.

Major

My first concern relates to the authors' pre-defined criteria for eruption magnitude and location (60°N to 20°S). As they themselves indicate, they applied a conservative magnitude filter of ≥5.9 based mainly on volume estimates and related magnitude calculations. It is surprising that they do not place more emphasis on the climatic impact of volcanic eruptions based on the estimated sulphur production. Therefore, limiting their data to explosive volcanic eruptions of magnitudes >5.9 between latitudes 60°N and 20°S needs to be better justified.

> Reviewer #1 is right to point out that sulphur emission is a crucial parameter to consider when evaluating the climate forcing potential of an explosive eruption. We also agree that more explicit clarification for why eruptions are chosen by magnitude (rather than other parameters, such as sulphur output) is needed in the manuscript.
>
> The decision to filter eruptions by magnitude (volumetric mass) rather than sulphur output (SO_2) for our statistical analysis was made for four reasons:

1) There is no single technique ideally suited, nor universally used, for quantifying pre- and syn-eruptive sulphur emissions of past eruptions. For eruptions that occurred in the pre-

instrumental era, sulphur emissions may be estimated using ice-core based reconstructions[1,2], or by petrological analysis of melt inclusions trapped in volcanic rocks[3,4]. However, evidence exists for a clear discrepancy between predicted and measured sulphur emissions; a mismatch termed the "sulphur excess problem"[5], and that suggests that placing quantitative constraints on an eruption's estimated sulphur emission would be sensitive to methodology. Not only does this introduce additional uncertainty, but it would also place greater emphasis on the subjective choice of technique.

2) Because our analysis spans both pre-instrumental and modern eruptions, selecting events based on known SO_2 would have significantly reduced the sample size and introduced a temporal bias toward well-observed, recent eruptions, because robust, quantitative estimates for sulphur emission are not available for all known Holocene eruptions. For example, due to: (a) poor exposure and/or access to eruptive materials suitable for petrological analysis, (b) no conclusive evidence (e.g., tephra) to attribute the eruption to an ice core sulphur signal. A key case illustrating this point in our manuscript is the Tao Rusyr eruption (8.31 ± 0.14 ka), for which no studies have yet attempted to quantify sulphur emissions, and no tephra has yet been found in ice cores from Greenland that would link this eruption to a definitive SO_4 peak. Both mean that if we were to filter eruptions based solely on sulphur emission, this event would be eliminated; despite being known as one of the largest explosive eruptions of the Holocene based on geological evidence.

3) Our objective in this stage of the analysis was simply to isolate the largest known Holocene eruptions; rather than to directly quantify radiative forcing. As Reviewer #1 rightly points out above, there are several studies already published that do this, and these are clearly cited in our manuscript. With this in mind, and acknowledging the fact that erupted mass and SO_2 yield is not one-to-one[3], eruption magnitude remains an effective filter criterion as it provides a practical, consistent, and reproducible proxy for eruption scale[6], and subsequently climate forcing **potential**, that can be used for comparative purposes.

4) The eruption dataset based on estimated sulphur injected into the atmosphere assumes that sulphur is the only chemical species emitted by volcanism that could affect climate. For example, we know that halogens exert a considerable effect on stratospheric ozone concentrations, and that this has knock on effects for the climate[7–9]. Although these are currently only partially understood, it is possible that halogens (or indeed other elements) are important in determining the climate response. What our approach effectively does is statistically link eruption to climate change, without assuming that it is the sulphur alone that produces the largest impacts.

We justify this decision more explicitly in our manuscript, by adding the following edits:

> **Lines 359-361**: "*The decision to filter eruptions by magnitude was made because this parameter provides a practical, consistent, and reproducible proxy for eruption scale[108], and subsequently climate forcing potential (**Fig. S3, Text S3**).*"
>
> **Lines 131-134**: "*This approach also avoids issues of circularity that could arise if we were to test the timing of volcanic eruptions relative to cooling event signals in the ice cores; given that older sections of existing ice core records are chronologically constrained using volcanic tephra layers as time-stratigraphic markers[45].*"

We have also added further context to the points made above in our supplementary information file. For example, in section **S3: Paleo-volcanic records**:

> **Lines 141-150:** "*The information provided by ice cores has been invaluable for the study of volcano-climate interactions during the Holocene[27,39]…However, there are several degrees of uncertainty associated with records of eruption-specific sulphur emissions on longer (10^3-yr) timescales. These uncertainties underpin our decision to not define the 'largest' Holocene eruption events using peaks in SAOD and/or VSSI (proxy for SO_2 output). Thus, while acknowledging the fact that erupted mass and sulphur yield is not one-to-one[6], eruption*

magnitude provides a practical, consistent, and reproducible proxy for eruption scale[44], and subsequently climate forcing potential, that can be used for comparative purposes."

Lines 168-170: *"The second reason we define eruption size by volumetric mass, rather than sulphur output, is because selecting events based on the latter would risk introducing two key issues to our analysis: (1) temporal bias, and (2) data non-uniformity."*

Here, we also describe the results of a series of additional statistical tests of the time-association between the largest VSSI and SAOD peaks (or 'eruptions'), and the global glacial advances defined by Solomina et al.

Lines 190-202: "*This analysis yielded a non-significant result, and we posit that this is most likely due to the fact that the glacial advance onsets defined by Solomina et al.[25] extend more-or-less uniformly across the entire Holocene, similar to the eruption record defined by volumetric mass (**Fig. S1**). Whereas, the largest SAOD and VSSI peaks are restricted to the first half of the Holocene (**Fig. S2**), resulting in a temporal mismatch that directly affects the statistical power of the test. For example, 'synthetic' eruptions are generated under the assumption of temporal randomness across the full Holocene window, producing a near-uniform distribution of events through time. However, because the largest SAOD peaks appear concentrated into the ~9–6 ka window, the Monte Carlo simulations systematically overestimate the expected temporal overlap with glacial advances under the null hypothesis: inflating the baseline coincidence level, and causing the RMS statistic to become dominated by large temporal gaps. Hence, causing glacier advance[25] onsets to better correlate with eruptions identified from geological evidence (**Table S1**), but produce a non-significant result when tested against the largest VSSI/SAOD peaks[27].*"

Finally, we include an additional figure in our supplementary file to illustrate the broadly consistent relationship between eruption magnitude and sulphur emission. Hence, the former can serve as a practical first-order proxy for eruption scale, and thus climate forcing potential, in the absence of a consistent method for accurately determining sulphur yields from past eruptions:

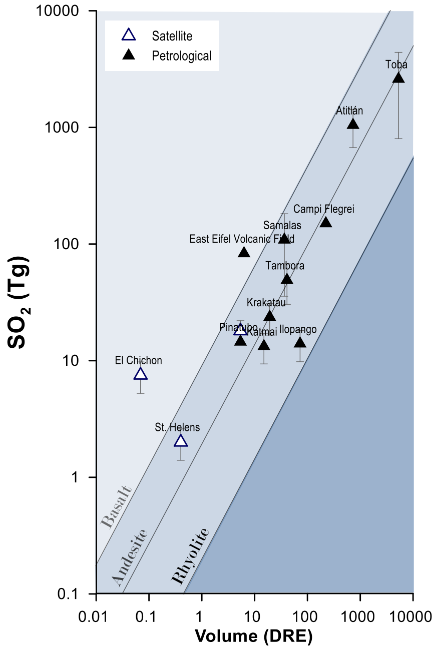

Figure S3: Erupted volume (dense-rock equivalent (DRE)) versus SO_2 flux (Tg) plotted for a selection of large volcanic eruptions known to have occurred over the past ~100-kyr. Eruptions for whom SO_2 values have been calculated using petrological methods are marked as black triangles. Direct satellite-derived values are marked by white triangles. Blue shading highlights the three primary bulk magma compositions as defined by sulphur solubility[4]

This limitation excludes, for example, all Icelandic eruptions, both explosive and effusive, even though they are known for their long lasting and high sulphur production, as explained in further detail below.

> Given that (as stated) below, our criteria still does permit inclusion of two Icelandic eruptions, we are unsure how to resolve the first component of this comment. Regarding the point about persistent and high sulphur production, we agree that there is evidence for high sulphur outputs, and measurable climate effects, following certain Icelandic eruptions. For example, the Lakí eruption of 1783-4 CE[10], and the 939 CE eruption of Eldgjá [11] are cited as the two most sulphur-rich Icelandic eruptions known to have occurred during the Holocene[12].
>
> We do not include the Lakí eruption, as this event falls short of the magnitude required for inclusion. To add this eruption to our compilation would introduce several inconsistencies into our selection framework. For example, the inclusion of effusive and/or "hybrid" eruptions. Although Lakí produced exceptionally high sulphur emissions perhaps relative to other Icelandic eruptions[12], it was predominantly an effusive fissure eruption; where volatile fluxes were spread over multiple months[13], and likely driven by highly complex (and often simultaneous) eruptive dynamics, styles, and mass eruption rates. Thus, classifying the 'true' magnitude of the eruption remains a challenge, and even more so to determine the extent to which the explosive phases of the event contributed to the overall sulphur yield. This is unlike the other eruptions included in our compilation, where geological evidence pertains to a single, cataclysmic eruption episode characterized by rapid magma discharge, sustained high eruption columns, and efficient injection of ash and gases into the stratosphere over a comparatively short time interval – an observation facilitated by the literature review conducted as part of our compilation process:
>
> > **Lines 363-365:** "*The second stage of data compilation involved a thorough intra-study comparison, where volumetric and chronological data was cross-checked with all literature currently available for each eruption individually: to determine the consistency in values…*"
>
> Therefore, altering our inclusion criterion to accommodate sulphur-rich but lower-magnitude effusive eruptions would require systematic re-assessment of all similarly complex volcanic events known for the Holocene. This would substantially alter the scope of the dataset, but also introduce uneven data quality and greater uncertainty. For example, because sulphur emissions are not consistently quantified for many pre-instrumental eruptions.
>
> As Reviewer #1 acknowledges below, we do include the 937 CE eruption of Eldgjá in our compilation, as sufficient evidence exists to suggest this event was predominantly explosive in nature, and so consistent with our stated methodological approach

Despite of this, the authors do include two Icelandic eruptions (both located north of lat 60°N - Table S1 and Figure S1): the Eldgjá eruption (937CE) and the Saksunarvatn eruption (using an outdated name and date of 10200 yr for the G10 ka eruption series that took place from ~9900-10400 BP). It is also a bit strange here that they do cite the paper by Óladóttir et al 2020 that describes the G10 ka eruption series and corrects the age range, but still do not rely on that age constraint.

> We are grateful to Reviewer #1 for pointing out the discrepancy in our latitude criteria, and eruptions included in our compilation. We have now corrected the stated latitude range:
>
> > **Line 347**: "*…criteria for location (90ºN to 20ºS)…*"
>
> It is true that Óladóttir et al. (2020) presents evidence to suggest that the high magnitude attributed to the Saksunarvatn/G10 ka series is actually a product of multiple eruptions over a multi-year period, rather than a single event. Also, that no single radiocarbon age has been ascribed to the eruption event/series/cluster, based on terrestrial evidence. Our inclusion of the Saksunarvatn/G10 ka series here is justified by evidence for an exceptionally large massive sulphate spike (>200 kg km^2), corresponding to a geochemically-distinct visible tephra layer in multiple ice cores from Greenland (GRIP, NGRIP, GISP2):
>
> GRIP (1528.59m) – Grönvold et al. (1995) *Earth & Planetary Science Letters*

GISP2 (1586.95) – Zielinski et al. (1997) *Journal of Geophysical Research*

NGRIP (1409.82m) – Mortensen (2005) *Journal of Quaternary Science*

When placed on the Common Era GICC05 timescale by Vinther et al. (2006), this signal corresponds to an age of 10.3±0.90 ka. More recently, it was placed onto the WD2014 timescale by Sigl et al. (2022) and so ascribed an updated age of 10.23±20, which is also consistent with the IntCal13/GICC05 age transfer presented by Adolphi & Muscheler (2016).

If this layer (shown in **Figure 5a** in Sigl et al., 2022) represents only one episode out of a series of eruptions related to the Saksunarvatn/G10 ka series, the presence of this sulphur signal still implies that the event had the potential to cause a measurable climate impact.

Here, we assume that Reviewer #1's view that our previous age listing for the Saksunarvatn/G10 ka series was "outdated" on the basis that it attempted to place too much weight on the ^{14}C data. So, to minimize any associated confusion, we amend the manuscript and **Table S1** to focus solely on the phase captured next to large quantities of sulfuric acids in the NGRIP, GRIP, NEEM and GISP2 ice cores and ascribed an age of 10.23±0.02 ka (Sigl et al. *2022)*.

Volcano	Latitude	Eruption Unit	M	^{14}C Age	Ref	IntCal20 Age	Ice Core Age	Ref
Grímsvötn	64.42	Saksunarvatn	6.6	-	-	-	10180±20	[1]

Along the same line, they have a conclusion chapter focusing particularly on the 8200 yr cold event emphasizing the volcanic eruptions as a trigger and pointing at two or three possible candidates (the Kurile Lake, Tao-Rusyr, and unknown ~8.62 ka eruptions) that are all likely to have injected massive quantities of sulphur into the stratosphere. This is not the first time that volcanic eruptions have been mentioned as a possible trigger for centennial scale cooling during the Holocene – and the 8200 year cold event. A number of paleoclimatic studies in Iceland have pointed at large Icelandic eruptions as possible candidates, among them the Thjorsa lava eruption ~8600 years ago, which produced the largest lava flow on Earth in one eruption during the Holocene, possibly lasting for decades, with aerosol loading of ~340 mega tons (cf. Thordarson et al 2003; Geirsdóttir et al 2012, 2020).

These are certainly important publications to consider, and we thank Reviewer #1 for bringing them to our attention. Given that similar to Laki, the Thjórsá eruption was predominantly (if not entirely) effusive in style[12], we do not add this event to our compilation. However, in light of the environmental significance of this eruption rightly pointed out by Reviewer #1, we add a reference to this event and its potential influence on the 8.2 ka event:

> **Lines 267-269:** "*Although there are no eruptions in our compilation clearly aligned to this peak, one possibility is that it is linked to the effusive Þjórsá eruption of the Bárðarbunga volcanic system in southern Iceland at ~8.6 ka – the largest Holocene lava flow known to have been emplaced during a single event*[14,15]."

Furthermore, we also add a series of additional clauses to our earlier discussion…

> **Lines 210-214**: "*The radiative effects arising from a sulphur injection into the atmosphere by a large explosive eruption(s) in the northern hemisphere would first cause pronounced surface cooling*[16]*. Such a cooling effect also arises if we consider the magnitude-frequency relationship*[17]*, which highlights that high-frequency 'clusters' of smaller explosive, and/or particularly sulphur-rich effusive eruptions may also deliver substantial quantities of sulphur to the lower stratosphere, capable of producing a potent radiative effect*[20,21]*.*"

…and to the section of the manuscript focusing on the 8.2-ka event specifically:

> **Lines 302-305:** "*Therefore, the occurrence of an ~8.6 ka precursor explosive, sulphur-rich eruption could reconcile existing proxy-model discrepancies that cast uncertainty over if, and how, early cooling signals relate to the peak ~8.2 ka event*[20]*.*"

The 8200 year event was a particularly interesting cold event since it took place during the Holocene Thermal Maximum and thus very likely that the event was caused by a combined effect from perturbation to North Atlantic Ocean due to freshwater input and a volcanic trigger to result in the large magnitude of cooling.

The 8.2 ka event is certainly an interesting Holocene climate excursion, given that it took place under a series of unique boundary conditions. We agree with Reviewer #1 that these conditions warrant closer inspection relative to our framework in this manuscript. To do this, we add the following paragraph:

Lines 275-285*: "During the early Holocene, freshwater inputs to the North Atlantic would already be high due to the opening of a new drainage pathway into the Hudson Strait, and accelerated melt of the remnant Laurentide Ice Sheet (LIS) during the final stages of deglaciation*[21–23]*. Furthermore, numerous marine and terrestrial proxy records also suggest gradually increasing temperatures across the northern hemisphere during this time, associated with major readjustment of regional temperature gradients, progressive strengthening of the Atlantic Meridional Overturning Circulation (AMOC)*[24]*, and progressive shifts in solar activity*[24,25]*. Together, these records strongly suggest that an "intermediate" climate state existed prior to the 8.2-ka event characterized by moderate-to-low sea-ice extent*[26]*, high variability in solar output*[25]*, declining insolation*[24]*, elevated meltwater inputs to the North Atlantic*[21]*, and large-scale atmospheric reorganisation*[27]*. Hence, a climate system that would have been highly sensitive to external forcing relative to the mid- and late-Holocene*[26,28]*."*

Here, more discussion on the role of variable NH summer insolation between the Early and Late Holocene is needed.

To add further discussion on the role of solar variability/insolation within the 8.2-ka event framework, we first highlight existing evidence for heightened solar and freshwater variability during this interval:

Lines 277-284*: "Furthermore, numerous marine and terrestrial proxy records also suggest gradually increasing temperatures across the northern hemisphere during this time, associated with major readjustment of regional temperature gradients, progressive strengthening of the Atlantic Meridional Overturning Circulation (AMOC)*[24]*, and progressive shifts in solar activity*[24,25]*. Together, these records strongly suggest that an "intermediate" climate state existed prior to the 8.2-ka event characterized by moderate-to-low sea-ice extent*[26]*, high variability in solar output*[25]*, declining insolation*[24]*, elevated meltwater inputs to the North Atlantic*[21]*, and large-scale atmospheric reorganisation*[27]*..."*

…before placing our hypothesis into this broader context:

Linea 289-291*: "We propose that their sulphur emissions perturbed a climate system already primed for change by* ***solar, cryospheric, and atmospheric*** *factors, by promoting hemispheric cooling that reinforced ocean–atmosphere feedbacks already operating near a stability threshold."*

We also place the significance of these background conditions into a broader context, relating it specifically to the late Holocene:

Lines 284-285*: "Hence, a climate system that would have been highly sensitive to* ***external forcing*** *relative to the mid- and late-Holocene*[26,28]*"*

Furthermore, we include two sections in our supplementary information file that speaks directly to the importance of background conditions in the context of the Holocene climate system, both of which are directly applicable to the 8.2 ka event:

S4: Orbital (solar) forcing

S5: Role of background conditions

Another concern relates to the time offset between moraine formation and the forcing that cause the advance in a time of overall deglaciation. Certainly ice sheet – ocean interactions through the earliest

Holocene are the dominant drivers. However, after 6 ka or so, ice sheets are irrelevant for cold events. This makes their correlation to glacial advances weak.

Ice sheets certainly have a diminishing importance throughout the Holocene, and would not have directly forced all of the cold events during the Holocene. However, we do not directly relate, or correlate, ice sheet dynamics to glacial advances, and so assume that Reviewer #1 is referring to the fact we discuss the Laurentide ice sheet, and its importance for freshwater fluxes in the interval preceding the 8.2 ka event (which is also associated with a glacial advance). We seek to clarify this is an event-specific reference by adding the following paragraph:

Lines 275-285*: "During the early Holocene, freshwater inputs to the North Atlantic would already be high due to the opening of a new drainage pathway into the Hudson Strait, and accelerated melt of the Laurentide Ice Sheet (LIS) during the deglaciation*[21–23]*. Furthermore, numerous marine and terrestrial proxy records also suggest gradually increasing temperatures across the northern hemisphere during this time, associated with major readjustment of regional temperature gradients, progressive strengthening of the Atlantic Meridional Overturning Circulation (AMOC)*[24]*, and progressive shifts in solar activity*[24,25]*. Together, these records strongly suggest that an "intermediate" climate state existed prior to the 8.2-ka event characterized by moderate-to-low sea-ice extent*[26]*, high variability in solar output*[25]*, declining insolation*[24]*, elevated meltwater inputs to the North Atlantic*[21]*, and large-scale atmospheric reorganisation*[27]*. Hence, a climate system that would have been highly sensitive to external forcing relative to the mid- and late-Holocene*[26,28]*."*

Most age determinations of ice sheet advances on land, even during the 8200 years event, are dating the advance not the trigger – and even 8200 year cosmogenic dates date the moraine stabilization, which is long after the initiation of the event.

Reviewer #1 is correct that it is unlikely that the event onset was at precisely 8.2 ka, and indeed this is a point we already make explicitly in the manuscript:

Lines 271-305: "*A growing number of paleoclimate records covering this interval show that the earliest signs of cooling associated with the 8.2 ka event began between ~8.6 and 8.5 ka*[20]*, over 100 years before the date of the earliest known Laurentide outburst flood (8.47 ± 0.3 ka)*[78]*. Indeed, the peak cooling signal in the Greenland temperature reconstructions occurs at ~8.6 ka - immediately following an unusually large injection of sulphur into the atmosphere (**Fig. 5**)…Therefore, the occurrence of an ~8.6 ka precursory explosive and/or sulphur-rich eruption could reconcile existing proxy-model discrepancies that cast uncertainty over if, and how, early cooling signals relate to the peak ~8.2 ka event*[20]."

This is a point that also supports our statistical approach, as it does not require a strict "match-up" of the eruption and peak climate signal to yield an informative result.

This time offset between moraine formation and the forcing that caused the advance in a time of overall deglaciation needs a much more thorough discussion. The reliability/lag of Late Holocene glacier advance constrains also needs to be addressed in the paper.

We agree that more information related to the time offsets between moraine formation, forcing, and the reliability of the glacial advance dataset used in our study needs to be presented in the manuscript. In response to this, we add the following text to **Results**:

Lines 109-113: "*The timing of each glacial advance is determined from the compilation of radiometric ages acquired from moraines across 17 regions compiled by Solomina et al.*[30]*. By marking the point of moraine stabilization, these ages provide a near-direct indication of centennial-scale temperature and precipitation fluctuations (see **Methods**)*[31,32]."

Lines 120-129: "*In periods and in regions where annually resolved glacier records are abundant, such as in the Alps, their advances are predominantly attributed to the climate effects (and feedbacks) from clusters of major volcanic eruption with time delays as low as one to a few decades*[17,40,44]*. However, over the full Holocene and on a global scale, we cannot quantify the precise time offset between moraine formation, and the initial 'trigger' forcing because: (1) our dataset is a regional compilation of terrestrial exposure ages rather than annually resolved glacier records, and (2) the associated moraine ages are almost all synthesized from multiple*

*glaciers of different sizes, shapes and types, and located at various elevations[38]. Here, we test the time-association between the largest (M ≥7) known Holocene volcanic eruptions north of 20ºS by volume (n = 11; **Table S1**) and glacial advance datasets relative to the null hypothesis: if explosive volcanism is not causally linked to Holocene glacial advances, eruptions dated to that period would not closely precede cooling events more often than they would by chance alone."*

Further details on the input dataset(s) are also included in the later Methods section, and so we add an explicit reference to this section here:

Line 113: "*Glacial advances are used here to define the timing of abrupt cold events, as they provide a near-direct indication of centennial-scale temperature and precipitation fluctuations (see **Methods**)*[31,32]."

We make two further additions to our supplementary file section **S2: Cold event definition**. First, is a figure that clearly presents the uncertainties associated with the composite moraine ages:

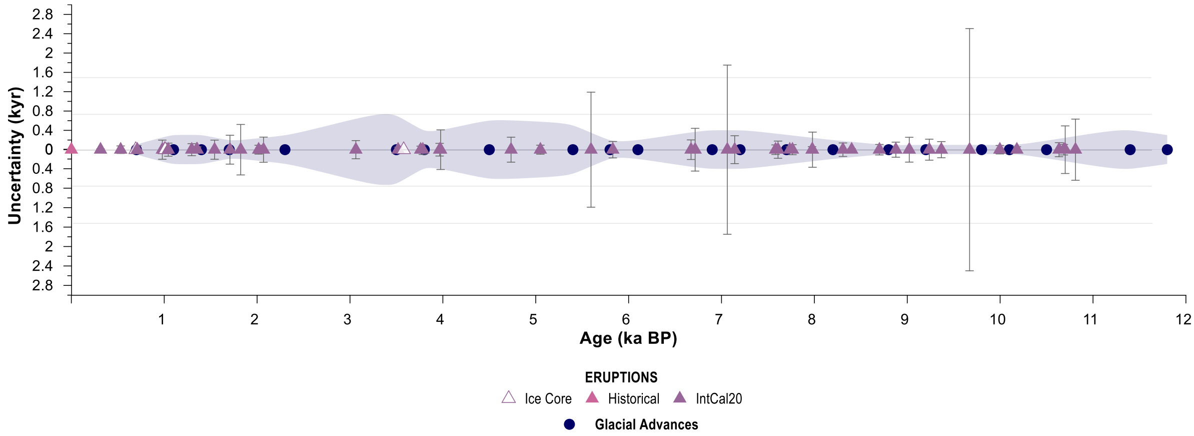

Figure S1: Uncertainties in glacial advance and eruption dates. Glacial advance dates are based on the compilation by Solomina et al.[25] , with blue shading marking upper and lower uncertainty estimates. Eruption dates derive from various sources (all listed in **Table S1**), and error bars mark the range in ages given in this study.

Second, we add three new paragraphs that explain our use of glaciological data for delineation of cold events in our study, rather than ice core, or marine sediment records:

Lines 67-73: "*In this study, we define the timing of abrupt Holocene cooling events by the onsets of major glacial advances, derived from compilation of radiometric ages acquired from moraines across 17 regions compiled by Solomina et al.[25] (**Fig. S1**). Two secondary sources of information were also considered for definition of these cold events: (1) temperature reconstructions derived from argon and nitrogen isotopes contained within air trapped inside the GISP2 ice core[23], and (2) sedimentary records of ice-rafted debris deposition in the North Atlantic[26]. Here, we outline why both of these sources were deemed unsuitable for our intended purpose…*"

Finally, we provide details for all glacial advances (including minimum and maximum ages, and spatial distribution) in an accompanying supplementary data file, which will be made openly accessible to readers.

Finally, the authors propose mechanisms at the end for sustaining cold excursions. They could rely more on empirical reconstructions here to test if these are feasible within our known understanding of paleoclimate. As written it sounds very hypothetical but there are tons of data to explore these mechanisms. For instance, sea ice is far less prevalent during the Early and Middle Holocene than later during the Little Ice Age, again, probably related to the diminishing NH insolation during the late Holocene. So I'm not sure if it can always be proposed as a sulphur amplifier, particularly during the Early Holocene.

We agree that there is a huge body of research that could be cited within our discussion. However, we are conscious that the current format for articles submitted to Nature Communications has a 70-reference limit. Further, it was not our intention to propose that sea ice is a direct “amplifier” of sulphur, and so Reviewer #1 is right to express uncertainty if this is the interpretation reached. Specifically as it relates to the early Holocene, where proxy records indeed show that sea ice was notably lower (e.g., Maffezzoli et al. 2021; Zhang et al. 2025). Taken together, we interpret this as indication that more clarity is required in our explanations of two components: (a) the role of volcanism relative to other (low-amplitude) forcing, and (b) how sulphur injection into the atmosphere translates to a response in the surface ocean and cryosphere.

Corresponding text amendments in direct response to (a) and (b) have been made as follows:

(a) The role of volcanism relative to other (low-amplitude) forcing.

> **Lines 201-206***: “If these dynamical, long-term sulphate aerosol effects are considered relative to the role of long-term, low amplitude climate forcing factors (e.g., variations in insolation*[33]*), explosive volcanism may represent the driver that prompted the climate to cool rapidly, and recurrently, at discrete points throughout the Holocene. Therefore, our work underscores the need to view volcanic perturbations not merely as short-lived radiative events, but as important catalysts – capable of initiating complex feedbacks within the coupled ocean–atmosphere system*[34]*.”*
>
> **Lines 245-250: “***Hence, they represent the culmination of the ocean–atmosphere feedbacks initiated by volcanic aerosol forcing, and superimposed onto the background state set by lower-amplitude forcing factors (e.g., insolation changes)*[33]*. In this framework, volcanism acts as a pacing and intensifying mechanism, triggering nonlinear responses within the climate system that emerge from the interaction between episodic sulphur emission events, and other underlying, more gradual radiative fluctuations*.”
>
> **Lines 289-291**: “*We propose that their sulphur emissions perturbed a climate system already primed for change by solar, cryospheric, and atmospheric factors, by promoting hemispheric cooling that reinforced ocean–atmosphere feedbacks already operating near a stability threshold....”*

(b) How sulphur injection into the atmosphere translates to a response in the surface ocean and cryosphere.

> **Lines 210-211**: “*The radiative effects arising from a sulphur injection into the atmosphere by a single – or a series of – large explosive eruption(s) in the northern hemisphere would* ***first*** *cause pronounced surface cooling*[16].”
>
> **Lines 220-221**: “*The second involves a rapid expansion of sea ice in the North Atlantic, in response to the volcanic-aerosol-driven surface cooling (****Fig. 4b****).”*
>
> **Lines 230-231: “***On centennial timescales, two processes amplify the role of sea ice dynamics (and their associated feedbacks) – relative to the initial radiative response***.**”
>
> **Lines 291-293**: “*For example, sulphur emissions produced by the first eruption at ~8.62 ka could have caused a transient expansion of sea ice in response to the surface cooling, prompting a decrease in surface heat loss (****Fig. 4c****)..*”

Also inspired by this comment from Reviewer #1, we make two further changes. First, we integrate references to the following papers in our discussion:

> Cabedo-Sanz et al. (2016) Variability in drift ice export from the Arctic Ocean to the North Icelandic Shelf over the last 8000 years: A multi-proxy evaluation. *Quaternary Science Reviews* **273**: 107249 → A comprehensive review of sea ice reconstructions from the North Atlantic covering the interval 8–0 ka.
>
> Maffezzoli et al. (2021) Sea ice in the northern North Atlantic through the Holocene: Evidence from ice cores and marine sediment records. *Quaternary Science Reviews* **273**: 107249 → Builds on the review of Cabedo-Sanz et al. (2016) to provide a multi-proxy overview of sea ice changes throughout the Holocene. Source of the RECAP ice core record added to **Figure 3** (see below)

Zhang et al. (2025) Enhanced Arctic sea-ice retreat due to pronounced pacificization effect in the Holocene. *Communications Earth & Environment* **6**: 834 → A recent review of existing proxy evidence for sea ice advance/retreat in the Arctic covering the interval 8–0 ka. Although this paper does not cover the earliest Holocene, it provides important context for assessing the mid- to late-Holocene variations also presented in our work.

Moros et al. (2006) Holocene history of drift ice in the northern North Atlantic: Evidence for different spatial and temporal modes. *Paleoceanography* **21**: PA2017 → A full-Holocene record of drift ice dynamics offshore Iceland, derived from marine sediment core MD99-2269. This record has also been added to **Figure 3** (see below).

Second, we add additional details to our description of sea ice's role within our framework (with citations):

Lines 221-226: "*Several studies have shown that both alpine glacier[44,62] and sea ice[27,28,53] extent are both highly sensitive to volcanic forcing, but also that the extent and characteristics of sea ice in the North Atlantic region underwent a series of abrupt changes throughout the Holocene (**Fig. 3e**)[55,68]. Sea ice reflects incoming solar radiation, while also serving as a physical barrier that inhibits exchange of heat and moisture between the ocean and atmosphere[53]. Thus, expansion of sea ice extent alters atmosphere-ocean heat exchanges, stratification, and deep-water formation sites in the Arctic and Nordic Seas[69,70].*"

Lines 230-231: "*On centennial timescales, two processes amplify the role of sea ice dynamics (and their associated feedbacks) – relative to the initial radiative response…*"

Lines 287-293: "*The Kurile Lake, Tao-Rusyr, and unknown ~8.62 ka eruptions are all likely to have injected massive quantities of sulphur into the stratosphere, consistent with ice core records of volcanism and regional cooling from Greenland (**Fig. 5**). We propose that their sulphur emissions perturbed a climate system already primed for change by solar, cryospheric, and atmospheric factors, by promoting hemispheric cooling that reinforced ocean–atmosphere feedbacks already operating near a stability threshold. For example, sulphur emissions produced by the first eruption at ~8.62 ka could have caused a transient expansion of sea ice in response to the surface cooling, prompting a decrease in surface heat loss (**Fig. 4c**).*"

Looking at and comparing to sea ice records would be a nice addition.

This is a good point, and we agree that addition of sea ice records would provide important context for our discussion. To this end, we have added two records to **Figure 3**:

1) Sodium (Na) concentrations in the RECAP ice core[26]
2) Quartz abundance (%) in sediment core MD99-2269 from offshore Iceland[40,43]

These records were chosen on the basis of the fact that both cover the near-full Holocene, and provide high-resolution proxies of sea ice variability from terrestrial (1) and marine (2) settings, respectively.

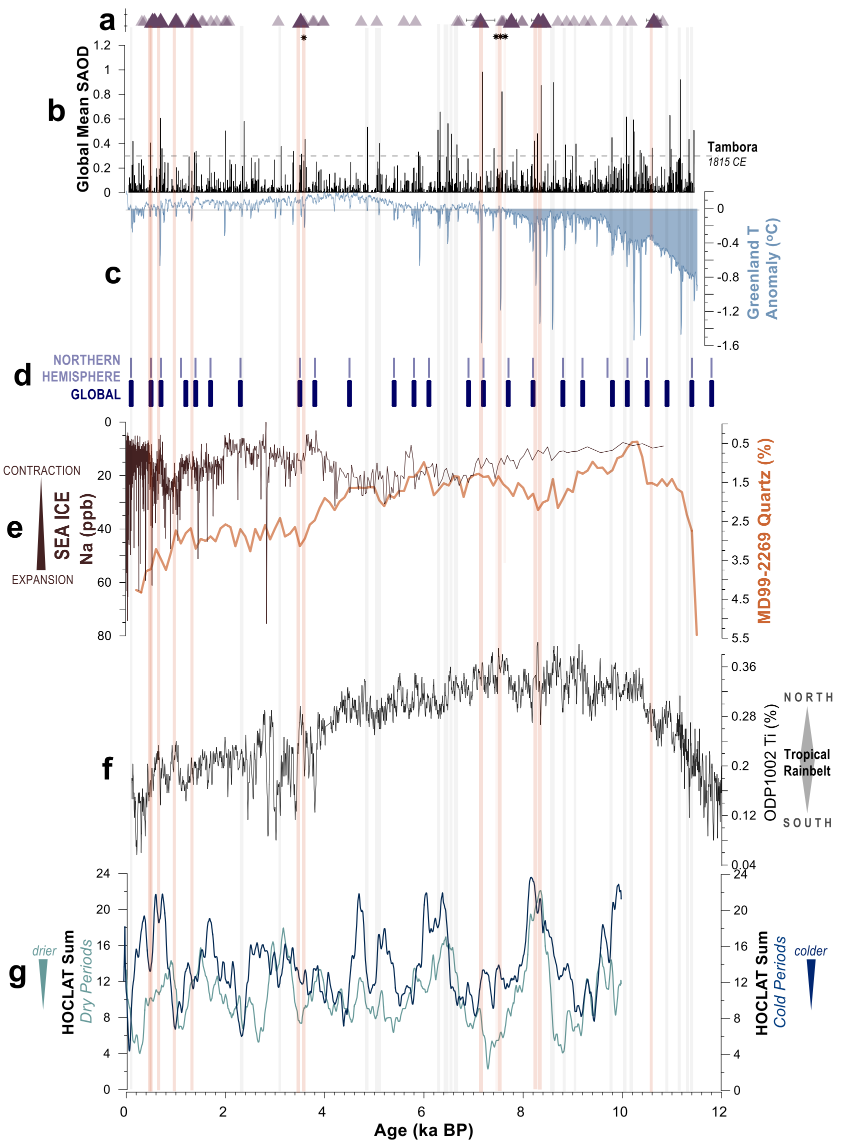

Figure 3: A comparison of key datasets mentioned within the text, listed from top-to-bottom: **(a)** explosive volcanic eruptions compiled as part of this study (see **Methods**); **(b)** ice-core derived estimates of stratospheric aerosol optical depth (SAOD) from the HolVol reconstruction, presented on the WD2014 timescale and with a dashed horizontal line marking estimated SAOD values produced by Tambora (1815 CE)[1,2]**; (c)** reconstructed Greenland temperature anomalies over the Holocene derived from argon and nitrogen isotopes[44]; **(d)** onset dates for major northern hemisphere (dark blue) and southern hemisphere (light blue) glacial advances[30]; **(e) records of sea ice variability derived from the RECAP ice core (Greenland[26], and sediment succession MD99-2269 (offshore Iceland)**[43]; **(f)** a record of elemental titanium (Ti) in the ODP 1002 sediment core (Cariaco Basin, Venezuela) smoothed by a three-point running mean, where higher Ti concentrations indicate more terrestrial runoff as evidence for a farther-northward summer ITCZ position[45]; **(g)** weighted curves representing the sum of cold and dry periods within the HOCLAT paleoclimate data compilation[46]. Key eruption events are labelled, and asterisks (*) mark events for whom tephra deposits have been identified in Greenland[47]. Orange bars mark the largest 10 eruptions in our compilation that occur <100 years prior to a glacier advance event. Grey bars mark large SAOD peaks with no known eruption source, but also occur <100 years prior to a glacier advance event.

Minor

Line 34- 35: Here it would perhaps be more correct to say that on millennial timescales, the climate of the Holocene in the Northern Hemisphere was strongly influenced by the steadily decreasing summer insolation, whereas on decadal to centennial timescales Holocene climate was variable, fluctuating between warmer and colder, and more humid and more arid states (e.g. Wanner et al., 2011). I think Boreal summer insolation as the first order trend affected the whole of the Holocene evenly whether strong (Early Holocene) or weak (Late Holocene)…

This is a good suggestion, and we agree that clearer distinction is needed between low- and high-amplitude signals that have characterized the Holocene climate system. Thus, this section has been amended to now read:

Lines 33-38: "*Boreal summer insolation modulated key changes in climate and atmospheric circulation during the early Holocene, with a progressively increasing influence from anthropogenic effects related to deforestation, agriculture, and industrialisation[1]. On decadal-to-centennial timescales, these low-amplitude trends were also punctuated by a series of repeated, aperiodic, century-scale cold 'snaps' characterised by pronounced surface cooling, an altered hydrological cycle, and ecological disruption[2,3].*"

Line 38: It is a bit weird starting with Bond Events, that I don't think have anything to do with volcanism

We agree that referring to Bond Events seems odd here, and may add unnecessary confusion. This reference has been removed, and the text amended as follows:

Lines 38-39: "*First identified in the North Atlantic as distinct peaks in Icelandic volcanic glass and/or hematite-stained grains, these cold events were initially linked to southward advection of icebergs…*"

Line 56-60: I think this would be considered a circular argument…

Fair point, the way this statement is worded can be improved so to ensure the point is not circular. We have now made this clarification:

Lines 54-60: "*A key development was the generation of ice-core sulphate-based reconstructions of volcanism across the Holocene[9] which, combined with tree-ring and historical records, now reveal compelling temporal coincidences between annual-to-decadal-scale temperature variations, major explosive eruptions, and key cultural transitions, upheavals, and hardships[11]. By proposing a causal link between volcanic eruptions and climate disturbance, these coincidences highlight a clear need for a deeper investigation into how long volcanic-induced climate impacts can last, and the utility of paleoclimate data and historical eruption records for this purpose.*"

Line 101-103: This is perhaps the weakest part of the study, e.g. the paleoclimate database and the lag time between triggers and stable moraines. Statistical methods based on a weak database are difficult to interpret…

There are certainly non-trivial uncertainties in use of moraine exposure ages to date glacial advances, and Reviewer #1 is right to highlight that these points need to be more clearly conveyed in the manuscript. We do this by making three key changes:

1) We now highlight that this is the reason our conclusions are not reliant solely on the results of the statistical analysis. Rather, the analysis permitted an initial test of whether volcanic eruptions were a **candidate** trigger for Holocene cold events. Specifically, by testing whether the largest known eruptions precede glacial advances more closely in time than they would by chance. To clarify this point, we amend the following sentence:

Lines 126-129: "*… we test the time-association between our eruption and glacial advance datasets relative to the null hypothesis that, if explosive volcanism is not causally linked to Holocene glacial advances, eruptions dated to that period would not closely precede cooling events more often than they would by chance alone.*"

2) We more clearly define the association between glacial advances and changes in mass balance:

Lines 109-118: "*The timing of each glacial advance is determined from the compilation of radiometric ages acquired from moraines across 17 regions compiled by Solomina et al.[38]. By marking the point of moraine stabilization, these ages record the culmination of sustained positive glacier mass balance, and therefore provide a near-direct indication of centennial-scale temperature and precipitation fluctuations (see **Methods**)[39,40]. Sustained intervals in which accumulation exceeds ablation lead to glacier thickening and increased ice flux to the terminus, producing advances after a dynamic response lag of years to decades[41]. For example, periods of accelerated glacier growth typically occur when conditions are cold enough to sustain ice throughout the year[42]. Therefore, alpine glaciers are much more sensitive to centennial-scale climate shifts than large ice sheets, which more clearly capture millennial-scale glacial-interglacial oscillations[43].*"

3) We more explicitly integrate existing lines of evidence that suggest not only that glaciers are sufficiently sensitive to volcanic forcing to respond with a measurable effect, but also that the lag time between the eruptive trigger and glacier mass balance increase/moraine stabilization would have been short (<100 years). For example:

- Glacier mass-balance model experiments show significant increases in ice volume for up to 5 years after being subject to the forcing observed following eruptions of Agung (Indonesia; 1963 CE), Pinatubo (the Philippines; 1991 CE), and El Chichón (Mexico; 1982 CE)[35]. These same experiments also show a significant increase in the magnitude of volcanic forcing required to stimulate glacial mass balance changes during the late Holocene (1850 CE to present), due to increasing influence from anthropogenic factors (e.g., greenhouse gas emissions)[35].

  Lines 221-222: "*Several studies have shown that both* ***alpine glacier***[35,36] *and sea ice*[37–39] *extent are both highly sensitive to volcanic forcing…"*

- Over the past ~800 years, volcanic eruptions are shown to have triggered glacier advances the European Alps with lag times equating to less than 10 years[32,36,53].

  Lines 198-200: *"Combined with evidence for rapid (<10-20-year) sea ice and* ***glacial expansion*** *followed by prolonged ocean circulation weakening, and interhemispheric climate reorganization following major eruptions (**Fig. 3**)*[18,19,35,37,38,53]*, these findings…"*

- Quantitative calculations suggest that glaciers can react to a shift in climatic conditions on the order of annual-to-decadal timescales, depending on the topography of the surrounding landscape[50].

  Lines 113-115: "*Sustained intervals in which accumulation exceeds ablation lead to glacier thickening and increased ice flux to the terminus, producing advances after a dynamic response lag of years to decades*[41]*…*"

As Reviewer #1 rightly points out, our compilation shows that we clearly cannot expect degrees of temporal precision comparable to the Common Era on a global scale, nor throughout the entire Holocene. However, if we consider glacial advances on a scale that encompasses the pre-industrial (arguably more sensitive) climate system, larger eruptions, and/or multiple regions, volcanic forcing provides a plausible and dynamically consistent mechanism for initiating glacier expansion, and so this suggests that volcano–climate interactions have played a significant role in modulating glacier behaviour during the Holocene.

Line 110-111: How do the authors estimate the initiation of the glacial advance?

We agree more clarity is needed here, and so add the following details:

Lines 109-118: "*The timing of each glacial advance is determined from the compilation of radiometric ages acquired from moraines across 17 regions compiled by Solomina et al.*[38]*. By marking the point of moraine stabilization, these ages record the culmination of sustained positive glacier mass balance, and therefore provide a near-direct indication of centennial-scale temperature and precipitation fluctuations (see* ***Methods****)*[39,40]*. Sustained intervals in which accumulation exceeds ablation lead to glacier thickening and increased ice flux to the terminus, producing advances after a dynamic response lag of years to decades*[41]*. For example, periods of accelerated glacier growth typically occur when conditions are cold enough to sustain ice throughout the year*[42]*.*"

How do they estimate the lag time between glacial advances and nearest preceding eruption date?

To estimate this lag time, we identified the nearest eruption to each discrete glacial advance and calculated the difference in their ages. These time offsets were then squared, averaged across all event pairs, and then square-rooted to yield a single RMS value. We have now added the acquired RMS value to the main text:

> **Lines 136-138**: "*We first captured the lag time between a given glacial advance and the nearest eruption date, and then calculated a root-mean-square (RMS) statistic from all the event pairs, where the RMS statistic is a function of N_e(the number of eruptions), T_k^e (the eruption dates), and T_j^c (the glacial advance dates) …*"

Important to note is that it is not possible to give precise (quantitative) time offsets, given that the glacial advance ages represent a collective value based on a large array of geochronological data. To ensure this point is clear in the manuscript, we add the following text to the **Results** section:

> **Lines 120-131:** *"In periods and in regions where annually resolved glacier records are abundant, such as in the Alps, their advances are predominantly attributed to the climate effects (and feedbacks) from clusters of major volcanic eruption with time delays as low as one to a few decades[17,40,44]. However, over the full Holocene and on a global scale, we cannot quantify the precise time offset between moraine formation, and the initial 'trigger' forcing because: (1) our dataset is a regional compilation of terrestrial exposure ages rather than annually resolved glacier records, and (2) the associated moraine ages are almost all synthesized from multiple glaciers of different sizes, shapes and types, and located at various elevations[38]. Here, we test the time-association between the largest (M ≥7) known Holocene volcanic eruptions north of 20ºS by volume (n = 11**; Table S1**) and glacial advance datasets relative to the null hypothesis: if explosive volcanism is not causally linked to Holocene glacial advances, eruptions dated to that period would not closely precede cooling events more often than they would by chance alone. This negates the need for a precise 'match' between the eruption and moraine exposure ages, and so allows us to account for non-linear processes that could prolong the effects of the initial volcanic cooling."*

Line 118: What is the average frequency of large volcanic eruptions throughout the Holocene?

On timescales of <10^6 years, large explosive volcanic eruptions are random and independent events in time. It is true that a general global magnitude-frequency relationship can be reconstructed from, and expressed as, a return interval between two discrete eruptions of a given magnitude, based on historical and geological observations[54]. For example, Sigl et al. (2022) used sulphur peaks in Greenland and Antarctic ice cores to derive an estimated frequency of one eruption every ~14 years for eruptions emitting >1 Tg sulphur[1] – equivalent to the magnitude (M) 4.0 eruptions of Nabro (2011 CE) and Kasatochi (2008 CE). This frequency reduces with magnitude, such that one M≥8 eruption is expected to occur every ~17,000 years[55,56] – an estimate supported by mathematical modelling[54].

We make explicit reference to eruption frequency and its relation to our approach in section **Methods: Statistical Analysis**. However, inspired by Reviewer #1's question (and point above about prior work in this domain), we also add references to key literature that also discuss the frequency of large explosive eruptions:

> **Lines 430-437**: *"Here, we consider the largest (M≥7) eruptions within our compilation (n=11). This equates to ~one eruption per kiloyear, which is a frequency consistent with statistical modelling[57]. This may result in omission of climatologically significant eruptions; however, using too many eruptions would artificially inflate the likelihood of a false-positive result where no actual link between an eruption and the climate response exists. Our method increases the likelihood that any identified link between an eruption that passed through this filtering and a glacial advance is meaningful, but also that some glacial advances were caused by eruptions that were filtered out. Hence, our null hypothesis posited that known eruptions precede glacial advances no more closely in time than they would by chance alone."*

Although long-term average recurrence intervals provide a useful contextual baseline for how often eruptions of different magnitudes typically occur, they do not imply a strict temporal uniformity or event distribution. Volcanism can cluster in time due to tectonic, magmatic, or mantle-dynamic processes, and regional eruption rates may deviate from global means over millennial timescales. Therefore, applying a generalized global return interval to our analysis would not be appropriate.

Line 129-130: First, only four out of 22 glacial advances do not occur within uncertainty of a known volcanic eruption (Fig. 2). This needs better clarification regarding what is considered the onset of glacier advance.

> As outlined in our response above, we have sought to clarify this point by adding the following text to the manuscript:
>
> > **Lines 109-118**: "*The timing of each glacial advance is determined from the compilation of radiometric ages acquired from moraines across 17 regions compiled by Solomina et al.*[38]*. By marking the point of moraine stabilization, these ages record the culmination of sustained positive glacier mass balance, and therefore provide a near-direct indication of centennial-scale temperature and precipitation fluctuations (see **Methods**)*[39,40]*. Sustained intervals in which accumulation exceeds ablation lead to glacier thickening and increased ice flux to the terminus, producing advances after a dynamic response lag of years to decades*[41]*. For example, periods of accelerated glacier growth typically occur when conditions are cold enough to sustain ice throughout the year*[42]*.*"

Line 173-174: Here it would be good to compare this with reconstructed sea ice data. Also note that this is more evident during the Late Holocene at times when NH summer insolation was at its minimum.

> Again, we agree with Reviewer #1 that addition of sea ice records provides important context for our discussion. Such a comparison is explicitly added to **Figure 3** (see amended figure above), and we add explicit reference to this in the text:
>
> > **Line 200**: "*eruptions (**Fig. 3**)*[18,19,37,38]"

Reviewer #2

The paper "Evidence For Volcanic Forcing of Holocene Cold Events" by Paine et al provides evidence for explosive volcanism initiation of centennial scale cold periods during the Holocene. The Introduction is well structured and lays out the possible mechanisms for explosive volcanism having both short term and centennial scale impacts on the climate. The Results provide a robust statistical analysis showing that the timing of large explosive volcanic eruptions immediately proceeding many of the Holocene's centennial scale cold periods is highly unlikely to be through random chance. The Discussion puts the results in context and describes a mechanistic framework for the long term impact of explosive volcanism on climate.

It has been my pleasure to review this manuscript. It is very well written, with clear and concise arguments and sufficient evidence to back the assertions. I believe that this work will advance the knowledge around the role of volcanism in climate variability, and will be well cited in the future. It is my recommendation that the paper be accepted for publication with minor revisions.

> We are very grateful to Reviewer #2 for taking the time to review our manuscript, and delighted to read their positive comments. Their comments have helped improve the manuscript considerably.

Below are some suggestions for some minor additions to the manuscript to improve the clarity and strength of the arguments, as well as some typographical edits.

> We also agree that revisions to the manuscript can be made, and so in our response below, we outline how we address all comments and suggestions and the resulting adaptations to our revised manuscript.

Minor Additions

1) Typically the dating of ice cores involves matching to other palaeo-records, including sulphur/sulphate records. Therefore, the ice core dating may not be independent of the

existing volcanic dating. The manuscript would benefit from a short acknowledgement (even in the Supp Info) of this potential circularity in the dating of volcanic events.

This is a good suggestion, and we have summarized the independence of the ice-core dating from the climate records used in this study in our **Methods** section as follows:

> **Lines 398-406**: "*The volcanic reconstruction is based on four ice cores, all synchronized to the annual-layer counted WAIS Divide (WD)2014 chronology from Antarctica. This timescale used the following eruptions as age constraints: Tambora (Indonesia), Huaynaputina (Peru), Samalas (Indonesia), and Miyake (Japan). The Greenland ice-core used in this study is additionally constrained using documentary records of volcanic dust veils in ~1.41, 1.32, and 1.01 ka as described by Stothers and Rampino[112] and Sigl et al.[9], together with the eruption markers described above. No climate information has been used for the chronologies, and no external age constraints have been used before 1.41 ka in either Greenland or Antarctica, but the WD2014 is in excellent agreement (+/- 15 years) with dendrochronological dates throughout the Holocene (e.g., ^{10}Be, ^{14}C)[9,31]*"

We have also added the following text to our supplementary file:

> **Lines 75-84**: "*The ice core-derived temperature reconstruction presented in Kobashi et al.[23] is derived from isotopic measurements of occluded air in the GISP2 ice core (Greenland). From this time series, the coldest events can be isolated by calculating residuals from a 15-point moving average, which highlighted the events defined as data with values more than 0.2 °C below the moving average (n = 28). However, despite the clear temporal agreement of this record with the VSSI/SAOD curves (**Fig. S2**)[27], the decision was made not to use this data to define the cold events in our analysis. First, because it could introduce circularity to the result if tested against (and compared with) the ice core-derived sulphur record, especially in cases where the best eruption age currently available is based on ice core data.*"

2) My understanding is that you compare RMS for E_A with N_e=11 to RMS for E_R with N_e=1 (with the latter calculated 10 million times). It might be a more appropriate (or at least insightful additional) comparison to generate 1 million ensembles of 11 randomly distributed eruptions, and calculate RMS for these alternative E_R's with N_e=11.

   We agree further clarification is needed here. We compared N_e = 11 to RMS for E_R with N_e = 11; in other words, the 1 million MC ensembles produced the same number of eruptions per simulation (n = 11) as there were actual Holocene eruptions (n = 11). We have made this clearer in the text as follows:

> **Lines 449-453**: "*The probability distribution and cumulative distribution functions of this statistic were then assessed by generating 10 million random eruption dates (E_R), with the assumption that these dates were uniformly and independently distributed across the interval of interest (~11.7–0.6 ka). As part of this, we compared N_e = 11 to the RMS for E_R with N_e = 11; meaning that the 1 million MC ensembles produced the same number of eruptions per simulation (n = 11) as there were actual Holocene eruptions as specified above (n = 11).*"

Typographical edits

L48 "inform our ability to model", "inform" doesn't quite seem the right word here, suggest "improve" or "enhance"

Agreed. This sentence has now been amended to read:

> **Lines 47-49**: "*Third, to enhance our ability to model the system's response to both natural and anthropogenic forcing under a range of future emission scenarios…*"

L54 "so a volcanic driver for Holocene climate change is under-researched" suggest changing to "leading to difficulties in researching the impact of volcanic eruptions on Holocene climate change and variability."

This is a good suggestion, and we agree one that improves the clarity of this sentence. This has now been amended:

> **Lines 53-54**: "*leading to difficulties in assessing the impact of volcanic eruptions on Holocene climate change and variability…*"

L55 delete "volcanic"

Done. Sentence now reads:

> **Lines 54-57**: *"A key development was the generation of ice-core sulphate-based reconstructions of volcanism across the Holocene[9] which, combined with tree-ring and historical records, now reveal compelling temporal coincidences between annual-to-decadal-scale temperature variations, major explosive eruptions, and key cultural transitions, upheavals, and hardships[11]."*

L79 This is the first time you have used "M" in the main text, define as eruption Magnitude based on erupted mass

This has now been clarified:

> **Line 79**: "*…very few large (magnitude (M) ≥6) eruptions…*"

L94 This is where you could add some text (or a reference to Supp Info) around possible circularity in ice core/volcanic dating

This is a good suggestion, and we agree that the use of volcanic eruptions as chronological markers in ice cores must be explicitly acknowledged. However, we also note that it does not affect the results of our analysis. This is because we do not define the timing of the glacial advances using the ice cores, and instead choose an independent source of information (e.g., moraine ages). Therefore, even if an eruption was included in ice core chronological development, that eruption is still independent of the cold events. We have added the following text to clarify this point in the manuscript:

> **Line 79**: "*This approach also avoids issues of circularity that could arise if we were to test the timing of volcanic eruptions relative to cooling event signals in the ice cores; given that older sections of existing ice core records are chronologically constrained using volcanic tephra layers as time-stratigraphic markers[44].*"

L101 Add "CE" after "1950"

Done:

> **Line 101**: "*presented herein as kiloyears before 1950 CE (ka)*."

L109 Change "core" to "centre" to avoid confusion with ice cores and/or sediment cores

We agree our previous wording choice could cause confusion, and so have made the following adjustment:

> **Lines 126-129**: "*Here, we test the time-association between the largest (M≥7) known Holocene volcanic eruptions north of 20°S by volume (n = 11**; Table S1**) and glacial advance datasets relative to the null hypothesis: if explosive volcanism is not causally linked to Holocene glacial advances, eruptions dated to that period would not closely precede cooling events more often than they would by chance alone*."

L116 Lines 114-116 don't quite read correctly to me. I think the easiest fix is to add a full stop after "determined"

Reviewer #2 is right to point out this as a typing error, which we have now corrected:

> **Lines 141-142**: "*…was significantly less than for E_R was determined. E_A was defined by dates all calibrated to the IntCal20 curve*[59]."

L121 Change "eruptions precede" to "eruptions immediately precede" or similar. As written, the eruptions could precede by any (large) number of years

We agree this statement could be more precise, and so make the following amendment:

Lines 126-129: "*… if explosive volcanism is not causally linked to Holocene glacial advances, eruptions dated to that period would not closely precede cooling events more often than they would by chance alone*."

Figure 1 Add in coastlines to help the reader with geographic context

Good suggestion, this figure has now been edited to include landmass boundaries and coastlines.

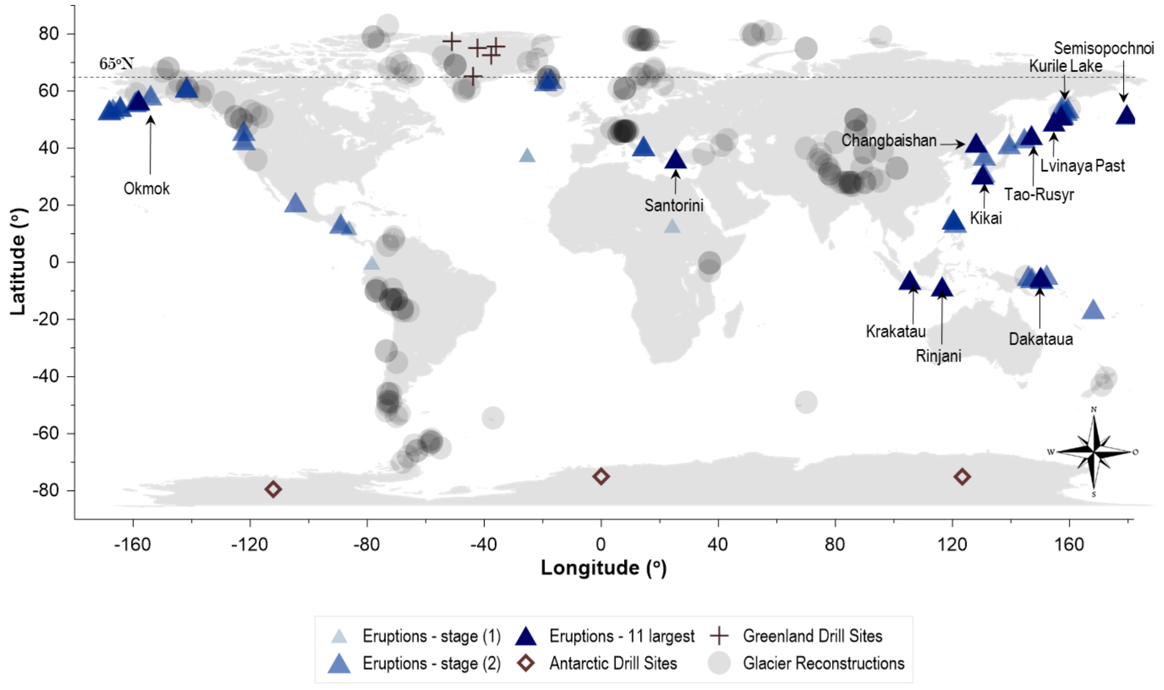

Figure 1: Locations of eruption events (blue triangles), reconstructed glacier advances (grey circles)[38], and paleoclimate reconstructions from Greenland (dark brown crosses), the North Atlantic (orange crosses), and Antarctica (brown diamonds) relevant to assessment of Holocene climate change [31,47,48]. The sources of the eleven largest (M≥7) eruptions are labelled and coloured darker blue. A dashed black line marks 65°N latitude.

Figure 1,2 and 5 The captions all refer to "section" in the manuscript, but the manuscript doesn't have numbered sections. Change these references

The following captions have now been amended:

Figure 1: Locations of eruption events (blue triangles), reconstructed glacier advances (grey circles)[38], and paleoclimate reconstructions from Greenland (dark brown crosses), the North Atlantic (orange crosses), and Antarctica (brown diamonds) relevant to assessment of Holocene climate change [31,47,48]. The sources of the eleven largest (M≥7) eruptions are labelled and coloured darker blue. A dashed black line marks 65°N latitude.

Figure 2: Temporal links between the onset of glacial advances[38], and known volcanic eruptions occurred during the Holocene. Large black circles mark coincidences (within chronological uncertainty) between glacial advances and the largest eruptions in our compilation (M≥7), and smaller grey circles represent coincidences with any of the other 31 eruptions that passed the filtering (described in **Methods**). Dashed lines represent glacial advances that do not occur within uncertainty of a known volcanic eruption meeting our predefined selection criteria. The largest eruptions are labelled by their corresponding unit name. Chronological uncertainties associated with each eruption and glacial advance in this figure are included in a **supplementary information** file.

Figure 5: Comparison of volcanic and chemostratigraphic records spanning the 8.2 ka event interval. Triangles denote the timing of the large explosive eruptions included in our compilation (**Table 1**), with dark colouring given to those large enough for inclusion in our statistical analysis, and grey horizontal lines displaying uncertainties associated with the IntCal20 calibration. Blue shading shows reconstructed Greenland temperature anomalies over the Holocene derived from argon and nitrogen isotopes[10], compared with a record of global mean stratospheric aerosol optical depth (SAOD) generated from the HolVol dataset[31]. Vertical purple shading marks an interval marked by evidence for glacial advance[38], and grey shading marks the timing of a discrete cold event within the HOCLAT proxy data compilation[3].

L128 Move the citation "10" from "temperature" to "Greenland"

Done:

Line 153: "*temperature in Greenland*[44],"

Figure 2 It would be useful to also show age uncertainties on this figure

We agree that errors should be available for the data used in this manuscript. To this end, we have added the following statement to the caption of Figure 2:

"…*Chronological uncertainties associated with each eruption and glacial advance in this figure are included in a* ***supplementary information*** *file*."

We also explored if, and how, error bars could be included in this figure in earlier stages of manuscript preparation. However, the decision was made to not include error bars in the main version because (a) errors on many of the eruption ages were small enough for the bars to be completely hidden by the eruption points, and (b) it compromised the clarity of the figure, while also being difficult to discern the error values being presented. Thus, although error bars have not been added to this figure in the revised manuscript, we have endeavoured to include clear mention of the fact this information is openly available in our supplementary information and data files.

Figure 3 Panel e, it is difficult to tell apart the two lines, suggest more distinct colours

We have now changed the colour of the "Dry Periods" curve to better distinguish between the two datasets:

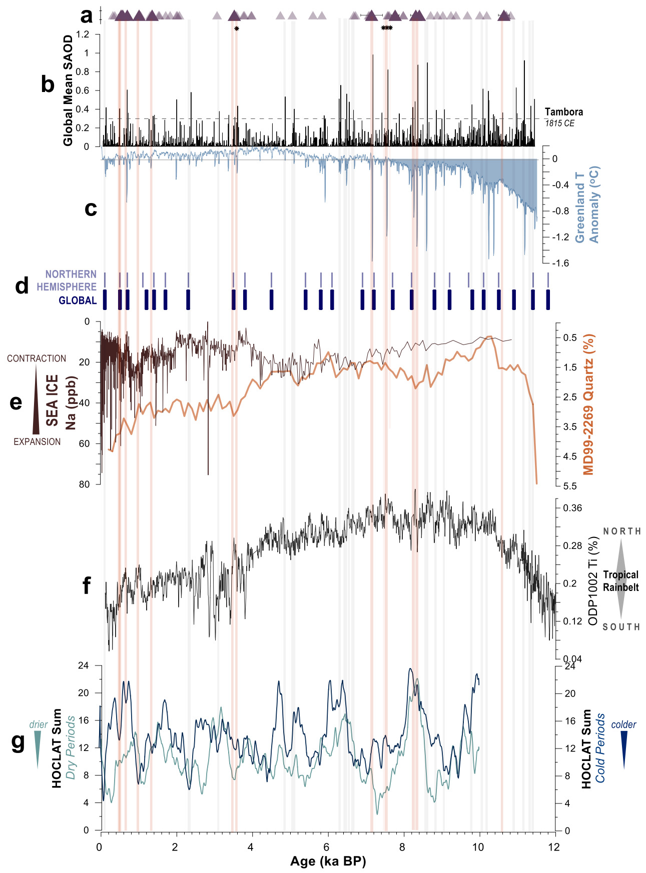

Figure 3: A comparison of key datasets mentioned within the text: **(a)** explosive volcanic eruptions compiled as part of this study (see **Methods**); **(b)** ice-core derived estimates of stratospheric aerosol optical depth (SAOD) from the HolVol reconstruction, presented on the WD2014 timescale and with a dashed horizontal line marking estimated SAOD values produced by Tambora (1815 CE)[9,31]**; (c)** reconstructed Greenland temperature anomalies over the Holocene derived from argon and nitrogen isotopes[10]; **(d)** onset dates for major northern hemisphere (dark blue) and southern hemisphere (light blue) glacial advances[38]; (e) records of sea ice variability derived from the RECAP ice core (Greenland)[55], and sediment succession MD99-2269 (offshore Iceland)[56]; **(f)** a record of elemental titanium (Ti) in the ODP 1002 sediment core (Cariaco Basin, Venezuela) smoothed by a three-point running mean, where higher Ti concentrations indicate more terrestrial runoff as evidence for a farther-northward summer ITCZ position[49]; **(g)** weighted curves representing the sum of cold and dry periods within the HOCLAT paleoclimate data compilation[3]. Key eruption events are labelled, and asterisks (*) mark events for whom tephra deposits have been identified in Greenland[34]. Orange bars mark the largest 10 eruptions in our compilation that occur <100 years prior to a glacier advance event. Grey bars mark large SAOD peaks with no known eruption source, but also occur <100 years prior to a glacier advance event.

L162 Add "the" between "of" and "Santorini"

Sentence has now been amended:

Lines 186-187: "*and may explain the notable absence of a large SAOD peak corresponding to the Minoan eruption of the Santorini volcano, Greece…*"

L193 Sea ice also acts as a physical barrier, inhibiting exchange of heat, moisture and momentum. It would be worth mentioning this

We have now added reference to this point in the text:

Lines 224-226: "*Sea ice reflects incoming solar radiation, while also serving as a physical barrier that inhibits exchange of heat and moisture between the ocean and atmosphere[47]. Thus, expansion of sea ice extent alters atmosphere-ocean heat exchanges, stratification, and deep-water formation sites in the Arctic and Nordic Seas[41,42].*"

L196 This is the first time you have used "AMOC", define it

Sentence has now been corrected:

Lines 238-239: "*…weakening of the Atlantic meridional overturning circulation (AMOC)…*"

L202 You just said at line 195 that there is strengthening of the sub-polar gyre. Need to discuss this "spin down" in context of the strengthening

We agree that this phrasing requires clarification. Thus, we have amended the passage to make more explicit reference to the fact that this spin-down effect follows the initial SPG strengthening:

Lines 234-236: "*This heat anomaly also reverses the initial strengthening of the sub-polar gyre, causing a "spin down" effect that progressively reduces ocean stratification, and advection of heat and salt*[38]."

Figure 4 Panel C. Two edits here. 1) swap the vertical position of the two arrows on the right hand side of the "N-S temperature gradient box" so that the two lines don't cross. 2) The line that branches into three from the right hand side of the "Increased melting at the ice-ocean boundary" is confusing. Does it a) represent a duplication of the upper and lower arrows from the right hand side, or b) an interaction between the "Reduced ocean stratification" and "Weakened convection" boxes. If so then swap the "Increased ocean fresh-to-saline water ratio" box with either the box above or below, and reposition the arrows so they don't cross

We thank Reviewer #2 for their suggestions on how this figure can be improved, and have now amended the flow diagram in **Figure 4c** by ensuring none of the arrows cross over each other:

Suggestion 1) Placement of the arrows branching off the "+ N-S temperature gradient" box has been amended so the upper arrow connects to "Intensification of the Polar vortex", and the lower arrow to "Lower North Atlantic SSTs".

Suggestion 2) We have swapped the position of "Weakened convection" and "Increased ocean fresh-to-saline water ratio", and edited the arrow placements accordingly.

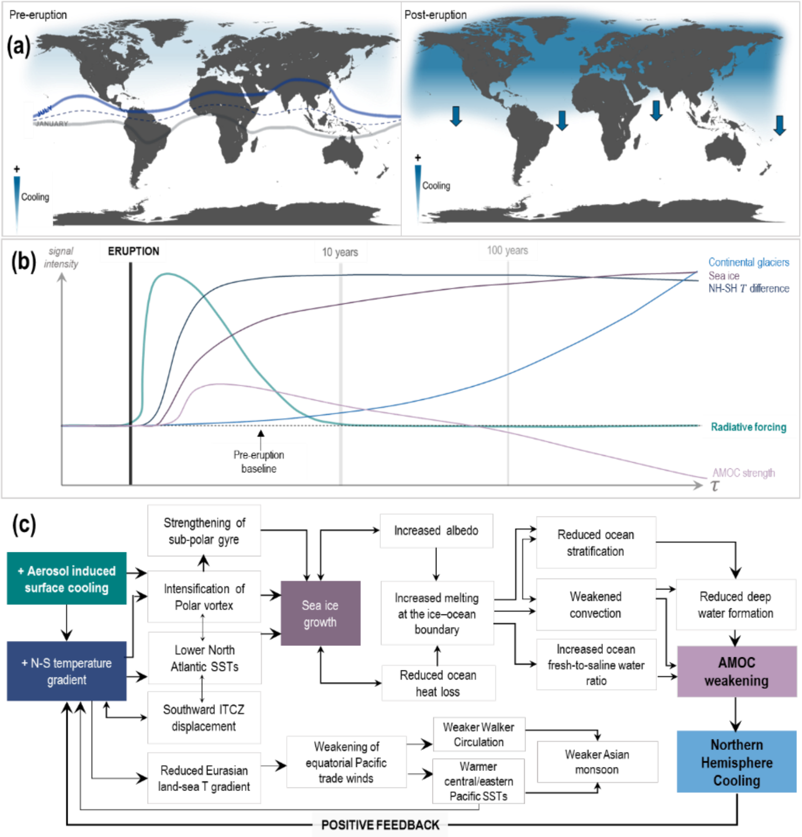

Figure 4: A framework illustrating how a large (M≥6) northern hemisphere volcanic eruption could produce a centennial-scale glacier advance. **(a)** World maps illustrating changes in the hemispheric temperature gradient following an explosive (NH) eruption, with blue shading marking the strength of cooling (darker = colder). For the pre-eruption map, longitudinal variations in mean ITCZ position based on average precipitation for January (grey) and June (blue) between 1979 and 1995 are also shown, with a dashed black line denoting the mean position (data sourced from the International Research for Climate Prediction (http://iri.ldeo.columbia.edu); adapted from Deplazes et al.[62]). **(b)** Schematic diagram showing the time (τ)- resolved evolution of climate system components known to be sensitive to volcanic forcing, and/or propagate eruption-induced feedbacks for multiple centuries. Curves are colour-coded to match their corresponding box in part **(c)**, and a dashed grey horizontal line marks the pre-eruption baseline state for each variable; such that if a curve goes above (below) this line, the associated signal is stronger (weaker) than it was under pre-eruption conditions. **(c)** A unified framework describing the sequence of climate feedbacks proposed as drivers of Holocene glacial advances.
Acronyms used include: SSTs for sea-surface temperatures, AMOC for Atlantic Meridional Overturning Circulation, and ITCZ for Intertropical Convergence Zone.

L237 This is the first time you have used "LIS", define it here rather than at line 251

This has now been adjusted:

Line 277: "*melt of the Laurentide Ice Sheet (LIS) during the deglaciation*[21–23]"

L292 Missing a "S" after the degree symbol

Now corrected:

Line 348: "*tropical (0-20ºS) southern hemisphere*."

L293 Missing a "M" before the greater than or equal to symbol

Added as follows:

Line 353: "…*magnitude filter of M≥5.9*…"

L473 Strange symbols after the dates in the article title

Well spotted, and likely an error in the reference storage software. This has now been corrected so the reference reads as:

Toohey, M. & Sigl, M. Volcanic stratospheric sulfur injections and aerosol optical depth from 500 BCE to 1900 CE. *Earth System Science Data* **9**, 809–831 (2017)

L601 Missing accent on "a" and extra "A" with a caret over it in "Eldgja"

Now corrected:

Thordarson, T., Miller, D. J., Larsen, G., Self, S. & Sigurdsson, H. New estimates of sulfur degassing and atmospheric mass-loading by the 934 AD Eldgjá eruption, Iceland. *Journal of Volcanology and Geothermal Research* (2001)

Supplementary Info

L44 I think "greater" should be "smaller"

We agree the phrasing of this sentence could be improved. To better make the point that the placement of tropical eruptions can result in the erupted gas being distributed into both hemispheres more equally (and thus the opposite of higher latitude volcanoes), we revise "greater" to now read:

Line 43: "*whereas those emitted by tropical eruptions exhibit **less pronounced** north-south partitioning*[63]."

L70 Should the ">" be a "<"?

Yes, and so this has now been corrected:

Line 92: "…*considering the short-term (*$<10^1$ *years) climate effects*…"

L74 Delete the ")"

Done.

L82 Change "so given that" to "because"

Amended to read:

Line 108: *"…and because Bond Events…"*

Figure S1 The uncertainties for the cases that were non explicitly given are not symmetric around zero. If this is in fact the case, need to explain why.

We thank the reviewer for pointing this out. On closer inspection, this discrepancy was the product of an issue with the scaling in the plotting software. The error bar size has now been corrected (figure pasted below), and to ensure full transparency, we include a supplementary file with our submission that contains all glacial advance dates, and their respective uncertainties.

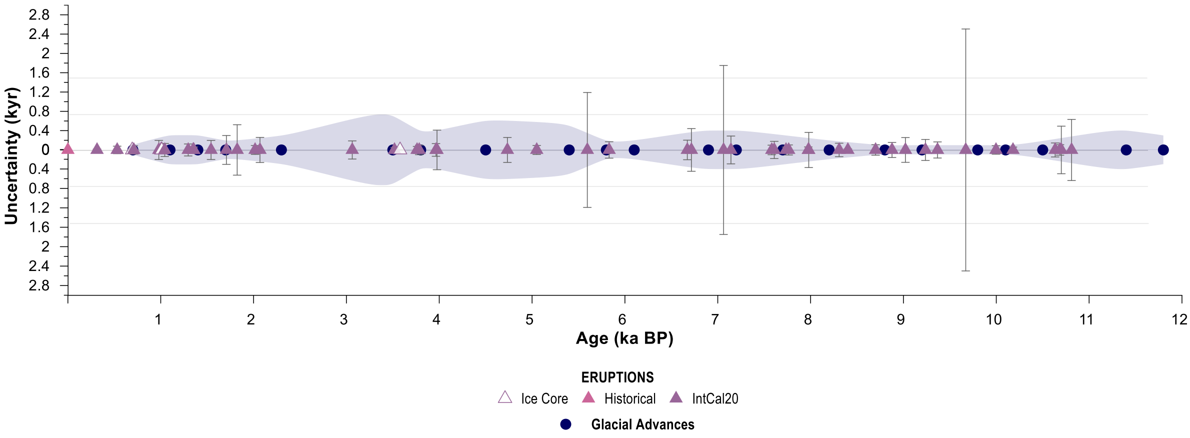

Figure S1: Uncertainties in glacial advance and eruption dates. Glacial advance dates are based on the compilation by Solomina et al.[25] , with blue shading marking upper and lower uncertainty estimates. Eruption dates derive from various sources (all listed in **Table S1**), and error bars mark the range in ages given in this study.

L131 Add a "S" after the degree symbol

Done:

Line 348: *"tropical (0-20°S) southern hemisphere eruptions…"*

L133 Add a "M" before the greater than or equal to symbol

Done:

Line 114: *"…Few large (magnitude (M)≥6) eruptions have been directly observed …"*

L135-136 It is implicit that M is quantified to one decimal point, so that M=5.9 gets all eruptions where 5.9 <= M < 6. This needs to be make explicit.

This should certainly be made more clear, and this has now been added to the main manuscript:

Lines 354-358: *"A conservative magnitude filter of M≥5.9 was initially applied to our search, where M is quantified to one decimal point. Thus, eruptions listed as M=5.9 includes all eruptions with M values between 5.9 and 6.0. Eruptions with magnitudes listed as 5.9 were independently assessed in order to verify volumetric estimations and their associated magnitude calculations."*

Table S1 The InterCal20 ages for the two Ksudach eruptions appear to have been swapped

Great spot. This has now been corrected (pasted below), and all other eruptions in **Table S1** checked to ensure no similar errors had arisen.

Ksudach	51.80	KS1	6.3	**1850±240**	64,65	**1824±523**	-	
Ksudach	51.80	KS2	5.9	**7133±195**	64,65	**7980±364**	7089±26	47

L191 The argument that the solar variability is only a small fraction of the change in TSI and is therefore not a driver of climate variability is weak, given that on line 35 of the Supp Info you have the Maunder Minimum change at -0.2 M/m^2

This is a fair point, and we agree our phrasing in the original submission was too simplistic in the context of this topic; where further work is undoubtedly needed. To respect this complexity, we have removed this statement so that the text now reads:

Lines 230-233: "*However, what remains unclear is if, and how, solar variability could explain the more abrupt atmospheric and terrestrial signals associated with Holocene cold events*[66]*. First, because the mechanisms that dictate the sensitivity of the Earth's climate to solar variability also remain poorly understood… Another source of uncertainty in attribution…*"

L361 Strange characters after the dates in the article title

Now corrected:

Toohey, M. & Sigl, M. Volcanic stratospheric sulfur injections and aerosol optical depth from 500 BCE to 1900 CE. Earth System Science Data 9, 809–831 (2017)

Please find below our detailed responses (in blue) to comments provided by Reviewers #1 and #2 The original reviewer comments are repeated here in black for clarity and completeness.

Reviewer #1

I have read the article with regard to the peer review of both reviewers and believe that the authors have worked conscientiously to refine the article and thereby meet the peer reviewers. The manuscript is very well written and the authors have greatly improved the clarity and strength of arguments and both detailed the methodology and clarified the statistics as requested. The interpretation of results and conclusions are supported by the data while at the same time the authors are well aware of the limitations of the study. I am convinced that this paper will increase interests in the role of volcanism when comes to explaining climate perturbations and recommend that the paper be accepted for publication.

We sincerely thank Reviewer #1 for taking the time to re-review our manuscript, as their comments have been really useful for improving it. Thus, we are thrilled they feel it is now suitable for publication pending minor revisions, which we address in turn below.

My only comment at this stage is a minor editing type of a comment:

L100 either skip (ka) where it is at the end of the sentence or move it to after kiloyears (ka): "presented herein as kiloyears (ka) before 1950 CE."

Good suggestion, this has now been corrected:

Line 100: "…*kiloyears (ka) before 1950 CE.*"

...and just some food for thoughts:
L305 in regard to the discussion that starts in L305 I think you can easily refer to Figure 3e as an example of stepwise cooling from 8.6 to 8.2 ka seen in the proxy record (MD99-2269 Quartz (%)). Another possible example also based on proxy data could be Larsen et al 2012 QSR 39 who identified two distinct and severe cold pulses between 8.7 and 8.0 and suggested additional trigger mechanisms, such as increased volcanism.

We agree that a reference to **Figure 3e** would be useful here, and so have amended the text:

Line 306: "…*latitude eruptions (**Figs. 3e,5**).*"

We also concur that the paper by Larsen et al. (2012) is also one that is relevant here, and indeed there are several papers that could be included to our discussion of the 8.2 ka event. To adhere to the reference/formatting requirements of *Nature Communications* we were unable to add this reference at this stage to the main manuscript, however, we have added it to our supplementary text:

"…increasingly support the proposition that explosive volcanism has repeatedly triggered annual-to-decadal scale cooling episodes throughout the Common Era (~2.5 ka to present)[11,18,23,24]."

Reviewer #2

The revised manuscript "Evidence For Volcanic Forcing of Holocene Cold Events" by Paine et al provides evidence for explosive volcanism initiation of centennial scale cold periods during the Holocene. The authors have diligently and thoughtfully addressed the reviewer comments, resulting in a clearer manuscript worthy of publication once some minor issues are addressed to the Editor's satisfaction.

Dr. Roberts' feedback has significantly improved the manuscript, so we are incredibly grateful to him for taking the time to review our manuscript, and delighted to hear he is happy for publication pending minor revisions.

The most significant remaining issue is inconsistency between the main text and Table S1.

We thank Dr. Roberts for pointing out these minor errors, and fully concur they require correction to ensure consistency between the manuscript and accompanying information. We highlight where these corrections have been made as follows:

- The text list n=11 for M>=7 (lines 126 and 429) but Table S1 only had 10 M>=7 eruptions, I think the problem is that Kuwae has been deleted from the table. If n=10 for M>=7 is correct, the statistical analysis for n=11 need to be redone for n=10

Dr. Roberts is right to point this out, as the discrepancy does appear misleading. It arises as we also included the following eruption in our statistical analysis:

Volcano	Latitude	Eruption Unit	M	^{14}C Age	Ref	IntCal20 Age*	Ice Core Age	Ref
Aniakchak	56.88	Aniakchak II	6.9	-	-	-	3578±4	1

It is true that the *Aniakchak II* eruption is listed in **Table S1** with a magnitude of 6.9 event, which corresponds to the magnitude calculated by (and listed in) the LaMEVE database[2]. Nonetheless, we decided to include it in our analysis for several reasons. First, it is one of only two eruptions in our compilation with a listed magnitude of 6.9, meaning it was already on the cusp of the M7 boundary. Second, the volcano is surrounded by ocean, meaning that this value likely constitutes a lower-end estimate of the true eruption size[3]. This uncertainty is confounded by parameters used in the volume calculation itself. The magnitude of this event calculated by LaMEVE (6.9) assumes a magma density of 2500 kg m^{-3}, and tephra density of 1000 kg m^3. However, evidence from analysis of the Aniakchak II eruption deposits suggest a magma density as low as 2200 kg m^{-3} [4], and evidence from eruptions with a similarly dacite-dominant composition have shown that tephra densities up to 1500 kg m^{-3} [5]. Thus, if the Aniakchak II eruption volumes are recalculated using these values, the resulting magnitude exceeds 7.0, and so in excess of our stated threshold.

To ensure full transparency and clarity in our reasoning, we add the following footnote to **Table S1**:

*"*Inclusion of Aniakchak II in our statistical analysis follows an independent assessment of all eruptions listed in Table S1, where each event was assessed in order to verify volumetric estimations and their associated magnitude calculations. Despite the Aniakchak II having a LaMEVE-listed magnitude that falls just below our M≥7 criterion, its inclusion is justified for two reasons. First, the volcano is surrounded by ocean, the reported eruption size of 6.9 is likely a minimum estimate[94]. Second, LaMEVE calculates a magnitude of 6.9 using magma and tephra densities of 2500 kg m^{-3} and 1000 kg m^{-3}, but*

using values more consistent with field evidence (2200 kg m^{-3} and 1500 kg m^{-3}, respectively [95,96]) would increase the estimated magnitude to above 7.0."

We also note that although the Okmok I eruption is also listed in VOGRIPA as a 6.9, we did not include this event in our analysis. This is due to greater discrepancies (and uncertainty) in the literature regarding it's magnitude, with values ranging from 6.5[6,7] - 7.0[8]; often tending toward the lower end of this range.

- The text lists a total of 50 (line 107, 367 and 372) eruptions, but (even without Kuwae) Table S1 lists 51 eruptions.

Well spotted. 51 is the correct number, and so has been amended in the main manuscript accordingly:

Line 107: "*…volume (n = 51; **Table S1**).*"

Line 366: "*Following stages one and two, 51 eruptions matched our selection criteria…*"

Line 371: "*From the 51 eruptions that remained in our compilation following stages (1) and (2)…*"

- The text lists n=18 for M>6.7 (line 144), but Table S1 only has 14 (or 15 if Kuwae is added) M>6.7

14 is the correct number, this has now been corrected in the manuscript:

Lines 144-145: "*…statistical significance is also maintained if all M > 6.7 eruptions (n = 14) were included in the analysis (p = 0.006).*"

- The test lists Kurile Lake as M7.3 and Tao-Rusyr as M7.1 (line 262), but Table S1 lists them as 7.2 and 7.0 respectively

Table S1 now checked, and magnitude values corrected:

Volcano	Latitude	Eruption Unit	M	^{14}C Age	Ref	IntCal20 Age*	Ice Core Age	Ref
Kurile Lake	51.45	CF	7.3	7618±14	[9]	8402±22	-	-
Tao-Rusyr	49.35	CF	7.0	7500±80	[10]	8308±140	-	-

Minor suggested topographical edits are listed below
- line 121 did you mean 'one year to a few decades'? i.e. add "year"

Corrected as follows:

Line 121: "*…time delays as low as one year, to several decades[17,40,44].*"

- Figure 3 caption, bold "(e)"

Done.

- line 280, you have already defined AMOC previously, so could change "Atlantic Meridional Overturning Circulation (AMOC)" to "AMOC"

Now corrected:

Line 238: "*…weakening of the AMOC…*"

I would like to finish by thanking the authors and editor for making reviewing this manuscript an enjoyable experience.

We echo this sentiment, and extend our thanks back to Dr Roberts.

References

1. Pearson, C. *et al.* Geochemical ice-core constraints on the timing and climatic impact of Aniakchak II (1628 BCE) and Thera (Minoan) volcanic eruptions. *PNAS Nexus* **1**, pgac048 (2022).
2. Crosweller, H. S. *et al.* Global database on large magnitude explosive volcanic eruptions (LaMEVE). *Journal of Applied Volcanology* **1**, 1–13 (2012).
3. Waythomas, C.F., Neal, C. A. Tsunami generation by pyroclastic flow during the 3500-year B.P. caldera-forming eruption of Aniakchak volcano, Alaska. *Bulletin of Volcanology* **60**, 110–124 (1998).
4. Bacon, C. R., Neal, C. A., Miller, T. P., McGimsey, R. G. & Nye, C. J. *Postglacial Eruptive History, Geochemistry, and Recent Seismicity of Aniakchak Volcano, Alaska Peninsula*. (2014) doi:http://dx.doi.org/10.3133/pp1810.
5. Paine, A. R. & Wadsworth, F. B. Large explosive eruptions may be dominated by pyroclastic flows instead of buoyant plumes: insights from a global data compilation. *J Appl. Volcanol.* **14**, 3 (2025).
6. Hayakawa, Y. Tephras of the World. (1996).
7. Larsen, J. F., Neal, C., Schaefer, J., Beget, J. & Nye, H. Late Pleistocene and Holocene Caldera-Forming Eruptions of Okmok Caldera, Aleutian Islands, Alaska. in *Volcanism and Subduction: The Kamchatka region* 343–364 (2007).
8. Decker, R. How often does a Minoan eruption occur? in *Thera and the Aegean World III. Volume Three: Chronology* 444–452 (Thera Foundation, London, 1990).
9. Ponomareva, V. V. *et al.* The 7600 (14C) year BP Kurile Lake caldera-forming eruption, Kamchatka, Russia: stratigraphy and field relationships. *Journal of Volcanology and Geothermal Research* **136**, 199–222 (2004).
10. Braitseva, O. A., Melekestsev, I. V., Ponomareva, V. V. & Sulerzhitsky, L. D. Ages of calderas, large explosive craters and active volcanoes in the Kuril-Kamchatka region, Russia. *Bulletin of Volcanology* **57**, 383–402 (1995).
